# CORO1C (coronin 1C) promotes autophagosome formation by coordinating branched actin network dynamics

**DOI:** 10.1080/15548627.2026.2658234

**Published:** 2026-04-20

**Authors:** Guozhong Zhang, Ningqing Yu, Yi Sun, Xiaowen Li, Lihong Sun, Guang Liu, Yue Huang

**Affiliations:** a State Key Laboratory of Common Mechanism Research for Major Diseases, Institute of Basic Medical Sciences & School of Basic Medicine, Chinese Academy of Medical Sciences & Peking Union Medical College, Beijing, China; b Department of Medical Genetics, Institute of Basic Medical Sciences & School of Basic Medicine, Chinese Academy of Medical Sciences & Peking Union Medical College, Beijing, China; c Center for Experimental Animal Research, Institute of Basic Medical Sciences & School of Basic Medicine, Chinese Academy of Medical Sciences & Peking Union Medical College, Beijing, China; d McKusick-Zhang Center for Genetic Medicine, Institute of Basic Medical Sciences & School of Basic Medicine, Chinese Academy of Medical Sciences & Peking Union Medical College, Beijin, China

**Keywords:** ARP2/3 complex, autophagy, branched actin network, coronin 1C, genetic screen, SQSTM1/p62 body formation

## Abstract

Macroautophagy/autophagy is a critical cellular process that maintains the cellular homeostasis by degrading and recycling cytotoxic material. Despite its importance, the intricate mechanisms governing this process remain partially elusive. Here, we designed and performed a genome-wide loss-of-function screen on a mouse haploid ESC mutant library and identified the actin-binding protein CORO1C (coronin 1C) as a previously unrecognized regulator of mammalian autophagy. Interactions between CORO1C and the ACTR2/ARP2 (actin related protein 2)-ACTR3/ARP3 complex are essential for branched actin network assembly, SQSTM1/p62 body formation, and maintaining autophagosome structural integrity. Unlike CORO1A and CORO1B, CORO1C possesses a unique second actin-binding site involved in regulating the branched actin network and autophagic process. Notably, *coro1c*^−/−^ newborn mice died earlier in starvation than wild-type littermates and multiple tissues showed autophagy-deficient phenotypes. Moreover, the adult *coro1c*-deficient mice exhibit severe spatial learning memory impairment. Collectively, our research uncovered the surprising role of CORO1C in promoting the formation of branched actin network and its central role in the assembly of structures vital to autophagy.

**Abbreviations**: ACTR2/ARP2: actin related protein 2; ACTR3/ARP3: actin related protein 3; ARPC2: actin related protein 2/3 complex, subunit 2; ATG: autophagy related; ATG5: autophagy related 5; BafA1: bafilomycin A_1_; CQ: chloroquine; FACS: fluorescence-activated cell sorting; GAPDH: glyceraldehyde-3-phosphate dehydrogenase; haESC: haploid embryonic stem cell; HML: haploid-mutant library; IF: immunofluorescence; KO: knockout; MAP1LC3B/LC3: microtubule-associated protein 1 light chain 3B; RB1CC1/FIP200: RB1-inducible coiled-coil 1; SQSTM1/p62: sequestosome 1; STX17: syntaxin 17; TEM: transmission electron microscopy; WB: western blotting; WT: wild type

## Introduction

Macroautophagy (herein referred to as autophagy) is an evolutionarily conserved intracellular degradation process present in all eukaryotic organisms. This intricate, multi-step process is regulated by over 40 core *ATG* (autophagy related) genes [1]. The evolution of autophagy research is closely linked to the identification of these *ATG*s, initially discovered using genetic screenings in *Saccharomyces cerevisiae* [2,3] and *Caenorhabditis elegans* [4]. In recent years, advancements in RNA interference (RNAi) [5] and CRISPR screening [6,7] have led to the identification of numerous novel factors involved in autophagy within mammals. For example, the inhibitor of apoptosis protein BIRC6 (baculoviral IAP repeat-containing 6) has been shown to enhance autolysosome formation, a function that is distinct from its role in ubiquitin conjugation [5]. Additionally, UBA6 (ubiquitin like modifier activating enzyme 6) and BIRC6 have been found to exert a negative regulatory effect on autophagy through the ubiquitination of MAP1LC3B/LC3 (microtubule-associated protein 1 light chain 3B) [7]. And a CRISPR screen has shown that TMEM41B (transmembrane protein 41B) is a novel ER-localized regulator of autophagosome formation [6]. These discoveries continue to expand our understanding of the complex machinery underlying autophagy.

Although the molecular roles and underlying mechanisms of these ATG proteins have been comprehensively investigated, several important aspects of this dynamic process in mammals, such as the autophagosome biogenesis and the formation of SQSTM1/p62 (sequestosome 1) bodies, remain largely unclear. The important role of the actin cytoskeleton in autophagy has been highlighted in several studies. For example, the branched actin network was found to serve as a cytoskeletal frame inside the phagophore, participating in its expansion and shaping. Indeed, a knockdown of the barbed-end capping protein CapZ or inhibition of the ACTR2/ARP2 (actin related protein 2)-ACTR3/ARP3 (actin related protein 3) (ARP2/3) complex results in the collapse of phagophore [1,8]. Additionally, a study reported that myosin 1D and the branched actin network control the condensation of the SQSTM1/p62 bodies [9], although the details of this regulation are largely unknown. For example, it is unclear how the actin cytoskeleton is recruited and assembles on membranes during autophagy, and whether there are other unknown regulators involved in the actin- associated autophagic process.

Given the highly complex and precise regulation of autophagy, it is likely that additional autophagy-related factors need to be identified. Because haploid embryonic stem cells (haESCs) possess only one set of chromosomes, they have rapidly emerged as a powerful tool for forward genetic screening and have been successfully utilized in numerous genetic screening efforts [10–14].

In this work, we designed and conducted a genome-wide loss-of-function screen on a mouse haESC mutant library and identified CORO1C (coronin 1C) as a novel autophagy regulator. CORO1C, but not CORO1A and CORO1B, functioned with the ARP2/3 complex to promote the formation of the branched actin network (a function that is distinct from the known role of CORO1C as a destabilizer), which was required for the formation of autophagosomes and SQSTM1/p62 bodies. These findings highlight the critical function of the CORO1C-dependent branched actin network in autophagy-related structure formation, offering new insights into the regulation of autophagy.

## Results

### Genome-wide loss-of-function screens in mouse haESCs to identify key regulators of autophagy

**Figure 1.**
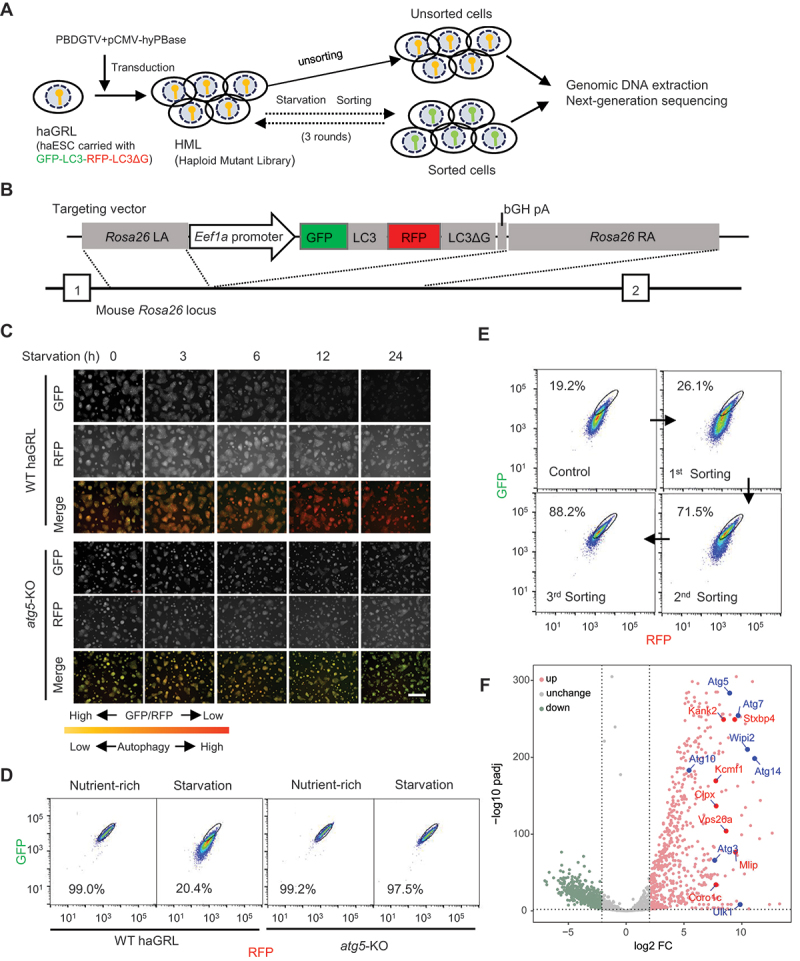
Genome-wide loss-of-function screen for genes regulating autophagy in mouse haESCs. (**A**) Schematic representation of the genetic screening procedure. The haploid mutant library (HML) was generated by transfecting cells with the PB transposon vector PBDGTV and the pCMV-hyPBase transposase plasmid, leading to genome-wide insertional mutations. Cells in the HML exhibiting a high GFP:RFP ratio under starved conditions were sorted and cultured through three rounds. Genomic DNA was extracted from both unsorted and sorted cells, followed by analysis using next-generation sequencing. (**B**) Schematic representing GFP-LC3-RFP-LC3ΔG insertion into the *Rosa26* locus of haESC to generate autophagic flux reporter cell lines. The boxes in the *Rosa26* locus represent gene exon. (**C**) WT and *atg5*-KO haGRL cells were starved for the indicated times and analyzed by fluorescence microscopy. In the merged images, the yellow signal indicates a high GFP:RFP ratio and a low autophagic flux, whereas the red signal represents a low GFP:RFP ratio and a high autophagic flux. (**D**) WT and *atg5*-KO haGRL cells were treated with nutrient-rich or starvation condition and analyzed by FACS. The proportion (%) of autophagy-deficient cells is indicated by the region of interest (ROI). (**E**) FACS analysis of the cells in HMLs during three rounds of sorting. The percentage of autophagy-deficient cells is represented by the ROI. (**F**) Volcano plots representing foldchange of candidate genes. For more detailed information about candidate genes, please see Table S1.

To uncover new key regulators of autophagy, we performed a comprehensive screen utilizing a genome-wide haploid mutant library (Figure 1A). First, a dual-fluorescent reporter for monitoring autophagic flux, GFP-LC3-RFP-LC3ΔG [15], was integrated into the *Rosa26* locus in mouse OdG haESC line [16], generating a new cell line designated as haGRL (**ha**ESCs expressing **G**FP-LC3-**R**FP-**L**C3ΔG) (Figure 1B ; Figure S1A). In an autophagy process, the GFP-LC3-RFP-LC3ΔG fusion protein is cleaved by endogenous ATG4 protein to produce equal amounts of GFP-LC3 and RFP-LC3ΔG. The GFP-LC3 is conjugated to phosphatidylethanolamine on autophagic membranes and is then transported to the lysosome, where the GFP signal is quenched. Meanwhile, the RFP-LC3ΔG stays in the cytosol and serves as an internal control. Therefore, assessing the GFP:RFP ratio using flow cytometry enables the visualization of the autophagic flux.

PCR assays showed that the reporter system was correctly targeted at the *Rosa26* locus of haGRL, and flow cytometry showed that most cells in this population remained in a haploid state (**Figure S1B, C**). Upon nutrient deprivation, the GFP signal in haGRL cells decreased as expected, while the RFP signal remained stable (Figure 1C, D). The depletion of *Atg5* (autophagy related 5) gene (**Figure S1D, E**), a critical component of autophagy [17], resulted in a significant attenuation of the decreased GFP levels induced by starvation (Figure 1C, D; Figure S1F). These findings confirmed that the reporter system employed in our study could effectively detect both normal and disrupted autophagic flux in haESCs.

Next, the haGRL cells and gene-trap vector *PiggyBac*-based Dual Gene Trap Vector (PBDGTV) [18] were utilized to create a genome-wide mutant library designated as the Haploid Mutant Library (HML), which comprising approximately 13,000 mutant genes (**Figure S1G**). A ploidy analysis of the HML using flow cytometry confirmed that the majority of the mutant cells maintained their haploid state (**Figure S1H**). Furthermore, a three-primers PCR revealed that 14 out of 16 individual cell clones (over 87%) contained homozygous insertions (**Figure S1I**), signifying the successful construction of a genome-wide homozygous mutant library.

For the screen, cells in the HML were starved for 24 h and then subjected to fluorescence-activated cell sorting (FACS) in order to isolate the cells exhibiting impaired autophagic flux. After three cycles of sorting and culturing, the proportion of autophagy-defective cells rose from 19.2% to 88.2% (Figure 1E). Genomic DNA was extracted from both unsorted and sorted HML cells for the analysis of the distribution of transposon insertions. Candidate autophagy-regulating genes were identified using Splinkerette-PCR coupled with next-generation sequencing (Figure 1F ; Table S1). The identification of key mammalian autophagy genes, including *Atg3*, *Atg5*, *Atg7*, *Atg10*, *Atg14*, *Ulk1* and *Wipi2*, as potent regulators of autophagy in our screen validated the efficacy of our approach.

### CORO1C is a novel autophagy regulator and is essential for the formation of SQSTM1/p62 bodies

Next, we selected seven candidate genes, *Clpx*, *Coro1c*, *Kank2*, *Kcmf1*, *Mlip*, *Stxbp4* and *Vps26a* for further investigation. The cells bearing the respective gene knockout (KO) were generated individually using the CRISPR-Cas9 system in a widely used mouse diploid ESC line (AB2.2 carrying a GRL reporter). The deletion of five candidate genes, including *Coro1c*, *Kank2*, *Kcmf1*, *Mlip* and *Vps26a* resulted in impaired autophagy flux in KO cells (**Figure S2A, B**). Notably, concurrent with our findings, another study reported that *Kcmf1* regulates autophagy in renal cell carcinoma [19]. To exclude the effect of the genetic background, we deleted the *CORO1C*, *KANK2*, *MLIP* and *VPS26A* genes individually in HEK293FT (293 FT) cells carrying a GRL reporter (293 FT GRL). FACS showed that the *CORO1C-*KO and *MLIP*-KO cells could inhibit the reduction in the GFP signal after starvation compared with that in WT cells, and that the *CORO1C* phenotype was the most pronounced (**Figure S2C, D**). Consequently, we focused on *CORO1C* for an in-depth investigation.

**Figure 2.**
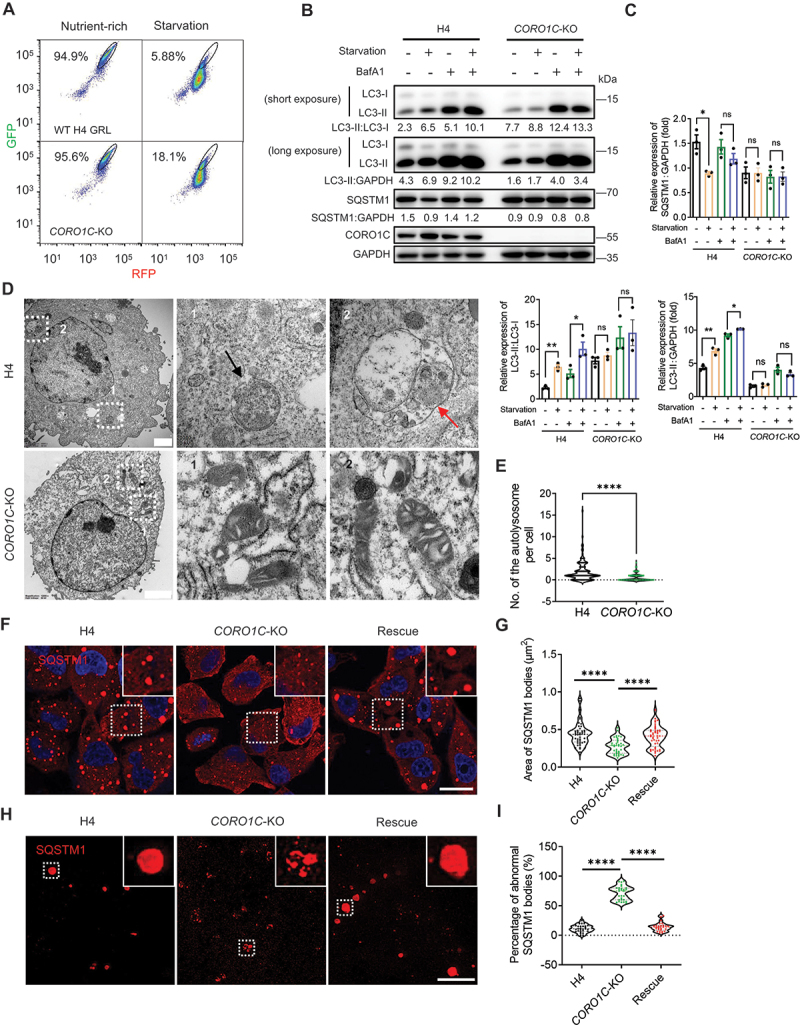
CORO1C is a novel autophagy regulator and is essential for the formation of SQSTM1/p62 bodies. (**A**) WT and *CORO1C*-KO H4GRL cells were treated with nutrient-rich or starvation condition for 24 h, followed by flow cytometry analysis; (**B**) WB analysis in wt and *CORO1C*-KO H4 cells cultures in nutrient-rich or starvation conditions with or without BafA1 (100 nM) for 4 h. (**C**) Quantification of the SQSTM1:GAPDH, LC3-II:LC3-I and LC3-II:GAPDH ratios from (b). (**D**) Representative TEM images of autophagosomes and autolysosomes in wt and *CORO1C*-KO H4 cells after 4 h of starvation. Scale bar: 2 μm. The autophagosome and autolysosome were indicated with black and red arrowhead, respectively. (**E**) Number of autolysosomes per cell from (D) was quantified. *n* = 124 (H4) and 102 (*CORO1C*-KO) cells were analyzed in three independent experiments. (**F**) Cells were starved for 4 h and then stained with an antibody against SQSTM1/p62 for immunofluorescence microscopy. DAPI was used to stain the nucleus (blue). Regions outlined with white dashed lines are magnified in the insets. Scale bar: 20 μm. (**G**) The size of SQSTM1/p62 bodies in (F) was quantified. *n* = 44 (H4), 38 (*CORO1C*-KO) and 51 (rescue) cells were assessed from two independent experiments. (**H**) Cells were starved for 4 h and then stained with an antibody against SQSTM1/p62 for super-resolution fluorescence microscopy. Scale bar: 5 μm. (**I**) The percentage of abnormal SQSTM1/p62 bodies in cells from (H) was quantified. *n* = 27 (H4), 28 (*CORO1C*-KO) and 26 (rescue) cells were assessed from two independent experiments. Data are presented as mean ± SEM. Unpaired t-test for B, d, F, H and J; ns, not significant, *** *p* < 0.001, **** *p* < 0.0001.

To characterize the role of *CORO1C* in autophagy, we deleted the *CORO1C* gene in the H4 (human neuroglioma) and H4 GRL cell lines (**Figure S2E**). The absence of *CORO1C* resulted in pronounced autophagy defects in the H4GRL cells, as evidenced by the markedly suppressed degradation of GFP under starvation conditions (Figure 2A). Western blotting (WB) further confirmed the presence of these defects, revealing a disruption in autophagy in the *CORO1C*-KO H4 cells. Under starvation conditions, wild type (WT) H4 cells exhibited a characteristic increase in the LC3-II:LC3-I ratio, consistent with normal autophagic flux. In contrast, *CORO1C*-KO cells displayed two key abnormalities: first, they maintained constitutively lower levels of LC3-I under normal or starved conditions. Second, and more strikingly, the starvation-induced increase in the LC3-II:LC3-I and LC3-II:GAPDH ratios was severely blunted, even upon bafilomycin A_1_ (BafA1) treatment (Figure 2B). Additionally, the degradation of SQSTM1/p62, a key autophagy substrate, was significantly impaired in the *CORO1C*-KO cells (Figure 2B). Interestingly, treatment with BafA1 resulted in weaker accumulation of total SQSTM1/p62 protein compared to LC3-II in both WT and *CORO1C*-KO cells. This differential response could be due to the intrinsically slower turnover of SQSTM1/p62 relative to LC3-II [20,21], or it may indicate that total SQSTM1/p62 levels are not a reliable proxy for autophagic flux, especially if downstream degradation is compromised [9]. Using transmission electron microscopy (TEM), we observed that the *CORO1C*-KO cells exhibited a reduced abundance of autophagosomes and autolysosomes when compared with those in WT cells (Figure 2C,D).

Immunofluorescence (IF) analysis showed that SQSTM1/p62 bodies in the WT cells formed a large spherical structure, while the number and area of SQSTM1/p62 bodies in *CORO1C*-KO cells had decreased significantly (Figure 2E,F). Furthermore, we found that SQSTM1/p62 had dispersed into numerous smaller puncta and showed irregular shapes in the *CORO1C*-KO cells by using super-resolution fluorescence microscopy, indicating a defect in the aggregation of SQSTM1/p62 bodies (Figure 2G,H). To verify that the deletion of *CORO1C* was responsible for the defective autophagy, we established a rescue cell line by stably expressing *CORO1C* in *CORO1C*-KO cells (designated as the rescue cell line) (**Figure S2F**). The results show that the defective phenotypes could be reversed through ectopic expression of *CORO1C* gene (Figure 2E–H). In summary, these data underscored the essential role of CORO1C in autophagy, and its critical involvement in the formation and maintenance of SQSTM1/p62 bodies.

### CORO1C deficiency impairs the formation of the phagophore and autophagosome

**Figure 3.**
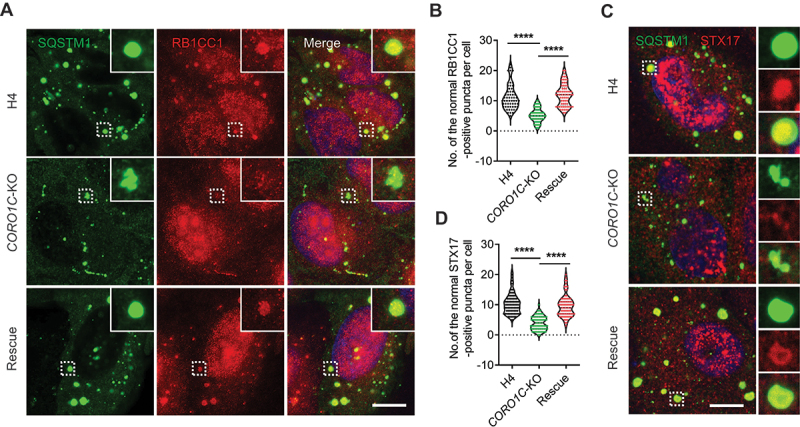
*CORO1C* deficiency impairs the formation of phagophore and autophagosomes. (**A**) Representative confocal image of wt, *CORO1C*-KO and rescued H4 cells immunolabeled with anti-SQSTM1/p62 and anti-RB1CC1 after starved for 4 h. DAPI was used to stain the nucleus (blue). Regions outlined with white dashed lines are magnified in the insets. Scale bar: 10 μm. (**B**) Cells from (A) were quantified for normal RB1CC1-positive puncta. *n* = 60 (H4), 67 (*CORO1C*-KO) and 69 (rescue) cells were assessed from three independent experiments. A structure with a diameter greater than 0.9 μm and a rounded shape is defined as a normal structure. (**C**) Representative confocal images of wt, *CORO1C*-KO and rescue H4 cells immunolabeled with anti-SQSTM1/p62 and anti-STX17 after starved for 4 h. DAPI was used to stain the nucleus (blue). Regions outlined with white dashed lines are magnified to right. Scale bar: 10 μm. (**D**) Cells from (C) were quantified for normal STX17-positive puncta. *n* = 81 (H4), 73 (*CORO1C*-KO) and 80 (rescue) cells were assessed from three independent experiments. A structure with a diameter greater than 0.9 μm and a rounded shape is defined as a normal structure. Data are presented as mean ± SEM. Unpaired t-test for B and D; **** *p* < 0.0001.

In order to assess the effects of a *CORO1C* deficiency on the autophagic process, we utilized RB1CC1/FIP200 (RB1-inducible coiled-coil 1), LC3 and STX17 (syntaxin 17) as markers. RB1CC1 is present on the phagophore [22], LC3 is localized on autophagosomes [23], and STX17 is specifically found on mature autophagosomes [24]. Using confocal fluorescence microscopy, we observed a significant decrease in the number of ring-shaped RB1CC1-positive puncta in the *CORO1C*-KO H4 cells under starvation conditions, with a corresponding change in the size and shape of these puncta to smaller and irregular forms (Figure 3A,B). Consistent with the results obtained for RB1CC1, the number of ring-shaped LC3-positive (**Figure S3A, B**) or STX17-positive puncta (Figure 3C,D) was obviously decreased in the *CORO1C*-KO H4 cells, and these puncta showed small clusters or irregular shapes. Moreover, the defective phenotypes observed in the *CORO1C*-KO H4 cells were reversible in rescue cells in which *CORO1C* was re-expressed ectopically (Figure 3A–D ; Figure S3A, B). Collectively, these results indicated that CORO1C is required for the formation of both the phagophore and autophagosome in human cells.

### CORO1C depletion disrupts the formation of the branched actin network within autophagy-related structures

**Figure 4.**
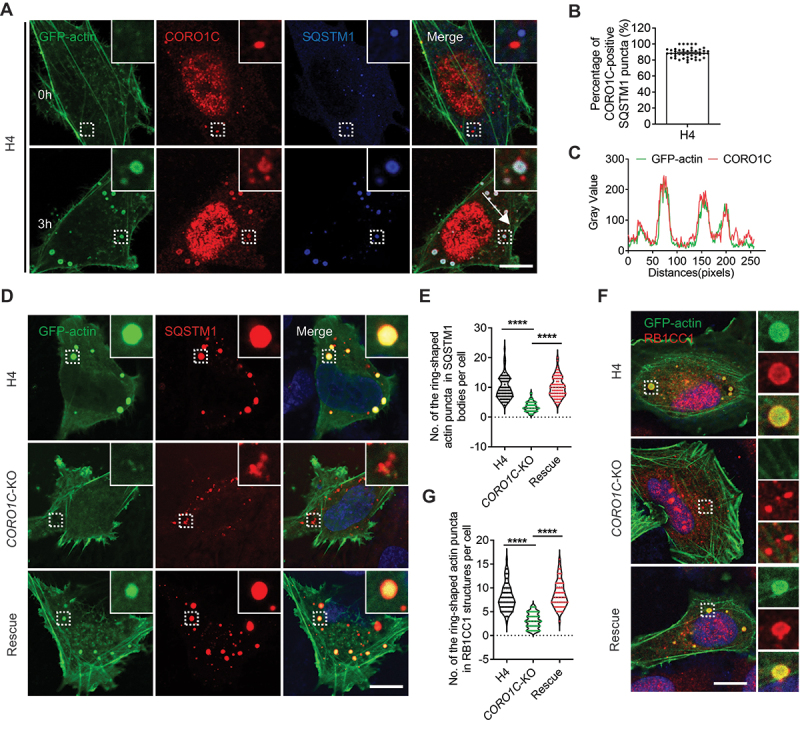
CORO1C facilitates the formation of the branched actin network in autophagy-related structures. (**A**) H4 cells were transfected with GFP-actin, starved for 0 or 3 h and then stained with an antibody against SQSTM1/p62 and CORO1C. Regions outlined with white dashed lines are magnified in the insets. Scale bar: 10 μm. (**B**) The percentage of CORO1C-positive SQSTM1/p62 puncta was quantified in cells from (A) (*n* = 46, cells were assessed from two independent experiments). (**C**) Fluorescent intensity of GFP-actin and CORO1C signals across indicated region marked with arrow from (A) is shown in line plots. (**D**) Representative confocal images of wt, *CORO1C*-KO and rescued H4 cells transfected with GFP-actin and immunolabeled with anti-SQSTM1/p62 after starved for 4 h. DAPI was used to stain the nucleus (blue). Regions outlined with white dashed lines are magnified in the insets. Scale bar: 10 μm. (**E**) The number of ring-shaped actin puncta in SQSTM1/p62 bodies was quantified in cells from (D). *n* = 79 (H4), 76 (*CORO1C*-KO) and 79 (rescue) cells were assessed from two independent experiments. (**F**) Representative confocal images of wt, *CORO1C*-KO and rescued H4 cells transfected with GFP-actin and immunolabeled with anti-RB1CC1 after starved for 4 h. DAPI was used to stain the nucleus (blue). Regions outlined with white dashed lines are magnified to right. Scale bar: 10 μm. (**G**) The number of ring-shaped actin puncta in RB1CC1 structures was quantified in cells from (F). *n* = 64 (H4), 59 (*CORO1C*-KO) and 62 (rescue) cells were assessed from two independent experiments. Data are presented as mean ± SEM. Unpaired t-test for E and G; **** *p* < 0.0001.

After establishing the significance of CORO1C in autophagy, we aimed to elucidate the mechanism underlying CORO1C-mediated regulation of the formation of SQSTM1/p62 bodies and autophagosomes. Prior studies have shown that CORO1C is an actin-binding protein [25], and the branched actin network is crucial for the formation of SQSTM1/p62 bodies [9]. This prompted us to investigate whether the abnormality in the SQSTM1/p62 bodies of the *CORO1C*-KO H4 cells could be attributed to changes in the branched actin network. We found that actin showed a filamentous morphology when H4 cells were cultured under nutrient-rich conditions; a few small CORO1C and SQSTM1/p62 puncta were observed but did not colocalize (Figure 4A). After a 3-h starvation, actin-positive puncta began to appear in starved cells. Large and ring-shaped CORO1C puncta and SQSTM1/p62 bodies were also observed, and all three puncta showed a colocalized pattern (Figure 4A–C). Previous studies have reported that actin puncta contain branched actin networks [8], our results confirmed this previous finding, as we showed that the actin puncta colocalized with CTTN (cortactin) (**Figure S4A, B**), a protein known to stabilize and serve as a marker for the branched actin network [26]. In WT H4 cells, actin puncta colocalized with SQSTM1/p62 bodies under starvation conditions (Figure 4 4D,E). In contrast, *CORO1C*-KO cells exhibited a marked reduction in the number of ring-shaped actin puncta, accompanied by abnormal SQSTM1/p62 body morphology. Conversely, the actin puncta were restored and displayed colocalization with SQSTM1/p62 bodies in rescue cells, which exhibited a normal morphology similar to that of the WT H4 cells (Figure 4 4D,E). Importantly, the lack of normal actin puncta in the *CORO1C*-KO cells was not attributable to decreased level of the actin protein, as confirmed by WB (**Figure S4C, D**). These data suggested that CORO1C is crucial for the formation of the branched actin network and plays a pivotal role in the assembly of SQSTM1/p62 bodies.

Since the assembly of actin within the phagophore governs the formation of the autophagosomal membranes [1,8], we postulated that the absence of the branched actin network may be responsible for the impaired formation of phagophore and autophagosomes in the *CORO1C*-KO cells. In WT H4 cells, actin puncta were found to colocalize with the RB1CC1-positive puncta. Conversely, normal actin puncta were rarely observed in the RB1CC1 structures, and the size of RB1CC1 structures was reduced in the *CORO1C*-KO cells. Furthermore, actin puncta reemerged within the RB1CC1 structures, and the normal morphology of the RB1CC1 puncta was restored in rescue cells (Figure 4F,G). Notably, the STX17 (**Figure S4E, F**) and LC3 (**Figure S4G, H**) phenotypes closely resembled that of RB1CC1. In summary, these data indicated that the CORO1C is required for the autophagy, through the facilitation of branched actin network formation within autophagy-related structures, including SQSTM1/p62 bodies, phagophores and autophagosomes.

### CORO1C functions with ARP2/3 complex to promote the formation of branched actin network

**Figure 5.**
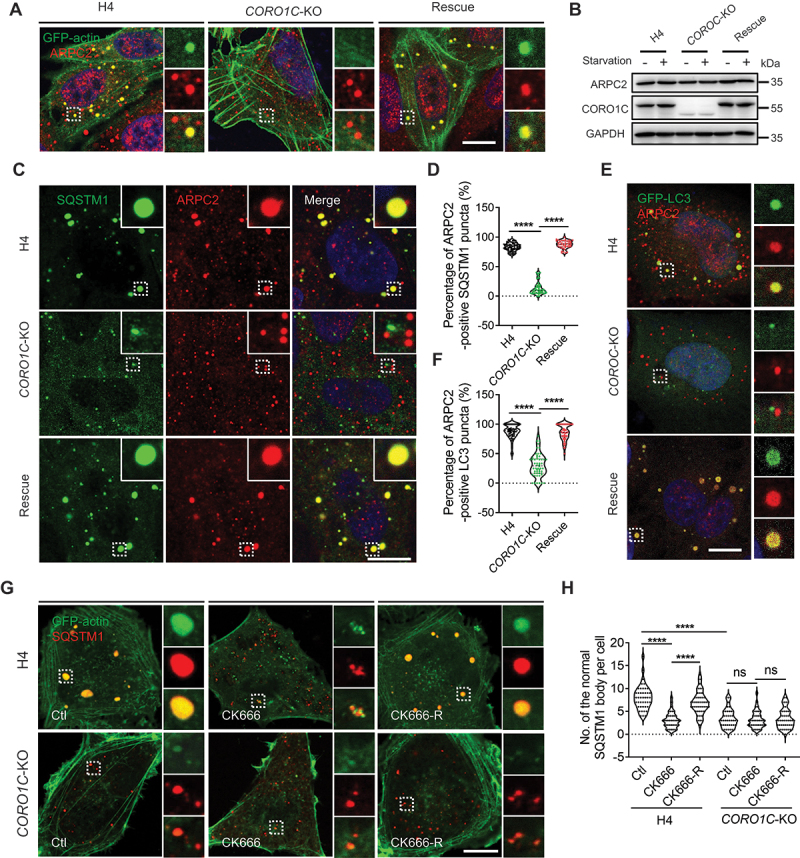
CORO1C functions with ARP2/3 complex to promote the formation of branched actin network. (**A**) Representative confocal images of wt, *CORO1C*-KO and rescue cells were transfected with GFP-actin and immunolabeled with anti-ARPC2 after starved for 4 h. DAPI was used to stain the nucleus (blue). Regions outlined with white dashed lines are magnified to right. Scale bar: 10 μm. (**B**) WB analysis in indicated cells cultured in nutrient-rich or starvation conditions for 4 h. (**C**) Representative confocal image of wt, *CORO1C*-KO and rescued H4 cells immunolabeled with anti-SQSTM1/p62 and anti-ARPC2 after starved for 4 h. DAPI was used to stain the nucleus (blue). Regions outlined with white dashed lines are magnified in the insets. Scale bar: 10 μm. (**D**) Cells from (C) were quantified for ARPC2-positive SQSTM1/p62 bodies. *n* = 65 (H4), 55 (*CORO1C*-KO) and 47 (rescue) cells were assessed from three independent experiments. (**E**) Representative confocal image of wt, *CORO1C*-KO and rescued H4 cells were transfected with GFP-LC3 and immunolabeled with anti-ARPC2 after starved for 4 h. DAPI was used to stain the nucleus (blue). Regions outlined with white dashed lines are magnified to right. Scale bar: 10 μm. (**F**) Cells from (e) were quantified for ARPC2-positive LC3 puncta. *n* = 58 (H4), 54 (*CORO1C*-KO) and 58 (rescue) cells were assessed from three independent experiments. (**G**) Reversible effect of CK666 on the formation of actin puncta and SQSTM1/p62 bodies. Cells were transfected with GFP-actin and treated without (Ctl) or with CK666 (100 μM) for 6 h. Cells treated by CK666 were then washed with DPBS three times for one minute each, and recovered with full medium 6 h (CK666-R). After each treatment, cells were stained with an antibody against SQSTM1/p62. Regions outlined with white dashed lines are magnified to right. Scale bar: 10 μm. (**H**) The number of ring-shaped SQSTM1/p62 bodies was quantified in cells from (G). *n* > 40 cells were assessed from two independent experiments. Data are presented as mean ± SEM. Unpaired t-test for d, F and H; * *p* < 0.05, ** *p* < 0.01, **** *p* < 0.0001; ns, not significant.

Given the absence of actin puncta in the *CORO1C*-KO H4 cells, we next investigated whether the function of CORO1C was linked to the ARP2/3 complex, which is the primary molecular machinery responsible for creating branched actin networks [27]. In H4 cells, nearly all actin puncta were found to colocalize with the ARP2/3 complex, as detected using ARPC2 (actin related protein 2/3 complex subunit 2). However, despite the unchanged structure of ARP2/3 complex (Figure 5A) and the consistent protein expression level of ARPC2 (Figure 5B), actin puncta were almost undetectable in *CORO1C*-KO cells. Confocal microscopy analysis demonstrated that in H4 cells, both ARPC2 and SQSTM1/p62 bodies formed large, round spots and colocalized with each other. Conversely, in *CORO1C*-KO cells, SQSTM1/p62 was restricted to forming small structures and failed to colocalize with ARPC2 spots (Figure 5C,D). The exogenous expression of *CORO1C* could restore the structure of SQSTM1/p62 bodies and reestablished their colocalization with ARPC2 (Figure 5C,D). Moreover, the phenotypic characteristics of ARPC2 and autophagosomes (Figure 5E,F; **Figure S5A, B**) were analogous to those observed in ARPC2 and SQSTM1/p62 bodies. These finding suggest that CORO1C plays a crucial role in the formation of SQSTM1/p62 bodies and their colocalization with the ARP2/3 complex.

The inhibitor CK666, known for its ability to inhibit the ARP2/3 complex and actin polymerization [28], disrupted the formation of actin puncta, resulting in a notable decrease in the presence of ring-shaped SQSTM1/p62 bodies (Figure 5G,H). Moreover, both the actin puncta and SQSTM1/p62 bodies reappeared after CK666 was washed off, underscoring the important role of the ARP2/3 complex-derived branched actin network in the regulation of autophagy (Figure 5G,H). The abnormal phenotypes of the actin puncta and SQSTM1/p62 bodies in the H4 cells treated with CK666 were similar to the phenotypes observed in the *CORO1C*-KO H4 cells. Notably, treatment of *CORO1C*-KO H4 cells with CK666 did not further disrupt the formation of actin puncta and SQSTM1/p62 bodies (Figure 5G,H). These results implied that CORO1C and the ARP2/3 complex may have coordinately facilitated the formation of the branched actin network during autophagy.

### Among type-1 coronins, only CORO1C regulates autophagy

**Figure 6.**
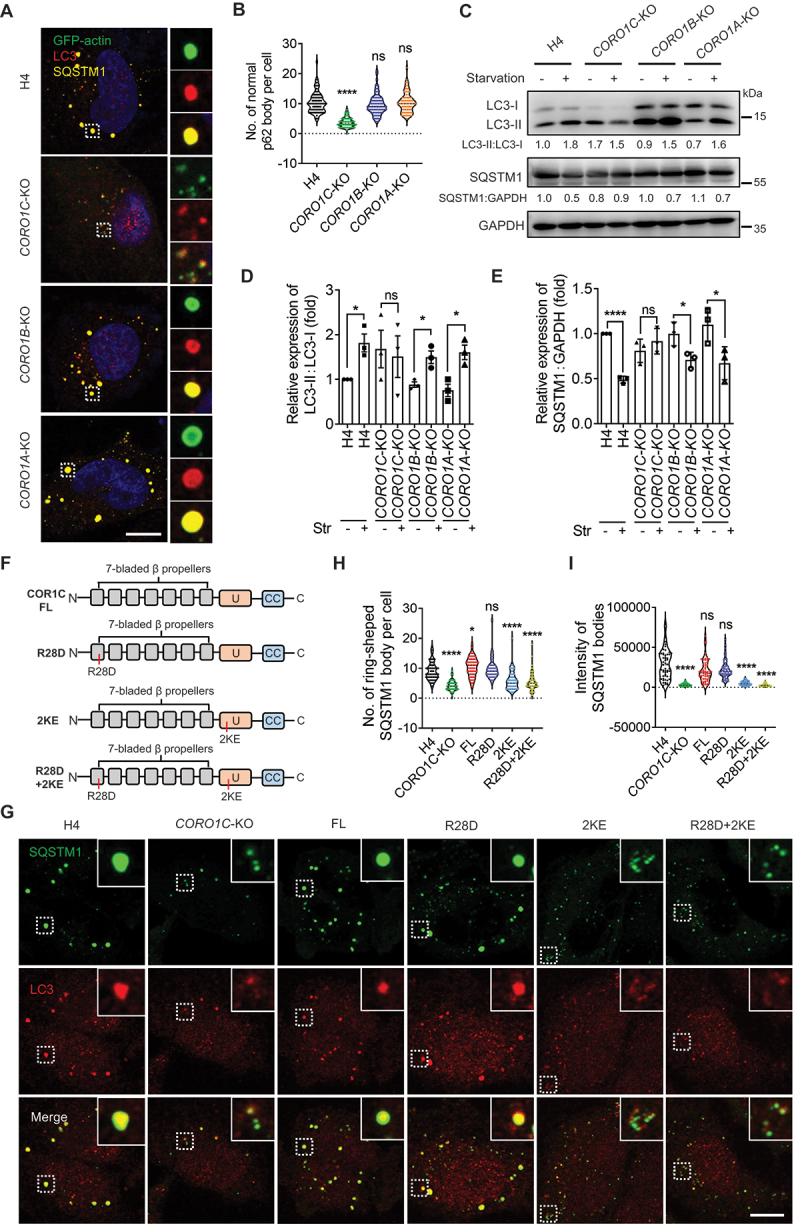
The second actin-binding site of CORO1C is required for the branched actin network formation and autophagy regulation. (**A**) Representative confocal images of wt, *CORO1C*-KO, *CORO1B*-KO and *CORO1A*-KO cells transfected with GFP-actin and immunolabeled with anti-SQSTM1/p62 and anti-LC3 after starved for 4 h. DAPI was used to stain the nucleus (blue). Regions outlined with white dashed lines are magnified to right. Scale bar: 10 μm. (**B**) Cells from (a) were quantified for number of ring-shaped SQSTM1/p62 bodies. *n* = 76 (H4), 80 (*CORO1C*-KO), 79 (*CORO1B*-KO) and 75 (*CORO1A*-KO) cells were assessed from two independent experiments. (**C**) WB analysis in wt, *CORO1C*-KO, *CORO1B*-KO and *CORO1A*-KO H4 cells cultures in nutrient-rich or starvation conditions for 4 h. (**D, E**) Quantitation of protein signal intensities from WB in (C) showing the ratio of LC3-II:LC3-I (D) and SQSTM1:GAPDH (E) (*n* = 3). (**F**) Domain diagrams of the mutations made to test domain functionality in CORO1C. (**G**) Cells were starved for 4 h and then stained with an antibody against SQSTM1/p62 and LC3. Regions outlined with white dashed lines are magnified in the insets. Scale bar: 10 μm. (**H**) Cells from (G) were quantified for number of SQSTM1/p62 bodies. *n* = 60 (H4), 72 (*CORO1C*-KO), 63 (FL), 53 (R28D), 79 (2KE) and 72 (R28D + 2KE) cells were assessed from two independent experiments. (**I**) Cells from (G) were quantified for intensity of SQSTM1/p62 bodies. *n* = 53 (H4), 72 (*CORO1C*-KO), 57 (FL), 60 (R28D), 73 (2KE) and 79 (R28D + 2KE) cells were assessed from two independent experiment. Data are presented as mean ± SEM. Unpaired t-test for d, e, H and I; * *p* < 0.05, ** *p* < 0.01, **** *p* < 0.0001; ns, not significant.

As mammalian type-1 coronins include *CORO1A*, *CORO1B*, and *CORO1C*, it prompted us to explore whether *CORO1A* and *CORO1B* regulate autophagy in a manner similar to *CORO1C*. First, we quantified the mRNA levels of these genes in various cell types. Our results reveal that CORO1B (encoded by the *CORO1B* gene) was expressed 0.3 to 0.6 times the level of CORO1C, while CORO1A (encoded by the *CORO1A* gene) exhibited very low expression levels in H4, 293 FT (**Figure S6A**) and mouse ES cells (**Figure S6B**). The *CORO1A*-KO and *CORO1B-*KO H4 and 293 FT cell lines were generated using the CRISPR-Cas9 system (**Figure S6C, D**). Confocal fluorescence microscopy revealed that unlike CORO1C, the actin puncta displayed normal morphology in the *CORO1A*-KO and *CORO1B-*KO H4 cells (Figure 6A,B). In addition, the formation of autophagosomes and SQSTM1/p62 bodies in the *CORO1A*-KO and *CORO1B-*KO H4 cells remained unaffected due to the normal formation of the branched actin network (Figure 6A,B). Furthermore, LC3 turnover and SQSTM1/ p62 degradation were similar in WT and *CORO1A*-KO or *CORO1B-*KO H4 cells (Figure 6C–E). The results of WB in 293 FT cells mirrored those obtained in H4 cells (**Figure S6E-H**). These data suggested that deficiencies in *CORO1A* or *CORO1B* did not impair the formation of the branched actin network during the autophagic process, highlighting the unique role of CORO1C in autophagy.

### The second actin-binding site of CORO1C is required for the branched actin network formation and autophagy regulation

We then questioned why CORO1C, rather than CORO1A or CORO1B, regulated the autophagic process, and what might be the mechanism underlying this regulatory activity. Although the expression patterns of type 1 coronins differ, all members share several characteristic domains: an N-terminal of the WD40 repeats domain contains the seven-bladed β propellers, a unique domain, and a C-terminal coiled-coil domain [29]. It has been reported that F-actin binding is essential for coronin function, with a specific binding site located in the β-propeller of all type 1 coronins (R29 in CORO1A, R30 in CORO1B, and R28 in CORO1C) [30,31]. However, CORO1C possesses a second actin-binding site within its unique domain [31]. Unlike CORO1A and CORO1B, CORO1C displays a highly co-operative binding to actin filaments [31]. This raised the question of whether the second actin-binding site of CORO1C plays a critical role in its function in autophagy.

To address this question, we generated four expression plasmids for *CORO1C*: the first one containing the full-length sequence (FL), the second containing a point mutation in the first actin-binding site (R28D), a third containing mutations in the second actin-binding site [(K418E, K419E/ K427E, K428E (2×KE)], and a fourth containing both R28D and 2KE mutations (R28D + 2KE) (Figure 6F ; Figure S6I). These four rescue constructs were packaged into lentivirus and used to transduce *CORO1C*-KO H4 cells. WB analysis confirmed the expression of the rescue constructs in *CORO1C*-KO H4 cells, with the expression levels exceeding those of endogenous CORO1C in WT H4 cells (**Figure S6J**). We observed that reintroducing either FL or R28D CORO1C into the *CORO1C*-KO H4 cells restored the normal morphology of autophagosomes and SQSTM1/p62 bodies, similar to those observed in WT cells (Figure 6G–I). In contrast, the introduction of the 2KE or R28D + 2KE constructs failed to rescue the abnormal phenotypes of autophagosomes and SQSTM1/p62 bodies (Figure 6G–I). These results emphasized the crucial role of the second actin-binding site of CORO1C in the formation of branched actin networks and the regulation of autophagy.

### The coro1c^−/−^ mice exhibit severe autophagic defects and spatial learning memory impairment

**Figure 7.**
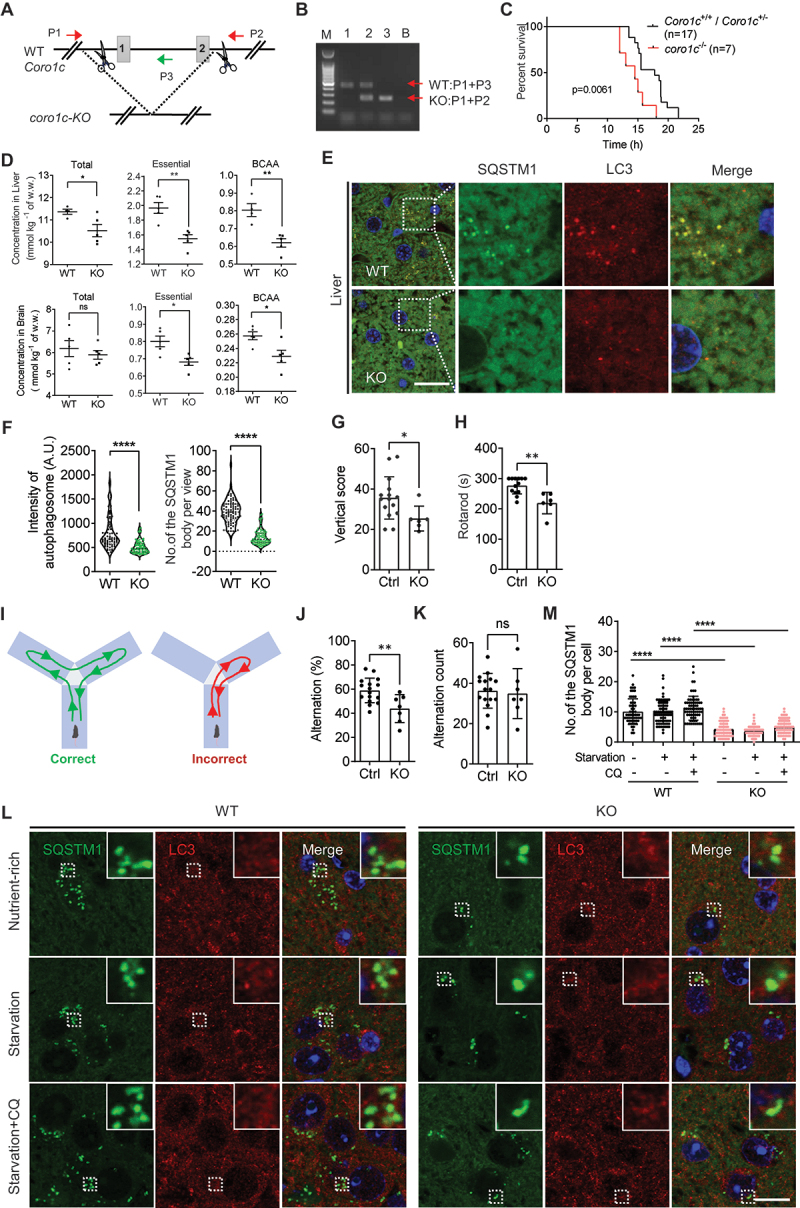
The *coro1c*^−/−^ mice exhibit autophagic defects and spatial learning memory impairment. (**A**) Strategy to create *coro1c^−/−^* mice by CRISPR-Cas9 system. (**B**) PCR analysis of genomic DNA extracted from *Coro1c^+/+^*, *Coro1c^+/-^* and *coro1c^−/−^* mice tail. B, blank control (ddH_2_O). (**C**) Survival profile of *Coro1c^+/+^*, *Coro1c^+/-^* (*n* = 17) and *coro1c^−/−^* (*n* = 7) mice. Neonates were obtained by caesarean delivery and monitored in a humidified chamber without milk feeding. (**D**) Tissues amino acid concentrations. Amino acid concentrations were measured at 10 h after the cesarean delivery under fasting conditions. “Total” indicates the sum of the asp, thr, ser, Asn, Glu, Gln, pro, gly, Ala, Val, cys, met, ile, leu, Tyr, phe, Lys, his and arg concentrations; “essential” indicates the sum of thr, Val, met, ile, leu, phe, Lys, his and arg concentrations; “BCAA” indicates the sum of the Val, ile and leu concentrations. Tissue amino acid concentrations are expressed as mmol kg^−1^ of wet weight. * *p* < 0.05, ** *p* < 0.01; ns, not significant. (**E**) Representative confocal images of liver in wt and *coro1c^−/−^* mice with LC3 (red) and SQSTM1/p62 (green) antibody. Nuclei are labeled with DAPI (blue). Scale bar: 20 μm. (**F**) Quantification of intensity of autophagosome and number of SQSTM1/p62 bodies in (E), *n* > 80 views were assessed from 3 mice in each group. (**G**) vertical score during the open field test (*n* = 6 in KO and *n* = 15 in Ctrl group). (**H**) Latency to fall off the rod during the rotarod test (*n* = 6 in KO and *n* = 13 in Ctrl group). (**I**) experimental scheme of the Y-maze. (**J, K**) Quantification of the ratio of spontaneous alternations (J) and the total number of alternations (K) in the Y-maze (*n* = 7 in KO and *n* = 16 in Ctrl group). (**L**) Representative confocal images of hippocampus in wt and *coro1c^−/−^* mice with LC3 (red) and SQSTM1/p62 (green) antibody. Nuclei are labeled with DAPI (blue). Scale bar: 10 μm. (**M**) Quantification the number of SQSTM1/p62 bodies in (L). *n* > 60 cells were assessed in each group. Data are presented as mean ± SEM. Unpaired t-test for d, F, H, I, j, L and M. * *p* < 0.05, ** *p* < 0.01, **** *p* < 0.0001; ns, not significant.

To evaluate whether CORO1C is critical for autophagy in vivo, the constitutive *coro1c* knockout (c*oro1c*^−/−^) mice were generated by using CRISPR-Cas9 system (Figure 7A,B; Figure S7A). We found that *coro1c*^−/−^ mice were born at the expected Mendelian frequency (**Figure S7B**). These mice appeared almost normal at birth (**Figure S7C**), and could survive into adulthood without gross phenotypic differences compared with their WT littermates (**Figure S7D**), except the mean body weight of 10 weeks *coro1c*^−/−^ male mice (23.72 ± 0.6145 g, *n* = 6) was significantly lower than that of WT and heterozygote littermates (26.55 ± 0.2488 g, *n* = 11; *p* = 0.0001). As autophagy is critical for survival during neonatal starvation [32], we assessed the survival time of neonates under non-suckling conditions after cesarean delivery. Our findings show that WT and heterozygous neonates died at 17.22 ± 0.64 h after birth, whereas *coro1c*^−/−^ neonates died at 14.32 ± 0.83 h (*p* = 0.0061; Figure 7C). Because the major role of autophagy is the degradation of proteins into amino acids [32], we measured the plasma and tissue amino-acid concentration under fasting conditions. Soon after the cesarean delivery, the concentration of amino acid in the plasma or tissues of *coro1c*^−/−^ neonates was not different from that of control littermates (**Figure S7E**). However, at 10 h starvation after the cesarean delivery, the amino acids concentration in the livers and brains of *coro1c*^−/−^ neonates was significantly lower than that of control neonates (Figure 7D); additionally, the amino-acid concentration showed a decreasing trend in the plasma of *coro1c*^−/−^ neonates (**Figure S7F**). Together, these results indicated that CORO1C is crucial for the cellular recycling of amino acids and for the survival of newborn mice under starvation conditions.

To investigate the role of CORO1C in the autophagic processes of adult mice, we assessed autophagic activity in the liver tissues of adult mice. IF staining revealed that the intensity of autophagosomes and the number of SQSTM1/p62 bodies was significantly decreased in the liver of *coro1c*^−/−^ mice (Figure 7E,F).

Recent studies have linked CORO1C to nervous system diseases, identifying it as a biomarkers for Alzheimer [33] and noting mutations in schizophrenia patients [34]. Interestingly, other work showed that the *coro1c*^−/−^ mice exhibited neurological and behavioral alterations, such as reduced hearing and hypoactivity [35]. Considering that a primary feature of neurological disorders is the impairment of learning and memory functions [36], we aimed to examine whether the neuro-related behaviors of *coro1c*^−/−^ mice were abnormal. In the open field test, while no significant differences were observed in total travel distance, velocity, or central zone crossings (**Figure S7G-I**), *coro1c*^−/−^ mice displayed a notable increase in vertical wall-rearing frequency (Figure 7G), indicating altered thigmotactic behavior. Motor coordination deficits were apparent in the rotarod assay, as *coro1c*^−/−^ mice exhibited a significantly reduced latency to fall compared to their littermate controls (Figure 7H). Cognitive assessment using the Y-maze paradigm (Figure 7I) revealed impaired spatial working memory in the mutant mice, evidenced by a significantly reduced spontaneous alternation percentage (Figure 7J), despite similar total arm entries between the groups (Figure 7K). In the novel arm experiment, *coro1c*^−/−^ mice spent significantly less time in the novel arm than WT and heterozygous mice (**Figure S7J**). These behavioral phenotyping results demonstrated that the absence of *coro1c* in mice results in spatial memory impairment, decreased exploration behavior and short-term memory defects.

To assess autophagic status in mouse neurons, we subjected mice to starvation or chloroquine (CQ) treatment and analyzed hippocampal sections immunostained for LC3 and SQSTM1/p62. WT neurons showed a substantial presence of normal autophagosomes and SQSTM1/p62 bodies under these conditions. In contrast, KO neurons exhibited a significant reduction in these structures under baseline conditions, starvation, or starvation combined with CQ treatment (Figure 7L,M). Furthermore, WB analysis revealed that starvation-induced increase in the LC3-II:LC3-I ratio and the decrease in SQSTM1/p62 levels were both blunted in the hippocampus of *coro1c*^−/−^ mice compared to WT control (**Figure S7K**). Collectively, these findings indicate that *coro1c*^−/−^ mice display pronounced autophagic defects alongside impaired spatial learning and memory, suggesting a potential functional link between disrupted autophagy and cognitive deficits.

## Discussion

**Figure 8.**
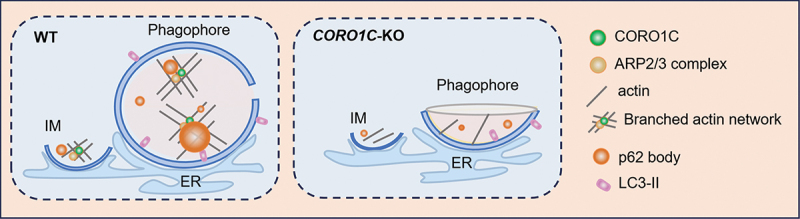
Schematic diagram of the role of CORO1C in autophagy.

Recently, the role of the actin cytoskeleton in autophagic processes illustrates the regulatory mechanism of autophagy from a new perspective [37,38]. In this study, we performed a genome-wide genetic screen and identified that CORO1C regulates the formation of SQSTM1/p62 bodies and autophagosomes by promoting the assembly of an ARP2/3-derived branched actin network within the autophagic structures (Figure 8). The ARP2/3 complex, composed of two actin-related proteins (ARP2 and ARP3) and five scaffolding subunits (ARPC1-5), is an evolutionarily conserved molecular machine that generates branched actin networks [27]. The ARP2/3 complex can be activated by nucleation promoting factors, while coronin is the first and most extensively studied ARP2/3 complex inhibitor [39]. In the coronin family, budding yeast has a single coronin (Crn1), whereas mammals possess seven coronins, categorized into three types based on sequence similarity [29].

To date, most data suggest that yeast coronin and type-1 mammalian coronins are branched network destabilizers or inhibitors of the ARP2/3 complex. For instance, the interaction between CORO1B and ARP2/3 has been shown to inhibit the ARP2/3 activity [40], CORO1B disassembles ARP2/3-containing actin filament branches by inducing ARP2/3 dissociation [41]. CORO1C has also been reported to confine actin structures to the base of the endosome by limiting ARP2/3 activity [42]. However, in our current study, we were initially surprised to find that the deficiency of CORO1C disrupts the formation of the branched actin network, while exogenous expression of CORO1C restores the formation of the branched actin network and rescues the defective autophagy phenotypes. If the only role of CORO1C is debranching, the formation of branched actin networks would not be affected. Thus, these results imply that CORO1C may not only act as an inhibitor of the ARP2/3 complex as demonstrated in previous studies but also play an unanticipated role in promoting the formation of the branched actin network during autophagy.

Interestingly, our results show that CORO1A and CORO1B did not regulate autophagy in the same manner as CORO1C. Notably, although CORO1A did not exert an impact on autophagy in the cell types examined in our present study, it can negatively regulate autophagy in immune-related cells. For example, CORO1A can obstruct the phagosome-lysosome fusion in macrophages [43], and inhibit autophagosome formation around phagosomes containing *Mycobacterium tuberculosis*, thereby promoting mycobacterial survival in macrophages [44]. Unlike that in immune-related cells, CORO1A is expressed at extremely low levels in other cell types, providing a reasonable explanation for its lack of effect on autophagy in our study. Both CORO1B and CORO1C are ubiquitously expressed at similar levels. However, the second actin-binding site in the “unique” region of CORO1C gives it a distinct regulatory role in autophagy.

A recent study revealed that the yeast Crn1 contains a second actin-binding site within its coiled-coil domain [45]. It has been reported that the interaction between the CC domain and actin effectively inhibits cofilin-mediated severing of newly assembled actin filaments (ATP/ADP+P(i)). In contrast, in the older regions of the actin filament, the Crn1 β-propeller domain works synergistically with cofilin to sever older filaments (ADP). This distinct effect on actin dynamics, depending on the nucleotide state of actin, requires two separate actin-binding domains in Crn1 [45]. Another study reported that at low concentrations, Crn1 does not inhibit but rather activates the ARP2/3 complex. Specifically, a central-acidic (CA) sequence, which binds to the ARP2/3 complex and triggers its conformational activation, has been identified within the “unique” region of Crn1. Point mutations within this CA sequence abolish the activation of the ARP2/3 complex by Crn1 *in vitro* [46]. However, this model cannot be directly applied to mammalian coronins given the absence of a CA sequence in their “unique” regions [39]. Collectively, these findings imply that Crn1 exerts a dual influence on both actin filaments and the ARP2/3 complex, and this bimodal mechanism appears to be indispensable for the normal functioning of Crn1.

The results obtained in two recent studies [45,46], combined with our observations described above, (showing that coronin has two separate actin-binding domains with markedly different effects on cofilin and the ARP2/3 complex), led us to investigate whether the effects of CORO1C on the activity of the ARP2/3 complex may differ under normal or autophagic conditions. CORO1C has the highest homology with Crn1 among type 1 coronins (**Figure S8A**); similar to Crn1, CORO1C contains two actin-binding sites, implying functional similarity. Significantly, across species from zebrafish to mice and humans, the CORO1C protein consistently consists of 474 amino acids, while the amino acid count in CORO1A and CORO1B varies across different species (**Figure S8B, C**). The following question arises: How does CORO1C regulate autophagy or other cellular processes via the bimodal mechanism of branched actin networks? We hypothesized that mechanisms linked to the nucleotide state [45] or alternation in concentrations [46] are not mutually exclusive, and may all contribute to the regulation of branched actin network formation or debranching at the appropriate time and location.

The formation of the autophagosome membrane [8] and SQSTM1/p62 bodies [9] is regulated by the branched actin network, and the appropriate size of these p62 condensates is crucial for their degradation via autophagy [9]. However, there must be mechanisms in place to limit the size of SQSTM1/p62 bodies to ensure they can adapt to the appropriate size of the autophagosome. CORO1C has been shown to restrict endosomal branched actin, thereby facilitating the organization of ER contact sites and endosome fission [42,47]. In the absence of CORO1C and its paralogs, actin proliferates along the length of the endosome bud, which hinders the proper recruitment of the ER membrane contact sites and obstructs endosome fission. CTTN-mediated stabilization of the ARP2/3 complex enhances the stability of the endosome bud, allowing slow-diffusing cargos within it [48]. We postulate that similar to its role in endosome fission, CORO1C may functionally interact with the ARP2/3 complex to promote the initial formation of branched actin networks, facilitating the formation of autophagosomes and SQSTM1/p62 bodies. CORO1C may then restrict the activity of the ARP2/3 complex to ensure that the phagophore is constrained to the appropriate size and shape, encapsulating substrates, such as the SQSTM1/p62 bodies, to form a complete autophagosome. Indeed, CORO1C may regulate the formation of autophagic structures and the proper termination of elongation, owing to its dual-function with respect to the ARP2/3-driven branched actin networks. We should note that our speculation needs to be supported by more biochemical experiments, such as the isolation of autophagosomes and western blotting of CORO1C, actin, ARPC2, SQSTM1/p62, and LC3, which will provide further direct evidence in support of our conclusions. In addition, CORO1C has been shown to activate RAC1 (Rac family small GTPase 1) [49], a Rho GTPase whose family members can participate in autophagosome formation through actin remodeling [50,51]. Therefore, CORO1C may also regulate autophagosome formation by regulating RAC1 (or other Rho GTPases), and it is one of the focuses of our future research to verify this conjecture by measuring GTPases activity.

Studies have demonstrated that *CORO1C* is widely expressed in the central nervous system from embryonic stages to adulthood, participating in the regulation of neuronal morphogenesis and migration [52]. Its functional deficiency may lead to corpus callosum defects in mice [53]. In disease associations, CORO1C has been identified by machine learning models as a potential biomarker for Alzheimer disease [33] and directly interacts with LRRK2, a key pathogenic factor in Parkinson disease, influencing disease penetrance [54]. Notably, CORO1C has demonstrated therapeutic potential in spinal muscular atrophy (SMA) models. In zebrafish with *SMN* (survival motor neuron) gene deficiency, *CORO1C* overexpression rescues the axonal truncation and branching phenotype caused by SMN loss through elevating F-actin levels [55]. Recent studies have revealed the involvement of CORO1C in mitochondrial quality control systems: mass spectrometry analysis confirm its direct interactions with PRKN/Parkin (parkin RBR E3 ubiquitin protein ligase), a core regulator of mitophagy [56–58], the mitophagy receptor protein CALCOCO2/NDP52 (calcium binding and coiled-coil domain 2) [59], and mitochondrial complex subunits (NDUFS1, NDUFS2, NDUFS3, etc.) [60]. Research has shown that *coro1c* knockout in mouse embryonic fibroblast cells results in abnormal subcellular localization and functional impairments of mitochondria [61]. Given that dysregulated mitophagy represents a critical pathological mechanism in neurodegenerative diseases such as Alzheimer and Parkinson disease [62], and considering CORO1C’s dual roles in regulating neuronal development and mitochondrial homeostasis, this protein may contribute to the pathogenesis of neurological disorders by mediating mitophagy pathways.

The absence of a severe or lethal phenotype in the *coro1c*-deficient mice may be attributed to functional redundancy within the Coronin family and the plasticity of the actin regulatory network. Given the particularly high expression of CORO1C in neurons [25,34], its knockout in mice may lead to a relatively mild overall phenotype. In other affected tissues, ubiquitously expressed homologs may compensate for the loss by sustaining ARP2/3-dependent actin nucleation. Additionally, other critical actin regulators, such as Cofilin and Profilin, may undergo adaptive changes in their activity or expression levels to preserve essential actin dynamics and autophagy. This compensatory network likely mitigates the impact of *CORO1C* loss, particularly in tissues where its homologs are present.

Overall, our study demonstrates the unexpected role of CORO1C in promoting the formation of branched actin networks in conjunction with the ARP2/3, which is essential for the formation of phagophore, autophagosomes, and SQSTM1/p62 bodies. However, whether CORO1C directly interacts with known core autophagy proteins remains undetermined. Further research may uncover the detailed mechanisms underlying the role of CORO1C in autophagy regulation and its potential implications in disease treatment.

## Materials and methods

### Mice

The *coro1c*-KO mice (Strain NO. T011224) were purchased from GemPharmatech (Nanjing, China). The mice were kept with an alternating 12-h light/dark cycle and provided with standard chow and water ad libitum. The Institutional Animal Care and Use Committee at Peking Union Medical College & Chinese Academy of Medical Sciences approved all mice procedures (ID: ACUC-A01-2025–021). All mice were fed in specific pathogen-free facilities. For starvation, 10 weeks old male mice were deprived of food for 24 h. These mice had free access to drinking water. For chloroquine (Sigma-Aldrich, C6628) treatment, 60 μg/g of chloroquine was injected intraperitoneally, and the control group was injected with a saline solution.

### Cell cultures and drugs treatment

Mouse haESC line OdG, derived from AGH-OG-3 (OG3) by deletion of the EGFP cassette [16], were cultured in M2iL mediums, which containing knockout DMEM (Gibco, 10,829–018), 15% fetal bovine serum (HyClone, SH30396.03), 1% GlutaMAX (Gibco, 35,050–061), 1% non-essential amino acids (Gibco, 11,140,050), 100 μM β-mercaptoethanol (Gibco, 21,985–023), 100 U Leukemia inhibitory factor (Merck Millipore, ESG1107), 3 μM CHIR99021 (Selleck Chemicals LLC, S2924) and 1 μM PD0325901 (Selleck Chemicals LLC, S1036). H4 and 293 FT cell lines were obtained from the Cell Resource Center, Peking Union Medical College (the headquarter of National Science & Technology Infrastructure-National BioMedical Cell-Line Resource, NSTI-BMCR). H4 and 293 FT cells were maintained at 37°C in 5% CO_2_, in Dulbecco’s modified Eagle medium (Gibco, C11995500) supplemented with 10% fetal calf serum (VivaCell, C04001), and 100 μM β-mercaptoethanol (Gibco, 21,985–023).

For starvation treatment, cells were washed twice with DPBS (Gibco, C14190500BT) and cultured in E15 (For haGRL and AB2.2 ESCs) or E10 (For H4, 293 FT cells) medium, which containing Earle’s balanced salt solution (Gibco, 14,155–063) and 15% or 10% fetal bovine serum (VivaCell, C04001). For bafilomycin A_1_ (Selleck Chemicals LLC, S1413) treatment, cells were cultured with 100 nM bafilomycin A_1_ for 2 h. For CK666 (Sigma-Aldrich, SML0006) treatment, cells were cultured with 100 μM CK666 for 4 h.

### Plasmids

pX330 plasmids was provided by F. Zhang through Addgene (42230). pMRX-IP-GFP-LC3-RFP-LC3ΔG (Addgene, 84,572) was a gift from Noboru Mizushima. cDNA encoding human *CORO1C* was inserted into pCDH-CMV-MCS-EF1α-Puro (Youbio, VT1480). cDNA encoding human CTTN was inserted into pcDNA3.1-mCherry-C2. cDNA encoding human ARPC2 was inserted into pmCherry-N1. pEGFP-actin (VT1128) was purchased from Youbio. PBDGTV (Addgene, 100,859; deposited by Yue Huang) and pCMV-hyPBase were generated previously [18]. Truncated constructs were prepared by PCR mediated site-directed mutagenesis.

### Preparation of lentivirus and cell infection

For preparation of lentivirus, 293 FT cells were transfected with a lentivirus vector together with helper plasmid (psPAX2 and pMD2.G) using lipofectamine 3000 (Thermo Fisher Scientific, L3000001). After 48 ~ 72 h culture, the supernatant was passed through a 0.45 μm filter unit (Merck Millipore, SLHVR33RB) and was concentrated with PEG8000 (AMRESCO, 0159). Then cells were infected with concentrated lentivirus, and the stable cell lines were selected using puromycin (Sigma-Aldrich, P8833).

### FACS for haploid ESCs

For sorting cells state in haploid, haESC OdG were trypsinized and stained with Hoechst33342 (5 μg/ml) (Sigma-Aldrich, B2261) for 20 ~ 30 min at 37°C. After filtered by a 40-μm cell strainer, the haploid in 1n peak were purified with a Beckman Moflo-XDP cell sorter. And diploid (2 n) ES cells were used as a control.

### Autophagic flux determination by FACS

Cells stably expressing GFP-LC3-RFP-LC3ΔG were treated with 0.05% trypsin-EDTA for 2 ~ 3 min and collected in ice-cold PBS (Servicebio, G4202). After washing, cells were analyzed on a Beckman Moflo-XDP cell sorter equipped with 488-nm and 561-nm lasers. To display autophagy induction, the fluorescence level in full medium or DMSO control condition was set to 100% for every cell line and the remaining fluorescence signal after autophagy induction was calculated accordingly.

### Generation of the genome-wide mutant library

For mutagenesis, haESCs with a high 1n peak were purified by FACS and were further cultured for 5 ~ 6 days. Ten million cells were transfected by electroporation (Bio-Rad Gene Pulser, 230 V, 500 μF, 800 Ω) with 1 μg PBDGTV transposon donor plasmid and 10 μg pCMV-hyPBase transposase expression plasmid. After electroporation, the cells were plated onto 90-mm feeder plates. Puromycin selection (1 μg/ml) was initiated 24 h later and continued for 5 days until individual ES cell clones were visible. The puromycin-resistant clones within each plate were pooled and expanded for 4 days to generate one library called the Haploid-PBDGTV Mutant Library (HML).

### Generation of the Coro1c-KO cells

The sgRNAs designed from E-CRISP (http://www.e-crisp.org/E-CRISP/) for each gene were produced with p×330 plasmids (Addgene, 42,230; deposited by Feng Zhang) (A “G” was added to the 5’ end of the sgRNA when the first base was not “G,” in order to ensure optimal expression from the *U6* promoter). *Coro1c*-sgRNA1 and *Coro1c*-sgRNA2 plasmids (1.5 μg each) and puromycin-selection plasmid PB-puro plasmid (0.5 μg) were co-electroporated (Invitrogen, Neon Transfection System) into cells and cultured for 24 h. After 48 h of puromycin-selection (1 μg/ml), cells were trypsinized and transferred to 10 cm-plates at a low density and cell colonies were picked at day 7. Genomic DNA was extracted for PCR and further sequenced to verify the deletions in the *Coro1c* gene. The oligos and primers are listed in Table S2.

### Splinkerette PCR

The Splinkerette PCR method to identify the transposon insertion sites was the same as that as previously described [63]. In brief, genomic DNA isolated from haESC colonies was extracted, digested and ligated with the corresponding Splinkerette adaptors HMSp-Sau3AI (generated by annealing Splinkerette oligos HMSpBb-Sau3AI with HMSpAa), followed by two rounds of nested PCR. And the final PCR products were sequenced and aligned to the mouse genome to identify the sites of transposon. All the primers used in Sp-PCR are listed in Table S2.

### Splinkerette-PCR combined with NGS and bioinformatics analysis

The methods of DNA library preparation and bioinformatics analysis was the same as that previously described [18]. Briefly, ten micrograms of genomic DNA from each mixed library were sheared by focused sonication with a fragment size of approximately 200 to 400 bp using the Covaris sonication system (Covaris S220). The fragmented DNA was purified using AMPure XP beads (Beckman Coulter, B23318) following the manufacturer’s instructions. We used the NEBNext Ultra II DNA Library Prep Kit for Illumina (New England Biolabs, E7645) to repair and added 3’dA overhangs of these fragments, then ligate with Splinkerette T-overhang linker (generated by annealing Splinkerette oligos SplkTp with SplkTm). Then, the junction fragments for the 3” and 5‘ ends of the *PiggyBac* transposon (PB3’ ITR and PB5” ITR) were amplified in two consecutive Splinkerette PCR rounds to generate PB3 and PB5 libraries for each library, and a size selection of 300–500 bp fragmented DNA was performed using AMPure XP beads. These libraries were sequenced on a single lane of an Illumina HiSeq2500 device at BGI with pair-end reads of 2 × 125 bases read lengths following the manufacturer’s recommended conditions. The PhiX DNA was added to libraries to increase complexity for Illumina sequencing.

For bioinformatics analysis, the read pairs were trimmed of adapters and PB tags (TATCTTTCTAGGGTTAA) before mapping to the genome using a FASTX-toolkit (http://hannonlab.cshl.edu/fastx_toolkit/commandline.html) with the default parameter. The trimmed paired end reads were aligned to the mouse reference genome (mm10) with Bowtie 2 software (http://bowtie-bio.sourceforge.net/bowtie2). The analyzed data could be seen in Table S1.

### Immunoblotting

Whole-cell lysates were separated by SDS-polyacrylamide gel electrophoresis (SDS-PAGE), and electroblotted to polyvinylidene difluoride membranes (Bio-Rad, 162,077). The membranes were blocked with 5% skim milk for 1 h at room temperature. The membranes were incubated with the primary antibody overnight at 4°C, followed by treatment with HRP-conjugated secondary antibody for 1 h at room temperature. Primary antibodies used were as follows: Rabbit anti-ATG5 (Novus Biologicals, NB110-53818), rabbit anti-LC3B (Cell Signaling Technology, 3868S), rabbit anti-SQSTM1/p62 (Abclonal, A11483), rabbit anti-SQSTM1/p62 (Proteintech, 31,403–1-AP; Selleck, D16H4 for brain tissues), rabbit anti-CORO1C (Proteintech, 14,749–1-AP), rabbit anti-ARPC2 (Proteintech, 15,058–1-AP), mouse anti-GAPDH (Santa Cruz Biotechnology, sc-47724) and mouse anti-ACTB/β-actin (Cell Signaling Technology, 3700). Secondary antibody used were as follows: goat anti-rabbit IgG (ZSGB-BIO, ZB-2301) and goat anti-mouse IgG (ZSGB-BIO, ZB-2305).

### Immunofluorescence (IF) staining

Cells cultured in Chamber Slides were fixed by 4% paraformaldehyde (Servicebio, G1101) at room temperature for 15 min. After washed by PBS for 3 times, cells penetrated with 0.5% Triton X-100 (Sigma-Aldrich, 9036–19-5) at room temperature for 10 min and then incubated in 3% BSA (Sigma-Aldrich, B2064) at room temperature for 1 h. After washed by PBS for 3 times, cells were then incubated overnight at 4°C with primary antibodies. After washed by PBS for 3 times, cells were incubated with the corresponding secondary antibodies for 1 h at room temperature.

Primary antibodies used were as follows: mouse anti-SQSTM1/p62 (Santa Cruz Biotechnology, sc-28359), anti-LC3B Rabbit mAb (Abclonal, A19665), rabbit anti-CORO1C (Proteintech,14749–1-AP), rabbit anti-RB1CC1 (Proteintech, 17,250–1-AP), rabbit anti-STX17 (Proteintech, 17,815–1-AP), mouse anti-CORO1C (Santa Cruz Biotechnology, sc-376919), rabbit anti-ARPC2 (Proteintech, 15,058–1-AP). All the secondary antibodies were purchased from Invitrogen (Alexa fluor 647 Donkey anti-Rabbit IgG, A-31573; Alexa fluor 546 Goat anti-Mouse IgG, A-11030; Alexa fluor 488 Goat anti-Rabbit IgG, A-11008,). DNA was labeled with DAPI (Invitrogen, S36939). Confocal fluorescence microscopy or STED was performed on Leica TSC SP8 (63× oil lens). super-resolution fluorescence microscopy was performed on Abberior Stedycon.

Dissected organs were fixed in 4% paraformaldehyde prior to optimal cutting temperature embedding. Paraffin sections were prepared using standard procedures. Sections were rehydrated and treated with 3% hydrogen peroxide to suppress the endogenous peroxidase activity. Antigen retrieval was achieved by boiling at 121°C for 15 min in 10 mM citrate buffer, pH 6.0 followed by gradual cooling to room temperature. After permeabilized with 0.1% Triton X-100 (Sigma-Aldrich, X100), the sections were incubated in a blocking buffer (ZSGB-BIO, ZLI-9022), and then probed with the primary antibodies diluted in the blocking buffer overnight at 4°C. The sections were washed in PBS, followed by the addition of the appropriate secondary antibody diluted in blocking buffer for 30 min. Coverslips were washed again with PBS and mounted with mounting medium containing DAPI (Invitrogen, S36939). Primary antibodies were used at the following concentrations: rabbit anti-LC3B (Cell Signaling Technology, 3868S), and mouse anti-SQSTM1/p62 (Santa Cruz Biotechnology, sc-28359).

### Electron microscopy analysis

Cell samples were pre-fixed for more than 4 h in 2.5% glutaraldehyde in 0.1 M sodium phosphate buffer, pH 7.4, and then rinsed with the same buffer, subsequently fixed in 1% osmium tetroxide in ddH_2_O. Samples were then dehydrated in a graded series of ethanol solutions (50, 70, 80, 90, 100%) and embedded in SPI-Pon 812 Epoxy Resin Monomer (SPI Supplies, 02659-AB). Seventy-nanometer sections were cut parallel to the substrate and post-stained with 2% uranyl acetate and Reynolds lead citrate. Sections were observed with a transmission electron microscope (TEM-1400plus, JEOL, Japan) at 80 kV. Images were acquired using 832 CCD camera (Gatan, Pleasanton, CA).

### Cesarean delivery and measurement of amino acids

Newborns were delivered by cesarean section at 19.0 d postcoitus and placed in a humidified, thermostat-controlled chamber (30°C). Plasma was stored in EP tube treated with heparin sodium. Amino acids in the supernatant from plasma samples or tissues were measured by HPLC-MS assay.

### Rotarod test

The rotarod test was used to assess motor coordination. Before testing, the mice were trained three times from 5 to 40 rpm in 300 s for 2 consecutive days. During the testing, the rotarod speed was increased from 5 to 40 rpm within 300 s. The latency to fall off the rod was recorded automatically for each mouse, and the average latency of three trials, with an interval of 30 min, was calculated for further analysis.

### Open field test

Evaluate sports activities through open field tests. The instrument consists of a white chamber (40 × 40 × 40 cm). Put each mouse in the center and let it explore the arena for 5 min. The motion trajectory is automatically recorded by camera and analyzed by Ethovision-XT software. Measure the total distance traveled and the number of shuttles in the central area to evaluate sports activities. After each test, the arena was cleaned with 70% ethanol.

### Y maze test

The Y-maze was used to study cognitive ability and spatial working memory. This device consists of three radiating arms that were arranged at equal angles (120°).

1. Spontaneous Alternation: At the beginning of the trial, the experimental mice were placed in one of the fixed arms and allowed to move freely in the maze for 10 min. Efficient behavior was defined as continuous access to all three arms in overlapping sets of three choices.
2. Novel arm test: The Y-maze test consisted of 2 trials. The first trial (training) had 5 min duration and allowed the mouse to explore only two arms (start arm and other arm) of the maze, with the third arm (novel arm) blocked. After 4 h, the second trial was conducted, during which all three arms were accessible and novelty vs. familiarity was analyzed by comparing behavior in all three arms. For the second trial, the mouse was placed back in the maze in the same starting arm, with free access to all three arms for 5 min. By using a ceiling-mounted CCD camera, all trials were recorded on a VCR. Video recordings were later analyzed and the duration, distance and number of visits in each arm were analyzed.

### Statistical analyses

To present quantitative data, Microsoft Excel and GraphPad Prism 6 were utilized and figures are structured in Microsoft Power Point or Adobe Illustrator. Unless otherwise stated in the figure legends, *p*-values for quantification of fluorescence microscopy and TEM data were generated by two-tailed Student’s t test. WB data displayed were representative experiments, and the number of independent experiments from biological replicates conducted is indicated in the legend. For all statistical analysis, a value of *p* < 0.05 was considered to be statistically significant and *p* > 0.05 was considered non-significant (ns).

## Supplementary Material

COR1C_in_Autophagy_Supplementary_Figures_Tables_5th_Revision_Final R6.docx

## Data Availability

All data are available in the manuscript and the supplementary materials are available upon request from the authors.
