# Supplementary material for: CORO1C (coronin 1C) promotes autophagosome formation by coordinating branched actin network dynamics
Source: Autophagy. 2026 Apr 20;22(7):1601–18. doi: 10.1080/15548627.2026.2658234 (PMC13285634; doi:10.1080/15548627.2026.2658234)
Supplement: COR1C_in_Autophagy_Supplementary_Figures_Tables_5th_Revision_Final R6.docx [file KAUP_A_2658234_SM2145.docx]

**Supplementary Figures and Tables**

**Table S1.** The list of transposon insertion sites in HML.

| **gene_id** | **Insertion site** | **region** | **log2FoldChange** | **pvalue** | **padj** | **Type** |
| --- | --- | --- | --- | --- | --- | --- |
| *Crocc* | chr4:141044054 | Intron | 13.26650822 | 4.62E-10 | 7.76E-10 | up |
| *Osbpl11* | chr16:33190427 | Intron | 12.6265429 | 7.03E-103 | 4.89E-102 | up |
| *Nebl* | chr2:17620588 | Intron | 12.39318268 | 3.01E-09 | 4.91E-09 | up |
| *Sult2b1* | chr7:45759193 | Intron | 11.60376127 | 1.49E-145 | 1.43E-144 | up |
| *Kcnk12* | chr17:87756901 | Intron | 11.53296884 | 1.47E-95 | 9.85E-95 | up |
| *Atg14* | chr14:47551787 | Intron | 11.10819408 | 2.96E-200 | 4.09E-199 | up |
| *Gm17655* | chr5:110068744 | Intron | 10.97287412 | 2.18E-169 | 2.52E-168 | up |
| *Sgms1* | chr19:32319316 | Intron | 10.92200868 | 1.18E-300 | 2.93E-299 | up |
| *Gm44613* | chr7:18079537 | Intron | 10.75832721 | 1.03E-112 | 7.79E-112 | up |
| *Grrp1* | chr4:134251110 | exon | 10.61339403 | 0.001683466 | 0.002051902 | up |
| *Wipi2* | chr5:142632990 | Intron | 10.49014041 | 3.56E-212 | 5.46E-211 | up |
| *Synpr* | chr14:13300122 | Intron | 10.31445746 | 2.56E-136 | 2.29E-135 | up |
| *Il7* | chr3:7578820 | Intron | 10.30681212 | 1.50E-245 | 2.79E-244 | up |
| *1700102P08Rik* | chr9:108393680 | Intron | 10.22726027 | 1.71E-49 | 7.50E-49 | up |
| *Mthfd1l* | chr10:4044033 | Intron | 9.984162652 | 3.89E-126 | 3.27E-125 | up |
| *Ccdc107* | chr4:43495467 | exon | 9.982701725 | 1.10E-100 | 7.58E-100 | up |
| *Tm9sf3* | chr19:41258973 | Intron | 9.889143702 | 1.18E-254 | 2.30E-253 | up |
| *Ulk1* | chr5:110797306 | Intron | 9.859604259 | 3.19E-09 | 5.20E-09 | up |
| *Atg7* | chr6:114662791 | Intron | 9.688978864 | 2.46E-256 | 4.88E-255 | up |
| *Skp1a* | chr11:52236814 | Intron | 9.642667319 | 3.86E-164 | 4.31E-163 | up |
| *Spon2* | chr5:33199019 | Intron | 9.624207798 | 1.81E-192 | 2.41E-191 | up |
| *Mst1r* | chr9:107916443 | exon | 9.604185311 | 7.48E-74 | 4.18E-73 | up |
| *Qtrt2* | chr16:43884252 | Intron | 9.550376404 | 3.52E-80 | 2.09E-79 | up |
| *Gm15802* | chr16:38833008 | Intron | 9.545519097 | 9.73E-14 | 1.89E-13 | up |
| *Rnf39* | chr17:36946727 | Intron | 9.525235227 | 4.59E-307 | 1.15E-305 | up |
| *Mlip* | chr9:77302261 | Intron | 9.455256813 | 5.16E-78 | 3.01E-77 | up |
| *Stxbp4* | chr11:90584695 | Intron | 9.388317816 | 3.12E-251 | 6.00E-250 | up |
| *Rexo5* | chr7:119822389 | Intron | 9.258331279 | 5.28E-55 | 2.41E-54 | up |
| *Gm15326* | chr13:111869063 | Intron | 9.09155542 | 2.99E-09 | 4.89E-09 | up |
| *Ppp4r1* | chr17:65776793 | intergenic(10kb) | 9.047716436 | 2.29E-09 | 3.76E-09 | up |
| *Snx1* | chr9:66124382 | Intron | 8.984172551 | 2.02E-257 | 4.05E-256 | up |
| *Atg5* | chr10:44329515 | Intron | 8.950311638 | 1.19E-285 | 2.80E-284 | up |
| *Chchd3* | chr6:32910581 | Intron | 8.846302587 | 1.77E-227 | 3.10E-226 | up |
| *Gm829* | chr4:45687776 | Intron | 8.790457031 | 2.52E-09 | 4.11E-09 | up |
| *Rtn4rl1* | chr11:75203350 | Intron | 8.774132222 | 0.001606373 | 0.001959255 | up |
| *Vps26a* | chr10:62481522 | Intron | 8.644024167 | 1.39E-105 | 9.91E-105 | up |
| *Dmgdh* | chr13:93731370 | Intron | 8.63371353 | 1.68E-122 | 1.37E-121 | up |
| *Ptprb* | chr10:116305706 | Intron | 8.627114033 | 5.98E-159 | 6.41E-158 | up |
| *Fbrsl1* | chr5:110437352 | Intron | 8.569289066 | 7.34E-47 | 3.14E-46 | up |
| *Kank2* | chr9:21783380 | Intron | 8.422452454 | 3.12E-251 | 6.00E-250 | up |
| *Stard5* | chr7:83632800 | Intron | 8.387315787 | 2.44E-258 | 4.97E-257 | up |
| *Sntg1* | chr1:8486033 | Intron | 8.362961813 | 1.75E-208 | 2.60E-207 | up |
| *Fgd4* | chr16:16427161 | Intron | 8.329341255 | 8.64E-05 | 0.00011418 | up |
| *Plekha1* | chr7:130877001 | Intron | 8.318157401 | 1.95E-09 | 3.20E-09 | up |
| *Rcor3* | chr1:192101419 | three_prime_utr | 8.315118392 | 2.44E-258 | 4.97E-257 | up |
| *4930503L19Rik* | chr18:70458321 | Intron | 8.266186497 | 1.56E-80 | 9.30E-80 | up |
| *Lamb3* | chr1:193302044 | five_prime_utr | 8.263207382 | 4.92E-157 | 5.18E-156 | up |
| *4930470P17Rik* | chr2:170598171 | Intron | 8.203862981 | 2.62E-255 | 5.15E-254 | up |
| *Cped1* | chr6:21986326 | five_prime_utr | 8.15793673 | 0.001551783 | 0.00189395 | up |
| *Atg9b* | chr5:24387929 | Intron | 8.136522443 | 5.38E-220 | 8.86E-219 | up |
| *Cntn4* | chr6:105975624 | Intron | 8.07292519 | 1.76E-270 | 3.74E-269 | up |
| *Glo1* | chr17:30611810 | Intron | 8.025377643 | 8.12E-210 | 1.24E-208 | up |
| *Kcnip2* | chr19:45799786 | Intron | 8.008199385 | 2.13E-206 | 3.01E-205 | up |
| *2810403A07Rik* | chr3:88686871 | exon | 7.95252422 | 8.59E-05 | 0.000113698 | up |
| *Naa38* | chr11:69395667 | five_prime_utr | 7.918603162 | 4.72E-35 | 1.70E-34 | up |
| *Katnal1* | chr5:148928601 | Intron | 7.916643425 | 9.81E-35 | 3.52E-34 | up |
| *Pip5k1a* | chr3:95084912 | Intron | 7.901725396 | 5.64E-287 | 1.36E-285 | up |
| *Gss* | chr2:155588963 | Intron | 7.900822339 | 1.16E-18 | 2.65E-18 | up |
| *Gm29514* | chr1:146427246 | Intron | 7.899011004 | 2.25E-48 | 9.73E-48 | up |
| *Ccnj* | chr19:40842156 | Intron | 7.83542858 | 4.36E-188 | 5.76E-187 | up |
| *Vax2* | chr6:83717908 | Intron | 7.822559328 | 3.11E-172 | 3.66E-171 | up |
| *Clpx* | chr9:65313633 | Intron | 7.78451236 | 2.91E-138 | 2.64E-137 | up |
| *Egfem1* | chr3:29146992 | Intron | 7.773034513 | 2.11E-35 | 7.67E-35 | up |
| *Coro1c* | chr5:113878098 | Intron | 7.763590381 | 2.53E-35 | 9.16E-35 | up |
| *Ctnna3* | chr10:63465010 | Intron | 7.760199465 | 5.64E-287 | 1.36E-285 | up |
| *Kcmf1* | chr6:72862418 | Intron | 7.748807411 | 4.32E-171 | 5.04E-170 | up |
| *Ephb1* | chr9:102206307 | Intron | 7.705429633 | 7.53E-17 | 1.62E-16 | up |
| *Atg3* | chr16:45159441 | Intron | 7.657031421 | 2.36E-67 | 1.24E-66 | up |
| *Scin* | chr12:40104025 | Intron | 7.650636596 | 9.71E-05 | 0.000128017 | up |
| *Htatip2* | chr7:49762953 | Intron | 7.640060426 | 2.19E-207 | 3.15E-206 | up |
| *Rab30* | chr7:92768267 | Intron | 7.638923821 | 2.82E-49 | 1.23E-48 | up |
| *Pde7a* | chr3:19294815 | Intron | 7.615313047 | 2.50E-84 | 1.54E-83 | up |
| *Adgrb3* | chr1:25075099 | Intron | 7.566092094 | 9.00E-05 | 0.000118911 | up |
| *Gm20517* | chr17:47647056 | Intron | 7.533408404 | 1.25E-85 | 7.80E-85 | up |
| *Rb1cc1* | chr1:6244919 | CDS | 7.512640745 | 9.99E-05 | 0.000131548 | up |
| *Gm29282* | chr1:155056545 | Intron | 7.500763594 | 1.17E-18 | 2.67E-18 | up |
| *Txndc17* | chr11:72208152 | Intron | 7.450520155 | 2.25E-48 | 9.73E-48 | up |
| *Col19a1* | chr1:24579277 | Intron | 7.431655591 | 2.96E-200 | 4.09E-199 | up |
| *4930519F16Rik* | chrX:103243574 | Intron | 7.402533475 | 1.17E-46 | 4.98E-46 | up |
| *Cmtm4* | chr8:104393070 | Intron | 7.329088516 | 1.92E-43 | 7.92E-43 | up |
| *Tbce* | chr13:14034624 | Intron | 7.320150077 | 1.62E-48 | 7.03E-48 | up |
| *Gk5* | chr9:96120239 | Intron | 7.312868635 | 3.05E-69 | 1.62E-68 | up |
| *Gm11464* | chr2:165987119 | Intron | 7.306884088 | 3.12E-227 | 5.43E-226 | up |
| *1700028P15Rik* | chr2:171962561 | Intron | 7.283495979 | 1.80E-241 | 3.32E-240 | up |
| *Ndufaf5* | chr2:140179205 | Intron | 7.194143899 | 1.40E-67 | 7.40E-67 | up |
| *AC159106.2* | chr14:20221300 | exon | 7.185059295 | 3.13E-45 | 1.33E-44 | up |
| *Ercc4* | chr16:13145340 | Intron | 7.158193772 | 3.15E-45 | 1.33E-44 | up |
| *Rbms2* | chr10:128142563 | Intron | 7.121694346 | 3.19E-09 | 5.20E-09 | up |
| *Atg16l1* | chr1:87756596 | Intron | 7.117287621 | 7.36E-68 | 3.91E-67 | up |
| *Fam110b* | chr4:5693954 | Intron | 7.095840287 | 5.62E-46 | 2.39E-45 | up |
| *Crim1* | chr17:78267653 | Intron | 7.094557143 | 4.95E-164 | 5.50E-163 | up |
| *Rcc2* | chr4:140700776 | Intron | 7.088115431 | 2.02E-257 | 4.05E-256 | up |
| *Ephb2* | chr4:136759627 | Intron | 7.055664437 | 0.001527863 | 0.001867273 | up |
| *Grm4* | chr17:27422318 | intergenic(10kb) | 7.052915572 | 8.34E-76 | 4.77E-75 | up |
| *Adnp* | chr2:168192766 | Intron | 7.045147167 | 2.35E-240 | 4.30E-239 | up |
| *Tead1* | chr7:112770795 | Intron | 7.03854662 | 1.66E-267 | 3.42E-266 | up |
| *Klf3* | chr5:64822989 | Intron | 7.036117091 | 4.10E-48 | 1.77E-47 | up |
| *Dnmt3l* | chr10:78043970 | Intron | 7.029854981 | 8.43E-05 | 0.000111575 | up |
| *Cfdp1* | chr8:111770115 | Intron | 6.994088701 | 5.64E-46 | 2.39E-45 | up |
| *Slc25a13* | chr6:6114190 | Intron | 6.935909411 | 1.19E-285 | 2.80E-284 | up |
| *Gm43469* | chr3:151092637 | exon | 6.927375897 | 4.82E-273 | 1.06E-271 | up |
| *Fermt2* | chr14:45484310 | Intron | 6.91380726 | 1.61E-250 | 3.07E-249 | up |
| *Casd1* | chr6:4639675 | Intron | 6.894285761 | 4.09E-57 | 1.92E-56 | up |
| *Gm15564* | chr16:35969262 | Intron | 6.8745901 | 8.05E-208 | 1.17E-206 | up |
| *Cep95* | chr11:106807369 | Intron | 6.870774542 | 4.10E-48 | 1.77E-47 | up |
| *Gm12524* | chr3:108175868 | Intron | 6.860949636 | 2.56E-35 | 9.25E-35 | up |
| *Emp1* | chr6:135363764 | Intron | 6.850901939 | 2.60E-116 | 2.05E-115 | up |
| *Sipa1l2* | chr8:125456784 | Intron | 6.823820763 | 4.21E-236 | 7.55E-235 | up |
| *Slc3a1* | chr17:85061566 | Intron | 6.774075784 | 5.88E-75 | 3.34E-74 | up |
| *Cd59b* | chr2:104089324 | Intron | 6.757569312 | 2.18E-74 | 1.23E-73 | up |
| *Gtdc1* | chr2:44613412 | Intron | 6.740719549 | 3.58E-38 | 1.35E-37 | up |
| *Gm17484* | chr8:25762722 | Intron | 6.733101598 | 3.23E-55 | 1.48E-54 | up |
| *6330403L08Rik* | chr5:138997640 | exon | 6.726150158 | 1.46E-126 | 1.24E-125 | up |
| *Aim2* | chr1:173400302 | Intron | 6.701453635 | 2.19E-67 | 1.16E-66 | up |
| *Gm7534* | chr4:134193643 | Intron | 6.700828741 | 3.11E-09 | 5.07E-09 | up |
| *Gm12031* | chr11:20146227 | exon | 6.681946318 | 7.88E-147 | 7.67E-146 | up |
| *Zfp808* | chr13:62183211 | Intron | 6.67804624 | 3.22E-35 | 1.16E-34 | up |
| *Gm15728* | chr5:117391453 | exon | 6.668208632 | 2.66E-46 | 1.13E-45 | up |
| *Phc1* | chr6:122337235 | five_prime_utr | 6.663816379 | 5.81E-67 | 3.05E-66 | up |
| *Chl1* | chr6:103579478 | Intron | 6.660684849 | 5.54E-115 | 4.29E-114 | up |
| *Poc1b* | chr10:99205265 | intergenic(10kb) | 6.62634422 | 1.45E-201 | 2.02E-200 | up |
| *Zbtb6* | chr2:37431312 | Intron | 6.621266505 | 2.11E-82 | 1.28E-81 | up |
| *Gm21123* | chr7:110690604 | exon | 6.598867401 | 2.72E-153 | 2.84E-152 | up |
| *Eif5a* | chr11:69916726 | Intron | 6.579573184 | 3.45E-45 | 1.45E-44 | up |
| *Golm1* | chr13:59654989 | Intron | 6.566047641 | 1.31E-34 | 4.70E-34 | up |
| *Trpm3* | chr19:22728442 | Intron | 6.546051316 | 0.00155162 | 0.00189395 | up |
| *Lck* | chr4:129554482 | Intron | 6.543548863 | 3.07E-52 | 1.37E-51 | up |
| *Tdp2* | chr13:24838552 | Intron | 6.540679893 | 7.98E-21 | 1.93E-20 | up |
| *Ryr2* | chr13:11907225 | Intron | 6.521189844 | 3.56E-212 | 5.46E-211 | up |
| *Cisd2* | chr3:135409541 | Intron | 6.517840617 | 7.12E-118 | 5.70E-117 | up |
| *2310016D23Rik* | chr1:60788294 | Intron | 6.508874454 | 6.27E-35 | 2.26E-34 | up |
| *Nos1* | chr5:117884874 | Intron | 6.488910469 | 1.18E-254 | 2.30E-253 | up |
| *Atp9a* | chr2:168714664 | Intron | 6.466859361 | 1.03E-47 | 4.43E-47 | up |
| *0610040F04Rik* | chr6:108599347 | Intron | 6.423701437 | 1.18E-162 | 1.30E-161 | up |
| *Snrpa1* | chr7:66078135 | intergenic(10kb) | 6.422651726 | 4.88E-229 | 8.67E-228 | up |
| *Pdcl2* | chr5:76321536 | Intron | 6.359311069 | 5.29E-272 | 1.14E-270 | up |
| *Rimbp2* | chr5:128781532 | Intron | 6.329264223 | 2.28E-09 | 3.73E-09 | up |
| *Hsh2d* | chr8:72192004 | Intron | 6.314243092 | 4.70E-36 | 1.72E-35 | up |
| *Gnas* | chr2:174286308 | Intron | 6.313196661 | 2.62E-255 | 5.15E-254 | up |
| *C430039J16Rik* | chr13:97284399 | Intron | 6.30118542 | 1.05E-267 | 2.19E-266 | up |
| *Emc7* | chr2:112455297 | exon | 6.293206178 | 5.02E-240 | 9.08E-239 | up |
| *Sdk1* | chr5:141579066 | Intron | 6.291729998 | 1.21E-199 | 1.63E-198 | up |
| *Ralgapa2* | chr2:146300049 | Intron | 6.285951097 | 2.44E-219 | 3.95E-218 | up |
| *AC061963.3* | chr9:44921192 | Intron | 6.275122915 | 1.66E-267 | 3.42E-266 | up |
| *Evl* | chr12:108572005 | Intron | 6.240835049 | 6.12E-24 | 1.64E-23 | up |
| *Nup210l* | chr3:90108384 | Intron | 6.232334494 | 3.90E-215 | 6.09E-214 | up |
| *Sytl5* | chrX:9923604 | Intron | 6.224057129 | 1.11E-52 | 4.97E-52 | up |
| *Gm26631* | chr9:57532568 | exon | 6.20501851 | 4.06E-298 | 9.93E-297 | up |
| *Coro7* | chr16:4629629 | exon | 6.202788876 | 3.12E-227 | 5.43E-226 | up |
| *1700123O12Rik* | chr4:10494163 | Intron | 6.192709946 | 6.45E-92 | 4.22E-91 | up |
| *Prcp* | chr7:92915147 | Intron | 6.1123554 | 2.27E-24 | 6.16E-24 | up |
| *Gpr156* | chr16:37971927 | Intron | 6.08872377 | 1.98E-208 | 2.92E-207 | up |
| *Ints2* | chr11:86217424 | Intron | 6.068866789 | 5.47E-148 | 5.41E-147 | up |
| *Cenpf* | chr1:189660511 | Intron | 6.06584445 | 3.02E-19 | 7.03E-19 | up |
| *a* | chr2:154902517 | Intron | 6.057625051 | 1.26E-69 | 6.70E-69 | up |
| *Gm12248* | chr11:58091665 | Intron | 6.04430633 | 4.33E-153 | 4.50E-152 | up |
| *Slc25a16* | chr10:62921615 | Intron | 6.025866613 | 6.34E-222 | 1.06E-220 | up |
| *C330002G04Rik* | chr19:23039020 | Intron | 6.012215068 | 1.60E-223 | 2.74E-222 | up |
| *Ppt1* | chr4:122844031 | Intron | 6.010039509 | 2.10E-179 | 2.63E-178 | up |
| *Hectd1* | chr12:51763617 | Intron | 6.009885056 | 9.26E-05 | 0.000122199 | up |
| *Gm1330* | chr2:148995260 | Intron | 5.979678354 | 2.78E-62 | 1.41E-61 | up |
| *Rhbdl2* | chr4:123811267 | Intron | 5.964852344 | 8.35E-141 | 7.79E-140 | up |
| *Phf3* | chr1:30800013 | intergenic(10kb) | 5.954694688 | 3.13E-174 | 3.75E-173 | up |
| *Gm16223* | chr5:42137507 | Intron | 5.951474931 | 3.16E-169 | 3.62E-168 | up |
| *AC124413.2* | chr10:121514313 | Intron | 5.926687025 | 8.33E-275 | 1.88E-273 | up |
| *Gm19410* | chr8:35813435 | Intron | 5.925111271 | 7.02E-59 | 3.36E-58 | up |
| *Dirc2* | chr16:35739807 | Intron | 5.911806911 | 9.31E-222 | 1.55E-220 | up |
| *Cbr3* | chr16:93684102 | Intron | 5.909624165 | 1.63E-183 | 2.08E-182 | up |
| *Ephx1* | chr1:180979765 | Intron | 5.897364055 | 3.47E-280 | 8.05E-279 | up |
| *Gm5592* | chr7:41236778 | Intron | 5.891908216 | 7.84E-30 | 2.54E-29 | up |
| *Gm28376* | chr1:16938764 | Intron | 5.837907176 | 4.50E-41 | 1.80E-40 | up |
| *Cep55* | chr19:38058003 | Intron | 5.832708523 | 2.46E-256 | 4.88E-255 | up |
| *Klk7* | chr7:43816347 | three_prime_utr | 5.770632202 | 2.54E-111 | 1.89E-110 | up |
| *Rad23b* | chr4:55371506 | Intron | 5.752741612 | 6.20E-161 | 6.72E-160 | up |
| *Oas1b* | chr5:120822217 | Intron | 5.745222527 | 1.93E-24 | 5.26E-24 | up |
| *Gm2617* | chr19:9332175 | Intron | 5.721023985 | 5.90E-13 | 1.11E-12 | up |
| *Ncoa7* | chr10:30731535 | Intron | 5.715884671 | 1.18E-300 | 2.93E-299 | up |
| *Lrp1b* | chr2:40789551 | Intron | 5.709792353 | 1.40E-85 | 8.71E-85 | up |
| *2810410L24Rik* | chr11:120188764 | Intron | 5.699939968 | 9.55E-05 | 0.000125972 | up |
| *Slc25a43* | chrX:36768307 | Intron | 5.676318626 | 1.46E-176 | 1.77E-175 | up |
| *4930558J18Rik* | chr1:57377068 | Intron | 5.657079648 | 2.08E-269 | 4.37E-268 | up |
| *Ush2a* | chr1:188585212 | Intron | 5.647622031 | 5.00E-47 | 2.14E-46 | up |
| *Dgkg* | chr16:22544696 | Intron | 5.618315128 | 2.31E-179 | 2.86E-178 | up |
| *Timp3* | chr10:86334927 | Intron | 5.603038464 | 1.46E-30 | 4.77E-30 | up |
| *Prkag1* | chr15:98828516 | Intron | 5.570161019 | 1.06E-199 | 1.45E-198 | up |
| *Rbm3* | chrX:8143382 | Intron | 5.569429037 | 9.12E-116 | 7.08E-115 | up |
| *Xpnpep1* | chr19:52987820 | Intron | 5.566661741 | 1.36E-216 | 2.14E-215 | up |
| *Mms19* | chr19:41965384 | Intron | 5.519980159 | 6.02E-17 | 1.30E-16 | up |
| *Sparc* | chr11:55415132 | Intron | 5.479425464 | 3.21E-136 | 2.86E-135 | up |
| *Atg10* | chr13:90945780 | Intron | 5.441064992 | 8.23E-185 | 1.06E-183 | up |
| *Cmtr1* | chr17:29663714 | Intron | 5.421213545 | 7.97E-19 | 1.83E-18 | up |
| *5730507C01Rik* | chr12:18525042 | Intron | 5.419664558 | 1.58E-84 | 9.76E-84 | up |
| *Pik3c2g* | chr6:139657378 | gene | 5.417159381 | 4.17E-226 | 7.18E-225 | up |
| *Galnt10* | chr11:57680490 | Intron | 5.409711481 | 8.50E-287 | 2.02E-285 | up |
| *Lsm14a* | chr7:34369990 | Intron | 5.389651667 | 4.32E-177 | 5.28E-176 | up |
| *Ppfia2* | chr10:106810310 | Intron | 5.385813214 | 4.35E-18 | 9.71E-18 | up |
| *Cers6* | chr2:68938801 | Intron | 5.369506775 | 4.83E-274 | 1.08E-272 | up |
| *Tmem44* | chr16:30544279 | Intron | 5.359499118 | 3.22E-132 | 2.83E-131 | up |
| *Gm14223* | chr2:159937061 | Intron | 5.352209286 | 4.97E-28 | 1.54E-27 | up |
| *Zfp961* | chr8:71951580 | Intron | 5.334852654 | 1.03E-209 | 1.55E-208 | up |
| *Gm14444* | chr2:175016444 | Intron | 5.334295326 | 5.31E-53 | 2.40E-52 | up |
| *Hdac7* | chr15:97803999 | Intron | 5.306176996 | 6.69E-23 | 1.74E-22 | up |
| *Gm6522* | chr3:106287156 | Intron | 5.292035173 | 4.94E-23 | 1.29E-22 | up |
| *Ehbp1* | chr11:22338304 | Intron | 5.277391749 | 3.59E-27 | 1.06E-26 | up |
| *Ube3c* | chr5:29576631 | Intron | 5.272746686 | 6.17E-218 | 9.89E-217 | up |
| *Pag1* | chr3:9810110 | Intron | 5.272017398 | 1.39E-74 | 7.86E-74 | up |
| *Ppm1l* | chr3:69361160 | Intron | 5.267611978 | 5.52E-223 | 9.34E-222 | up |
| *Cntn1* | chr15:92319898 | Intron | 5.261093377 | 2.56E-113 | 1.95E-112 | up |
| *Hmox2* | chr16:4746436 | Intron | 5.230466438 | 2.20E-11 | 3.87E-11 | up |
| *Snd1* | chr6:28499564 | Intron | 5.22599012 | 1.50E-34 | 5.36E-34 | up |
| *Elmo1* | chr13:20133924 | Intron | 5.225394846 | 1.19E-285 | 2.80E-284 | up |
| *Astn1* | chr1:158648138 | Intron | 5.222509364 | 5.24E-11 | 9.10E-11 | up |
| *Epb41l1* | chr2:156423179 | Intron | 5.204259847 | 6.11E-279 | 1.40E-277 | up |
| *Atp11b* | chr3:35769443 | Intron | 5.20221496 | 9.22E-181 | 1.16E-179 | up |
| *Nfib* | chr4:82392624 | Intron | 5.199334621 | 2.85E-147 | 2.81E-146 | up |
| *Gm20005* | chr5:149643152 | Intron | 5.191816226 | 2.53E-117 | 2.01E-116 | up |
| *2610203C22Rik* | chr1:9588655 | Intron | 5.188198224 | 7.18E-82 | 4.33E-81 | up |
| *Atp1b3* | chr9:96363518 | Intron | 5.171070524 | 6.28E-83 | 3.83E-82 | up |
| *Slc36a3* | chr11:55127296 | Intron | 5.166955182 | 3.26E-217 | 5.17E-216 | up |
| *Agps* | chr2:75931309 | three_prime_utr | 5.14907012 | 1.74E-197 | 2.34E-196 | up |
| *4732471J01Rik* | chr7:25427944 | Intron | 5.146510603 | 7.34E-47 | 3.14E-46 | up |
| *Tmprss15* | chr16:78988792 | Intron | 5.107128526 | 1.37E-09 | 2.26E-09 | up |
| *Chd9* | chr8:90904186 | Intron | 5.092651917 | 8.34E-250 | 1.57E-248 | up |
| *Bmpr1a* | chr14:34455362 | Intron | 5.087237596 | 4.99E-272 | 1.09E-270 | up |
| *Gm11630* | chr11:97893264 | Intron | 5.060067597 | 1.65E-150 | 1.68E-149 | up |
| *Mettl26* | chr17:25880497 | gene | 5.052445086 | 2.15E-173 | 2.56E-172 | up |
| *Pard3* | chr8:127508456 | Intron | 5.047558495 | 9.21E-220 | 1.50E-218 | up |
| *Gm45846* | chr7:123123841 | Intron | 5.041875174 | 1.45E-54 | 6.59E-54 | up |
| *AC154994.1* | chr9:9395273 | Intron | 5.041871569 | 5.80E-149 | 5.80E-148 | up |
| *Gm14005* | chr2:128339557 | Intron | 5.038939333 | 2.71E-94 | 1.81E-93 | up |
| *Man1a* | chr10:53929954 | Intron | 5.024206198 | 5.09E-207 | 7.25E-206 | up |
| *Alg13* | chrX:144339564 | Intron | 5.021408771 | 3.07E-179 | 3.78E-178 | up |
| *Galnt7* | chr8:57633167 | Intron | 5.018248312 | 6.19E-152 | 6.40E-151 | up |
| *Gm10640* | chr7:31157024 | Intron | 5.01647357 | 3.64E-57 | 1.71E-56 | up |
| *Timm17a* | chr1:135313532 | Intron | 5.009070975 | 2.47E-13 | 4.71E-13 | up |
| *Gm14051* | chr2:132208649 | Intron | 5.00702137 | 3.71E-147 | 3.63E-146 | up |
| *Dtx1* | chr5:120701754 | Intron | 5.004521987 | 1.41E-172 | 1.67E-171 | up |
| *Wdr20* | chr12:110759021 | Intron | 4.996814387 | 4.31E-148 | 4.29E-147 | up |
| *Strn* | chr17:78670164 | Intron | 4.988581092 | 1.83E-142 | 1.72E-141 | up |
| *Rbm26* | chr14:105148721 | Intron | 4.980596227 | 4.46E-57 | 2.09E-56 | up |
| *Crebl2* | chr6:134843143 | Intron | 4.958970398 | 8.24E-202 | 1.16E-200 | up |
| *Nt5dc1* | chr10:34315133 | Intron | 4.955614775 | 2.78E-187 | 3.59E-186 | up |
| *Ret* | chr6:118167455 | Intron | 4.953662598 | 1.23E-16 | 2.64E-16 | up |
| *Vmn2r-ps25* | chr5:151453901 | Intron | 4.930947735 | 1.54E-13 | 2.95E-13 | up |
| *Slc38a11* | chr2:65341608 | Intron | 4.915073293 | 1.35E-112 | 1.02E-111 | up |
| *AC102009.1* | chr10:11581795 | Intron | 4.910796102 | 5.69E-16 | 1.19E-15 | up |
| *Larp6* | chr9:60729242 | Intron | 4.888949262 | 8.57E-18 | 1.89E-17 | up |
| *Tmem57* | chr4:134852369 | Intron | 4.888933944 | 2.20E-149 | 2.21E-148 | up |
| *4930567H12Rik* | chr8:125604729 | Intron | 4.885447087 | 5.72E-93 | 3.77E-92 | up |
| *Taf11* | chr17:27904426 | Intron | 4.884077136 | 4.46E-77 | 2.60E-76 | up |
| *Bach2* | chr4:32240484 | Intron | 4.881984428 | 2.08E-166 | 2.34E-165 | up |
| *Mrpl33* | chr5:31633943 | Intron | 4.862933472 | 1.05E-108 | 7.70E-108 | up |
| *Mecomos* | chr3:30049860 | exon | 4.830559808 | 8.45E-147 | 8.18E-146 | up |
| *Olfr362* | chr2:37110523 | Intron | 4.829817902 | 8.83E-25 | 2.46E-24 | up |
| *Zfp182* | chrX:21058453 | Intron | 4.827992839 | 6.76E-163 | 7.46E-162 | up |
| *Slc28a2* | chr2:122456541 | Intron | 4.827137784 | 2.30E-23 | 6.11E-23 | up |
| *Slco1a6* | chr6:142153029 | Intron | 4.797734764 | 1.20E-12 | 2.23E-12 | up |
| *F930017D23Rik* | chr10:43598636 | Intron | 4.792700866 | 7.37E-130 | 6.35E-129 | up |
| *Map4k5* | chr12:69850635 | Intron | 4.777866452 | 5.20E-129 | 4.46E-128 | up |
| *Platr2* | chr13:65787756 | Intron | 4.77652649 | 3.14E-23 | 8.30E-23 | up |
| *Nt5dc3* | chr10:86798817 | Intron | 4.767652966 | 2.84E-13 | 5.39E-13 | up |
| *Vmn2r27* | chr6:124214057 | Intron | 4.764018281 | 8.92E-20 | 2.10E-19 | up |
| *Foxn3* | chr12:99515871 | Intron | 4.762132221 | 3.94E-200 | 5.41E-199 | up |
| *BC049762* | chr11:51256547 | Intron | 4.751791753 | 7.41E-151 | 7.62E-150 | up |
| *AC154517.3* | chr12:13423943 | exon | 4.751304865 | 8.43E-11 | 1.46E-10 | up |
| *Etv5* | chr16:22433851 | Intron | 4.727003547 | 1.31E-187 | 1.71E-186 | up |
| *Tmem63c* | chr12:87050932 | Intron | 4.720262479 | 7.23E-14 | 1.41E-13 | up |
| *Esr1* | chr10:4789324 | Intron | 4.72013754 | 1.77E-39 | 6.85E-39 | up |
| *Igsf3* | chr3:101437002 | Intron | 4.706296305 | 1.11E-33 | 3.94E-33 | up |
| *Saxo1* | chr4:86470906 | Intron | 4.687388796 | 4.23E-21 | 1.04E-20 | up |
| *Arel1* | chr12:84962064 | Intron | 4.682717424 | 2.71E-208 | 3.95E-207 | up |
| *Accs* | chr2:93843749 | Intron | 4.665317166 | 3.94E-66 | 2.04E-65 | up |
| *Fut8* | chr12:77264813 | Intron | 4.657486373 | 1.89E-99 | 1.29E-98 | up |
| *Tex21* | chr12:76222975 | Intron | 4.654793962 | 5.67E-158 | 6.03E-157 | up |
| *Ppm1h* | chr10:122792790 | Intron | 4.651574981 | 1.19E-61 | 6.02E-61 | up |
| *Agtrap* | chr4:148087131 | Intron | 4.649242144 | 1.21E-121 | 9.83E-121 | up |
| *Ankrd35* | chr3:96679461 | Intron | 4.643657418 | 4.36E-188 | 5.76E-187 | up |
| *Fam83g* | chr11:61709027 | Intron | 4.639970982 | 9.26E-125 | 7.73E-124 | up |
| *AC154687.2* | chr14:68050047 | Intron | 4.636351712 | 9.02E-62 | 4.56E-61 | up |
| *Kcnh1* | chr1:192289256 | Intron | 4.623078829 | 1.53E-85 | 9.48E-85 | up |
| *Desi2* | chr1:178214896 | Intron | 4.608847867 | 1.06E-11 | 1.90E-11 | up |
| *Zc3h7b* | chr15:81770858 | Intron | 4.594236333 | 3.13E-45 | 1.33E-44 | up |
| *Ubap2l* | chr3:90048298 | Intron | 4.589418333 | 3.62E-59 | 1.74E-58 | up |
| *Rab6b* | chr9:103117388 | Intron | 4.582095947 | 1.03E-214 | 1.59E-213 | up |
| *Snhg14* | chr7:59689141 | Intron | 4.558059455 | 2.00E-51 | 8.89E-51 | up |
| *Asap1* | chr15:64213900 | Intron | 4.552446707 | 4.12E-140 | 3.78E-139 | up |
| *Meltf* | chr16:31878847 | exon | 4.549674329 | 2.00E-168 | 2.28E-167 | up |
| *St8sia1* | chr6:142898848 | Intron | 4.52890343 | 1.82E-14 | 3.63E-14 | up |
| *Rrm2b* | chr15:37960269 | Intron | 4.524576225 | 2.28E-121 | 1.84E-120 | up |
| *Hltf* | chr3:20094562 | Intron | 4.521338329 | 5.99E-20 | 1.42E-19 | up |
| *Slc22a27* | chr19:7937877 | Intron | 4.521053748 | 1.42E-12 | 2.62E-12 | up |
| *Gm11099* | chr2:58881183 | Intron | 4.5202225 | 5.81E-67 | 3.05E-66 | up |
| *AC155634.2* | chr12:64183556 | Intron | 4.517348349 | 1.43E-17 | 3.15E-17 | up |
| *Gm38366* | chr9:108531954 | exon | 4.497938018 | 1.71E-84 | 1.05E-83 | up |
| *CT010460.1* | chr12:71427441 | Intron | 4.491884678 | 6.78E-145 | 6.46E-144 | up |
| *Gm15218* | chr14:45835891 | Intron | 4.489727351 | 1.17E-116 | 9.23E-116 | up |
| *1700073E17Rik* | chr6:145389614 | Intron | 4.489296565 | 5.15E-94 | 3.42E-93 | up |
| *Trove2* | chr1:143752007 | three_prime_utr | 4.476794071 | 3.19E-94 | 2.13E-93 | up |
| *Acyp2* | chr11:30522000 | Intron | 4.459750666 | 1.06E-107 | 7.68E-107 | up |
| *Rufy3* | chr5:88620247 | Intron | 4.443895 | 4.03E-209 | 6.03E-208 | up |
| *Vmn2r109* | chr17:20561981 | Intron | 4.442067626 | 2.42E-187 | 3.15E-186 | up |
| *Rspry1* | chr8:94649806 | CDS | 4.434432656 | 6.17E-106 | 4.43E-105 | up |
| *Gm12023* | chr11:18638136 | Intron | 4.433297878 | 1.42E-19 | 3.33E-19 | up |
| *Gsta2* | chr9:78349946 | Intron | 4.421319097 | 1.93E-91 | 1.26E-90 | up |
| *Paics* | chr5:76953083 | Intron | 4.42110983 | 2.06E-157 | 2.18E-156 | up |
| *Slc25a25* | chr2:32447579 | Intron | 4.408341376 | 7.65E-08 | 1.18E-07 | up |
| *Skap2* | chr6:51881950 | Intron | 4.404080471 | 1.49E-182 | 1.89E-181 | up |
| *Ano4* | chr10:89303354 | Intron | 4.396970945 | 2.12E-130 | 1.85E-129 | up |
| *Itga7* | chr10:128945228 | Intron | 4.377260004 | 1.23E-37 | 4.63E-37 | up |
| *Zcchc11* | chr4:108522490 | Intron | 4.375234085 | 5.42E-112 | 4.07E-111 | up |
| *AC158686.2* | chr10:119610670 | Intron | 4.372857845 | 7.86E-97 | 5.33E-96 | up |
| *Nedd4l* | chr18:65037377 | Intron | 4.358115294 | 4.72E-75 | 2.69E-74 | up |
| *Gm15668* | chr1:67310767 | Intron | 4.338592683 | 2.36E-109 | 1.75E-108 | up |
| *Agap1* | chr1:89508969 | Intron | 4.319214694 | 1.21E-140 | 1.12E-139 | up |
| *Srgap3* | chr6:112764314 | Intron | 4.31896166 | 1.48E-140 | 1.36E-139 | up |
| *Kcnk10* | chr12:98484853 | Intron | 4.2983785 | 2.12E-30 | 6.90E-30 | up |
| *AC157019.2* | chr10:15794044 | Intron | 4.260331228 | 9.13E-167 | 1.03E-165 | up |
| *Mrps5* | chr2:127589717 | Intron | 4.24908719 | 3.25E-122 | 2.65E-121 | up |
| *Fam129a* | chr1:151580214 | Intron | 4.237750795 | 5.86E-67 | 3.07E-66 | up |
| *Ovch2* | chr7:107787302 | Intron | 4.216379061 | 3.34E-61 | 1.66E-60 | up |
| *Tab3* | chrX:85584711 | Intron | 4.212616752 | 9.73E-14 | 1.89E-13 | up |
| *Sec1* | chr7:45682485 | Intron | 4.193048734 | 6.24E-138 | 5.62E-137 | up |
| *Nyap2* | chr1:81301861 | Intron | 4.181765274 | 1.28E-86 | 8.18E-86 | up |
| *Rhpn2* | chr7:35365280 | Intron | 4.160182376 | 1.05E-111 | 7.83E-111 | up |
| *Med13l* | chr5:118568654 | Intron | 4.159290145 | 1.79E-162 | 1.95E-161 | up |
| *Lpar5* | chr6:125072813 | Intron | 4.151490011 | 3.33E-30 | 1.08E-29 | up |
| *Ccdc38* | chr10:93544552 | Intron | 4.136653784 | 3.93E-105 | 2.79E-104 | up |
| *Rpgrip1* | chr14:52113151 | Intron | 4.135913223 | 9.59E-133 | 8.47E-132 | up |
| *Stag2* | chrX:42175986 | Intron | 4.131145156 | 1.63E-60 | 7.96E-60 | up |
| *Gm5709* | chr3:59628262 | Intron | 4.120689696 | 3.31E-20 | 7.89E-20 | up |
| *Syt1* | chr10:108582047 | Intron | 4.099452038 | 1.65E-150 | 1.68E-149 | up |
| *Myo16* | chr8:10283394 | Intron | 4.082432697 | 2.19E-67 | 1.16E-66 | up |
| *Sorbs1* | chr19:40348696 | Intron | 4.079102748 | 1.27E-124 | 1.05E-123 | up |
| *Cyyr1* | chr16:85429488 | Intron | 4.073662809 | 9.67E-143 | 9.12E-142 | up |
| *Gm31243* | chr3:135926583 | Intron | 4.072227993 | 7.88E-50 | 3.47E-49 | up |
| *Galm* | chr17:80169003 | Intron | 4.071779591 | 4.64E-40 | 1.81E-39 | up |
| *Dmd* | chrX:82975495 | Intron | 4.067217557 | 4.76E-37 | 1.76E-36 | up |
| *Pknox1* | chr17:31570435 | Intron | 4.059439433 | 1.48E-169 | 1.71E-168 | up |
| *Ptges2* | chr2:32404777 | exon | 4.055779835 | 1.84E-76 | 1.07E-75 | up |
| *Dlg2* | chr7:91989740 | Intron | 4.051310622 | 1.36E-145 | 1.31E-144 | up |
| *Cog5* | chr12:31866033 | Intron | 4.050353678 | 2.03E-26 | 5.91E-26 | up |
| *Erich2* | chr2:70525968 | Intron | 4.046744896 | 3.87E-124 | 3.20E-123 | up |
| *Tprg* | chr16:25415650 | Intron | 4.032748582 | 1.90E-71 | 1.03E-70 | up |
| *Cx3cr1* | chr9:119985180 | Intron | 4.028710421 | 1.77E-37 | 6.60E-37 | up |
| *Pfdn1* | chr18:36418120 | Intron | 4.028631568 | 1.10E-149 | 1.11E-148 | up |
| *Syngr1* | chr15:80094562 | Intron | 4.021491061 | 5.14E-132 | 4.50E-131 | up |
| *Iqcm* | chr8:75495835 | Intron | 4.016649109 | 4.87E-75 | 2.77E-74 | up |
| *D2hgdh* | chr1:93843622 | Intron | 4.003546164 | 3.76E-23 | 9.88E-23 | up |
| *Msra* | chr14:64166948 | Intron | 3.996653435 | 1.89E-138 | 1.72E-137 | up |
| *Tmem117* | chr15:94801569 | Intron | 3.978424253 | 2.12E-176 | 2.56E-175 | up |
| *Mettl15* | chr2:109210603 | Intron | 3.965916715 | 3.98E-73 | 2.22E-72 | up |
| *Cpa6* | chr1:10703175 | Intron | 3.964307343 | 3.07E-91 | 1.99E-90 | up |
| *3110082I17Rik* | chr5:139452362 | Intron | 3.962749929 | 2.73E-125 | 2.28E-124 | up |
| *Synpo* | chr18:60616428 | Intron | 3.95811601 | 3.24E-103 | 2.27E-102 | up |
| *4930558C23Rik* | chr3:95400140 | Intron | 3.946750365 | 4.47E-140 | 4.08E-139 | up |
| *Edf1* | chr2:25560087 | Intron | 3.945620771 | 6.27E-116 | 4.89E-115 | up |
| *Hsf3* | chrX:96384272 | Intron | 3.942202718 | 1.00E-13 | 1.95E-13 | up |
| *Sytl2* | chr7:90416789 | intergenic(10kb) | 3.929072405 | 2.44E-37 | 9.08E-37 | up |
| *Phf2* | chr13:48841342 | Intron | 3.914570113 | 3.31E-130 | 2.87E-129 | up |
| *Prkcq* | chr2:11298851 | Intron | 3.898761033 | 1.56E-117 | 1.25E-116 | up |
| *St14* | chr9:31117028 | Intron | 3.895387615 | 3.53E-96 | 2.38E-95 | up |
| *Pibf1* | chr14:99251564 | Intron | 3.892234543 | 6.85E-94 | 4.52E-93 | up |
| *Gm15925* | chr7:3791392 | Intron | 3.888954499 | 4.97E-28 | 1.54E-27 | up |
| *Dlgap2* | chr8:14107755 | Intron | 3.885210677 | 1.12E-119 | 8.98E-119 | up |
| *C330007P06Rik* | chrX:36817549 | intergenic(10kb) | 3.882977006 | 3.23E-56 | 1.50E-55 | up |
| *Sntg2* | chr12:30189114 | Intron | 3.872270479 | 2.67E-21 | 6.61E-21 | up |
| *Epb41l2* | chr10:25379920 | Intron | 3.872124183 | 7.53E-17 | 1.62E-16 | up |
| *Fgd3* | chr13:49286513 | Intron | 3.845208452 | 8.21E-56 | 3.79E-55 | up |
| *Ust* | chr10:8528004 | intergenic(10kb) | 3.832119229 | 1.30E-40 | 5.16E-40 | up |
| *Gm18129* | chr7:60275619 | Intron | 3.830375435 | 1.23E-13 | 2.38E-13 | up |
| *Nsmce4a* | chr7:130543566 | Intron | 3.827306271 | 9.38E-07 | 1.36E-06 | up |
| *Pbx3* | chr2:34271856 | Intron | 3.825680687 | 5.46E-113 | 4.15E-112 | up |
| *Muc16* | chr9:18506144 | Intron | 3.806294853 | 5.81E-18 | 1.29E-17 | up |
| *Tmem214* | chr5:30868981 | five_prime_utr | 3.796878409 | 5.94E-76 | 3.41E-75 | up |
| *Prep* | chr10:45070551 | Intron | 3.794735667 | 5.13E-134 | 4.55E-133 | up |
| *Sh3d19* | chr3:85973847 | Intron | 3.793152089 | 4.46E-92 | 2.92E-91 | up |
| *Acvr1b* | chr15:101176190 | Intron | 3.784020681 | 1.12E-114 | 8.63E-114 | up |
| *Rap1gap* | chr4:137714280 | Intron | 3.782763386 | 1.06E-89 | 6.86E-89 | up |
| *Rab5b* | chr10:128690879 | Intron | 3.769381398 | 5.59E-63 | 2.84E-62 | up |
| *Gm14326* | chr2:177948749 | Intron | 3.756837636 | 1.97E-10 | 3.36E-10 | up |
| *Nusap1* | chr2:119619492 | Intron | 3.755818231 | 4.12E-100 | 2.82E-99 | up |
| *Nfe2l2* | chr2:75682997 | Intron | 3.752475518 | 5.07E-106 | 3.65E-105 | up |
| *4930433N12Rik* | chr9:3147528 | Intron | 3.745145393 | 9.49E-28 | 2.90E-27 | up |
| *Pecam1* | chr11:106682457 | Intron | 3.73349581 | 1.23E-159 | 1.33E-158 | up |
| *Slc16a10* | chr10:40125938 | Intron | 3.728560212 | 2.07E-126 | 1.75E-125 | up |
| *D230025D16Rik* | chr8:105235045 | Intron | 3.712535248 | 1.19E-128 | 1.02E-127 | up |
| *Slc18b1* | chr10:23819494 | Intron | 3.707711675 | 4.12E-72 | 2.25E-71 | up |
| *Tacr1* | chr6:82466416 | Intron | 3.692062595 | 1.00E-53 | 4.54E-53 | up |
| *Nrg4* | chr9:55252366 | Intron | 3.691580054 | 2.44E-74 | 1.37E-73 | up |
| *Lgals8* | chr13:12441149 | Intron | 3.687819133 | 8.88E-107 | 6.43E-106 | up |
| *Sertad2* | chr11:20596876 | Intron | 3.683937931 | 3.81E-16 | 8.02E-16 | up |
| *Med27* | chr2:29438468 | Intron | 3.683885459 | 1.51E-104 | 1.06E-103 | up |
| *Iqca* | chr1:90062484 | Intron | 3.678619568 | 5.77E-86 | 3.61E-85 | up |
| *Prkag2* | chr5:24983666 | Intron | 3.649538303 | 3.28E-86 | 2.08E-85 | up |
| *AC110381.2* | chr10:122792055 | exon | 3.63097152 | 8.79E-106 | 6.29E-105 | up |
| *Zfp516* | chr18:82974711 | Intron | 3.62551254 | 3.80E-86 | 2.39E-85 | up |
| *Fstl1* | chr16:37809138 | Intron | 3.622582125 | 3.93E-101 | 2.73E-100 | up |
| *Gfra1* | chr19:58359579 | Intron | 3.608262797 | 3.47E-123 | 2.85E-122 | up |
| *Epb41l3* | chr17:69150850 | Intron | 3.581416891 | 5.17E-61 | 2.57E-60 | up |
| *Msi2* | chr11:88563653 | Intron | 3.577210977 | 6.64E-143 | 6.30E-142 | up |
| *Zfp652* | chr11:95765307 | Intron | 3.558545233 | 5.32E-108 | 3.88E-107 | up |
| *Snhg5* | chr9:88522019 | exon | 3.553119445 | 2.19E-109 | 1.63E-108 | up |
| *Zcchc24* | chr14:25730259 | Intron | 3.55062008 | 3.78E-05 | 5.08E-05 | up |
| *Top3b* | chr16:16877491 | Intron | 3.545665765 | 6.27E-35 | 2.26E-34 | up |
| *Clec2g* | chr6:128938094 | Intron | 3.529517212 | 3.68E-109 | 2.71E-108 | up |
| *Gm27017* | chr14:61650683 | Intron | 3.528294738 | 2.19E-82 | 1.33E-81 | up |
| *Gm31816* | chr9:47318910 | Intron | 3.521749473 | 6.88E-43 | 2.82E-42 | up |
| *Egfl6* | chrX:166525992 | Intron | 3.507124074 | 3.03E-59 | 1.46E-58 | up |
| *Cryl1* | chr14:57282192 | Intron | 3.503166162 | 1.79E-87 | 1.15E-86 | up |
| *Il31ra* | chr13:112578632 | Intron | 3.493123625 | 8.18E-45 | 3.42E-44 | up |
| *Dxo* | chr17:34838759 | CDS | 3.480933735 | 2.36E-67 | 1.24E-66 | up |
| *Adra1a* | chr14:66670320 | Intron | 3.477582944 | 4.65E-73 | 2.58E-72 | up |
| *Dynlt1b* | chr17:6433966 | Intron | 3.465581344 | 6.70E-105 | 4.74E-104 | up |
| *Asl* | chr5:130012315 | Intron | 3.450577905 | 1.26E-69 | 6.70E-69 | up |
| *Acap1* | chr11:69883430 | Intron | 3.43446343 | 1.37E-80 | 8.19E-80 | up |
| *Tdh* | chr14:63503127 | Intron | 3.426141508 | 1.05E-72 | 5.81E-72 | up |
| *Myh9* | chr15:77826941 | Intron | 3.408477352 | 3.92E-50 | 1.73E-49 | up |
| *Dpp10* | chr1:123872993 | Intron | 3.387076 | 2.43E-06 | 3.48E-06 | up |
| *Gm11823* | chr4:13941811 | Intron | 3.386578873 | 6.15E-07 | 9.03E-07 | up |
| *Bnc2* | chr4:84547198 | Intron | 3.364934226 | 1.28E-61 | 6.43E-61 | up |
| *Apobec2* | chr17:48430551 | Intron | 3.360985919 | 1.62E-70 | 8.65E-70 | up |
| *Bcat1* | chr6:145047572 | Intron | 3.35915269 | 1.97E-72 | 1.08E-71 | up |
| *Gm37359* | chr3:65871693 | Intron | 3.358870845 | 1.34E-83 | 8.18E-83 | up |
| *AC173481.1* | chr13:6673332 | exon | 3.357995048 | 0.000121055 | 0.00015866 | up |
| *Pign* | chr1:105569928 | Intron | 3.349363264 | 8.52E-18 | 1.89E-17 | up |
| *C430014B12Rik* | chr1:82724745 | exon | 3.33941887 | 1.57E-78 | 9.23E-78 | up |
| *Mbnl1* | chr3:60494345 | Intron | 3.333960039 | 9.81E-105 | 6.91E-104 | up |
| *Slc9a7* | chrX:20168491 | Intron | 3.333022012 | 9.38E-61 | 4.60E-60 | up |
| *Zfp710* | chr7:80085592 | Intron | 3.327182531 | 3.26E-86 | 2.07E-85 | up |
| *Ostf1* | chr19:18613909 | Intron | 3.317398091 | 3.03E-89 | 1.95E-88 | up |
| *Tnr* | chr1:159814464 | Intron | 3.316147562 | 1.12E-72 | 6.17E-72 | up |
| *Gm3164* | chr14:4435258 | Intron | 3.314576322 | 8.33E-13 | 1.56E-12 | up |
| *Vps13d* | chr4:145179642 | Intron | 3.30741604 | 1.08E-38 | 4.10E-38 | up |
| *2210408I21Rik* | chr13:77371144 | Intron | 3.307163795 | 1.77E-48 | 7.68E-48 | up |
| *Ankrd33b* | chr15:31352732 | Intron | 3.289147562 | 3.53E-86 | 2.22E-85 | up |
| *Mpp7* | chr18:7564447 | Intron | 3.278143071 | 9.06E-96 | 6.10E-95 | up |
| *Sel1l2* | chr2:140318456 | Intron | 3.274172921 | 2.48E-79 | 1.47E-78 | up |
| *Tpp2* | chr1:43937760 | Intron | 3.247613889 | 5.59E-72 | 3.04E-71 | up |
| *Gramd4* | chr15:86067508 | Intron | 3.246349524 | 2.93E-81 | 1.76E-80 | up |
| *AC164105.2* | chr9:46338915 | Intron | 3.241330031 | 1.37E-32 | 4.69E-32 | up |
| *Gm11775* | chr11:121039634 | Intron | 3.24087931 | 5.16E-59 | 2.48E-58 | up |
| *A630010A05Rik* | chr16:14573737 | Intron | 3.229585158 | 6.28E-05 | 8.34E-05 | up |
| *Arhgap32* | chr9:32192587 | Intron | 3.224911666 | 1.31E-114 | 1.00E-113 | up |
| *Nxn* | chr11:76328428 | Intron | 3.2212794 | 4.39E-116 | 3.44E-115 | up |
| *Pde2a* | chr7:101437800 | Intron | 3.214761374 | 2.68E-16 | 5.67E-16 | up |
| *Prdm1* | chr10:44459437 | Intron | 3.214149238 | 2.95E-80 | 1.76E-79 | up |
| *Nwd1* | chr8:72679656 | Intron | 3.213079938 | 4.29E-76 | 2.48E-75 | up |
| *Fyn* | chr10:39445850 | Intron | 3.210506541 | 1.02E-59 | 4.93E-59 | up |
| *Gm13335* | chr2:20430789 | Intron | 3.194407522 | 1.47E-05 | 2.02E-05 | up |
| *Depdc5* | chr5:32894974 | Intron | 3.193640598 | 1.75E-39 | 6.77E-39 | up |
| *Cyp2c53-ps* | chr19:39231564 | Intron | 3.191832113 | 4.26E-06 | 6.03E-06 | up |
| *Anks6* | chr4:47055190 | Intron | 3.183821058 | 6.27E-06 | 8.81E-06 | up |
| *AC226737.1* | chr10:130552613 | Intron | 3.179274902 | 4.72E-35 | 1.70E-34 | up |
| *A830080D01Rik* | chrX:159528369 | Intron | 3.178348514 | 3.60E-71 | 1.94E-70 | up |
| *Gm15494* | chr7:4459946 | Intron | 3.173191105 | 5.80E-19 | 1.34E-18 | up |
| *Herc4* | chr10:63284992 | Intron | 3.154280802 | 3.78E-06 | 5.36E-06 | up |
| *Rab3gap1* | chr1:127878115 | Intron | 3.130537523 | 6.12E-08 | 9.50E-08 | up |
| *A830019P07Rik* | chr19:35844566 | Intron | 3.120281701 | 4.94E-09 | 8.01E-09 | up |
| *Frmd5* | chr2:121765023 | Intron | 3.100745028 | 4.56E-71 | 2.45E-70 | up |
| *Sh3bp4* | chr1:89077983 | Intron | 3.095860167 | 2.82E-54 | 1.28E-53 | up |
| *Olfr138* | chr17:38269556 | Intron | 3.093425545 | 4.09E-07 | 6.07E-07 | up |
| *Cetn3* | chr13:81795916 | Intron | 3.09267804 | 1.45E-65 | 7.45E-65 | up |
| *Pcyt1b* | chrX:93744409 | Intron | 3.088447789 | 1.53E-100 | 1.05E-99 | up |
| *Nipal3* | chr4:135459836 | Intron | 3.086481865 | 6.26E-61 | 3.09E-60 | up |
| *Ascl4* | chr10:85931813 | Intron | 3.070620598 | 8.70E-79 | 5.13E-78 | up |
| *Yipf7* | chr5:69533902 | Intron | 3.069352849 | 7.78E-20 | 1.83E-19 | up |
| *Gm14548* | chr7:3889467 | Intron | 3.065400481 | 6.18E-16 | 1.29E-15 | up |
| *Rest* | chr5:77275154 | Intron | 3.061822104 | 3.55E-57 | 1.68E-56 | up |
| *Cnksr3* | chr10:7187840 | Intron | 3.057442097 | 3.05E-69 | 1.62E-68 | up |
| *AC156794.1* | chr9:80598898 | Intron | 3.050320725 | 2.05E-07 | 3.08E-07 | up |
| *Sirt3* | chr7:140881610 | exon | 3.041175602 | 5.93E-76 | 3.41E-75 | up |
| *Gm14149* | chr2:151208992 | Intron | 3.039330048 | 8.91E-22 | 2.24E-21 | up |
| *Got1* | chr19:43513902 | Intron | 3.010321393 | 3.37E-60 | 1.64E-59 | up |
| *A330015K06Rik* | chr3:65190677 | Intron | 3.009795149 | 7.42E-06 | 1.04E-05 | up |
| *Hs3st3a1* | chr11:64458033 | Intron | 3.009359278 | 0.000785633 | 0.000975306 | up |
| *Pglyrp1* | chr7:18872007 | Intron | 2.995231475 | 2.27E-31 | 7.61E-31 | up |
| *Igsf21* | chr4:140039221 | Intron | 2.979404214 | 1.17E-70 | 6.29E-70 | up |
| *Kcnh6* | chr11:106032803 | Intron | 2.974651204 | 2.52E-09 | 4.11E-09 | up |
| *Pcnx2* | chr8:125779056 | Intron | 2.95360533 | 1.15E-66 | 6.00E-66 | up |
| *Gm5946* | chrX:150221387 | exon | 2.952838775 | 2.50E-33 | 8.73E-33 | up |
| *Trp53rkb* | chr2:166794193 | three_prime_utr | 2.944192807 | 2.13E-29 | 6.84E-29 | up |
| *Gm30075* | chr7:73215927 | Intron | 2.942107333 | 1.02E-05 | 1.42E-05 | up |
| *Mast4* | chr13:102736411 | exon | 2.930767799 | 1.66E-36 | 6.09E-36 | up |
| *Gm2885* | chr17:6827303 | Intron | 2.928457189 | 1.26E-97 | 8.60E-97 | up |
| *Eya1* | chr1:14232918 | Intron | 2.914631518 | 8.25E-57 | 3.85E-56 | up |
| *Gm43376* | chr3:55920739 | Intron | 2.913280412 | 4.45E-05 | 5.98E-05 | up |
| *Slc37a1* | chr17:31298383 | Intron | 2.913049442 | 5.85E-73 | 3.24E-72 | up |
| *AC153829.1* | chr10:47070674 | Intron | 2.871705884 | 1.95E-08 | 3.08E-08 | up |
| *Gm3972* | chr7:9482887 | Intron | 2.870701936 | 1.28E-05 | 1.77E-05 | up |
| *Ap4s1* | chr12:51735989 | Intron | 2.867012766 | 2.03E-78 | 1.19E-77 | up |
| *Ubtd2* | chr11:32503605 | Intron | 2.861349855 | 5.00E-47 | 2.14E-46 | up |
| *Nemf* | chr12:69340077 | Intron | 2.84947303 | 1.17E-27 | 3.54E-27 | up |
| *Pga5* | chr19:10672068 | Intron | 2.846480142 | 1.48E-60 | 7.22E-60 | up |
| *AC125223.2* | chr13:38815643 | Intron | 2.830454024 | 1.52E-87 | 9.77E-87 | up |
| *Rrp12* | chr19:41889289 | Intron | 2.825878078 | 3.89E-09 | 6.32E-09 | up |
| *Hivep3* | chr4:119825760 | Intron | 2.819761293 | 3.56E-65 | 1.83E-64 | up |
| *Scn3a* | chr2:65567245 | Intron | 2.802155206 | 2.73E-63 | 1.39E-62 | up |
| *Ptprc* | chr1:138104404 | Intron | 2.798293381 | 3.16E-05 | 4.26E-05 | up |
| *Tbx3os1* | chr5:119587451 | Intron | 2.796605866 | 7.36E-68 | 3.91E-67 | up |
| *Gm13977* | chr2:115580107 | Intron | 2.789233424 | 2.79E-61 | 1.40E-60 | up |
| *Lrriq4* | chr3:30647235 | Intron | 2.788750867 | 1.85E-55 | 8.54E-55 | up |
| *Srpx* | chrX:10040809 | Intron | 2.772011183 | 1.18E-07 | 1.80E-07 | up |
| *Grin2b* | chr6:135827587 | Intron | 2.771318234 | 1.40E-67 | 7.40E-67 | up |
| *Gm43041* | chr5:59955774 | exon | 2.76066539 | 2.34E-12 | 4.29E-12 | up |
| *Chid1* | chr7:141512372 | Intron | 2.759675012 | 2.81E-58 | 1.34E-57 | up |
| *Gm7271* | chr5:76505903 | Intron | 2.739812888 | 1.73E-21 | 4.29E-21 | up |
| *Slc22a28* | chr19:8121264 | Intron | 2.732017681 | 1.60E-32 | 5.47E-32 | up |
| *Gm14410* | chr2:177196014 | Intron | 2.723289023 | 7.25E-06 | 1.01E-05 | up |
| *Gm43948* | chr6:101141449 | Intron | 2.719624671 | 1.07E-08 | 1.71E-08 | up |
| *Gpa33* | chr1:166133352 | Intron | 2.710378735 | 1.57E-58 | 7.50E-58 | up |
| *Blvra* | chr2:127079308 | Intron | 2.703895776 | 7.94E-61 | 3.91E-60 | up |
| *Olfr1388* | chr11:49445815 | three_prime_utr | 2.700954904 | 1.29E-11 | 2.30E-11 | up |
| *Mgat4c* | chr10:102194406 | Intron | 2.690081756 | 1.29E-10 | 2.21E-10 | up |
| *Catsperb* | chr12:101449076 | Intron | 2.67834494 | 7.61E-09 | 1.22E-08 | up |
| *Shroom2* | chrX:152644779 | Intron | 2.669347816 | 3.06E-34 | 1.09E-33 | up |
| *AC131117.2* | chr13:35631273 | Intron | 2.667302688 | 1.40E-07 | 2.12E-07 | up |
| *Arfgef3* | chr10:18625114 | Intron | 2.65920822 | 1.83E-66 | 9.54E-66 | up |
| *Slc1a6* | chr10:78807978 | Intron | 2.655201911 | 4.16E-05 | 5.60E-05 | up |
| *Meaf6* | chr4:125112461 | exon | 2.651925892 | 3.28E-36 | 1.20E-35 | up |
| *Stk33* | chr7:109370460 | Intron | 2.641687604 | 3.49E-17 | 7.61E-17 | up |
| *Irak2* | chr6:113689439 | Intron | 2.637143288 | 3.22E-35 | 1.16E-34 | up |
| *CT009754.2* | chr13:17934517 | Intron | 2.635404796 | 0.000182941 | 0.000237194 | up |
| *Phldb3* | chr7:24613964 | Intron | 2.626319253 | 1.67E-26 | 4.88E-26 | up |
| *Mboat1* | chr13:30187275 | Intron | 2.62566809 | 1.03E-47 | 4.43E-47 | up |
| *Mcm9* | chr10:53573583 | Intron | 2.61886468 | 0.000136718 | 0.000178801 | up |
| *Shroom3* | chr5:92876977 | Intron | 2.58187817 | 4.76E-74 | 2.67E-73 | up |
| *Kcns3* | chr12:11106705 | Intron | 2.57665287 | 1.95E-09 | 3.20E-09 | up |
| *Lats2* | chr14:57674663 | Intron | 2.572829957 | 2.75E-21 | 6.78E-21 | up |
| *Arhgap12* | chr18:6135357 | Intron | 2.569658791 | 5.46E-09 | 8.82E-09 | up |
| *Parva* | chr7:112492858 | Intron | 2.555547189 | 1.84E-33 | 6.48E-33 | up |
| *Frmpd4* | chrX:167559089 | Intron | 2.554030031 | 0.000275822 | 0.000353818 | up |
| *Gtsf1* | chr15:103412988 | Intron | 2.551234372 | 2.91E-05 | 3.94E-05 | up |
| *Nfs1* | chr2:156136588 | Intron | 2.551210986 | 4.79E-07 | 7.07E-07 | up |
| *Wdfy1* | chr1:79722694 | Intron | 2.550308818 | 0.000504115 | 0.000632766 | up |
| *Olfr54* | chr11:51017535 | Intron | 2.54965304 | 0.000404534 | 0.000512391 | up |
| *D830032E09Rik* | chr1:107925568 | Intron | 2.549453774 | 1.81E-06 | 2.61E-06 | up |
| *Gdf3* | chr6:122608740 | Intron | 2.548355943 | 1.64E-61 | 8.23E-61 | up |
| *Mipol1* | chr12:57473338 | Intron | 2.545653324 | 2.15E-40 | 8.46E-40 | up |
| *AC239878.2* | chr13:116617316 | Intron | 2.543801967 | 0.000749879 | 0.000931559 | up |
| *AC091783.1* | chr14:56193900 | Intron | 2.526968242 | 1.84E-07 | 2.77E-07 | up |
| *Pnrc1* | chr4:33277466 | Intron | 2.525067631 | 5.77E-61 | 2.85E-60 | up |
| *Gsk3b* | chr16:38092732 | Intron | 2.512460775 | 2.48E-39 | 9.56E-39 | up |
| *Arfgef2* | chr2:166815246 | Intron | 2.507289134 | 0.002231394 | 0.002699748 | up |
| *St8sia6* | chr2:13659312 | Intron | 2.492503884 | 6.09E-05 | 8.09E-05 | up |
| *Schip1* | chr3:68471933 | Intron | 2.491058768 | 9.83E-66 | 5.08E-65 | up |
| *AC124556.2* | chr12:76978679 | Intron | 2.481409946 | 1.65E-34 | 5.88E-34 | up |
| *Adamts17* | chr7:66883654 | Intron | 2.476093821 | 4.30E-41 | 1.72E-40 | up |
| *Gpc5* | chr14:115278116 | Intron | 2.471893202 | 1.03E-13 | 2.00E-13 | up |
| *Abca5* | chr11:110288393 | Intron | 2.471837446 | 1.74E-25 | 4.96E-25 | up |
| *Asap3* | chr4:136223416 | Intron | 2.470892269 | 4.24E-57 | 1.99E-56 | up |
| *Gm45459* | chr8:62809287 | Intron | 2.470157144 | 0.000442417 | 0.00055842 | up |
| *Ces5a* | chr8:93507554 | Intron | 2.469385658 | 8.66E-26 | 2.49E-25 | up |
| *Ptprt* | chr2:161989410 | Intron | 2.468844294 | 0.000309268 | 0.000395321 | up |
| *Tbc1d24* | chr17:24201003 | Intron | 2.467500903 | 1.50E-34 | 5.36E-34 | up |
| *Ago1* | chr4:126442618 | exon | 2.444869817 | 1.14E-43 | 4.71E-43 | up |
| *Gm15638* | chr16:45970232 | exon | 2.442599915 | 3.53E-50 | 1.57E-49 | up |
| *Ermard* | chr17:14979328 | Intron | 2.441947368 | 4.62E-31 | 1.54E-30 | up |
| *Dusp16* | chr6:134801886 | intergenic(10kb) | 2.435710913 | 1.12E-56 | 5.20E-56 | up |
| *Slc12a8* | chr16:33568523 | Intron | 2.42907242 | 4.17E-13 | 7.89E-13 | up |
| *L3hypdh* | chr12:72082723 | Intron | 2.426552353 | 2.82E-18 | 6.32E-18 | up |
| *Lrrc8d* | chr5:105739530 | Intron | 2.421618154 | 8.78E-39 | 3.34E-38 | up |
| *Cux1* | chr5:136356130 | Intron | 2.420971556 | 4.86E-33 | 1.69E-32 | up |
| *Synj1* | chr16:90994909 | Intron | 2.416497291 | 1.50E-24 | 4.13E-24 | up |
| *Cggbp1* | chr16:64856501 | three_prime_utr | 2.414999888 | 9.57E-58 | 4.55E-57 | up |
| *AC132237.3* | chr12:68347635 | Intron | 2.411510108 | 1.13E-10 | 1.95E-10 | up |
| *Cdkl1* | chr12:69758124 | Intron | 2.411098694 | 1.97E-33 | 6.92E-33 | up |
| *Zfp521* | chr18:13756614 | Intron | 2.409770851 | 2.67E-57 | 1.27E-56 | up |
| *Nbas* | chr12:13347391 | Intron | 2.407207104 | 9.68E-12 | 1.74E-11 | up |
| *Tbc1d12* | chr19:38844976 | Intron | 2.405258124 | 2.00E-26 | 5.84E-26 | up |
| *Gm29200* | chr18:12503745 | Intron | 2.400014631 | 0.000395996 | 0.000502631 | up |
| *AC166992.1* | chr9:21883214 | Intron | 2.398317122 | 0.000179278 | 0.000232778 | up |
| *Slit3* | chr11:35706151 | Intron | 2.395954725 | 1.17E-46 | 4.98E-46 | up |
| *Ip6k3* | chr17:27148777 | Intron | 2.390072404 | 2.09E-27 | 6.26E-27 | up |
| *BC147527* | chr13:120305207 | Intron | 2.389893259 | 1.33E-26 | 3.89E-26 | up |
| *Cyfip2* | chr11:46198145 | Intron | 2.374901287 | 2.66E-46 | 1.13E-45 | up |
| *Rbpms* | chr8:33902878 | Intron | 2.374260769 | 2.55E-64 | 1.30E-63 | up |
| *Gng7* | chr10:80977799 | Intron | 2.372315037 | 0.001292208 | 0.001585693 | up |
| *Ism1* | chr2:139748840 | Intron | 2.362931034 | 4.37E-33 | 1.52E-32 | up |
| *Lima1* | chr15:99863203 | Intron | 2.360314509 | 6.14E-40 | 2.39E-39 | up |
| *Magi2* | chr5:19478334 | Intron | 2.349475763 | 1.05E-18 | 2.41E-18 | up |
| *Shisa6* | chr11:66462699 | Intron | 2.347829065 | 3.55E-17 | 7.74E-17 | up |
| *Gm38825* | chr6:67203788 | Intron | 2.338472865 | 2.43E-49 | 1.07E-48 | up |
| *Pcsk6* | chr7:65866069 | Intron | 2.335236252 | 1.10E-37 | 4.13E-37 | up |
| *Gm7133* | chr1:97176320 | Intron | 2.328506986 | 1.15E-08 | 1.83E-08 | up |
| *Clhc1* | chr11:29566662 | Intron | 2.328131887 | 1.50E-31 | 5.02E-31 | up |
| *Gm17058* | chr15:99777568 | Intron | 2.326002097 | 3.19E-09 | 5.20E-09 | up |
| *Foxp2* | chr6:14964868 | Intron | 2.325865391 | 6.85E-12 | 1.24E-11 | up |
| *AC098716.1* | chr9:71195233 | Intron | 2.309563368 | 7.21E-05 | 9.55E-05 | up |
| *Tanc2* | chr11:105710006 | Intron | 2.297817825 | 1.81E-11 | 3.20E-11 | up |
| *Mc2r* | chr18:68416343 | Intron | 2.29504916 | 1.23E-05 | 1.70E-05 | up |
| *Mmp16* | chr4:17901595 | Intron | 2.294259717 | 1.33E-08 | 2.11E-08 | up |
| *Gm10338* | chr14:7590473 | Intron | 2.288066001 | 0.000977564 | 0.001206128 | up |
| *AC157494.1* | chr12:38050409 | Intron | 2.285496229 | 0.001121051 | 0.001378466 | up |
| *Rpgr* | chrX:10205167 | Intron | 2.279275593 | 1.16E-12 | 2.17E-12 | up |
| *Tfap2a* | chr13:40722703 | Intron | 2.2711309 | 7.80E-33 | 2.69E-32 | up |
| *Atp8a1* | chr5:67811224 | Intron | 2.26769151 | 3.45E-45 | 1.45E-44 | up |
| *Pam* | chr1:97867601 | Intron | 2.266476747 | 1.68E-11 | 2.98E-11 | up |
| *Gm29571* | chr10:25626938 | Intron | 2.260261098 | 1.46E-06 | 2.11E-06 | up |
| *Asap2* | chr12:21167416 | Intron | 2.25014416 | 1.49E-56 | 6.92E-56 | up |
| *Tfap2c* | chr2:172552226 | Intron | 2.2413748 | 7.77E-42 | 3.13E-41 | up |
| *Dip2b* | chr15:100126926 | Intron | 2.239028305 | 3.11E-42 | 1.25E-41 | up |
| *Dnajc8* | chr4:132549935 | Intron | 2.228234502 | 0.000584991 | 0.000731744 | up |
| *Map2* | chr1:66422074 | Intron | 2.227404746 | 5.80E-12 | 1.05E-11 | up |
| *Atp2b4* | chr1:133742279 | Intron | 2.226860934 | 1.23E-48 | 5.35E-48 | up |
| *Zfp367* | chr13:64149246 | Intron | 2.218657982 | 4.61E-28 | 1.43E-27 | up |
| *Myo1d* | chr11:80720186 | Intron | 2.217962031 | 0.000231442 | 0.000297945 | up |
| *Gm26561* | chr17:70971787 | Intron | 2.217586913 | 8.46E-43 | 3.46E-42 | up |
| *Gm10718* | chr9:3024416 | Intron | 2.216592406 | 4.62E-10 | 7.76E-10 | up |
| *Lcp1* | chr14:75178684 | Intron | 2.215108272 | 2.33E-42 | 9.44E-42 | up |
| *Spc25* | chr2:69205171 | five_prime_utr | 2.207582308 | 6.84E-31 | 2.25E-30 | up |
| *Lmx1a* | chr1:167797021 | Intron | 2.188511975 | 2.39E-05 | 3.25E-05 | up |
| *Ptrhd1* | chr12:4234593 | Intron | 2.187742236 | 0.001402165 | 0.00171713 | up |
| *AC140381.1* | chr10:120992034 | Intron | 2.185574118 | 9.99E-05 | 0.000131548 | up |
| *Cd84* | chr1:171860870 | Intron | 2.185360124 | 6.16E-37 | 2.27E-36 | up |
| *4930449A18Rik* | chr3:59800057 | Intron | 2.173103392 | 4.54E-05 | 6.09E-05 | up |
| *Pkp2* | chr16:16263773 | Intron | 2.170902562 | 1.26E-27 | 3.83E-27 | up |
| *Sycp2l* | chr13:41144925 | Intron | 2.168248413 | 3.11E-42 | 1.25E-41 | up |
| *Anks1b* | chr10:90736906 | Intron | 2.158318203 | 5.64E-46 | 2.39E-45 | up |
| *Grhl2* | chr15:37279708 | CDS | 2.155423624 | 2.31E-24 | 6.27E-24 | up |
| *Ankub1* | chr3:57686885 | Intron | 2.145452011 | 3.42E-08 | 5.35E-08 | up |
| *Irf3* | chr7:44999076 | Intron | 2.136969225 | 0.003166312 | 0.003792869 | up |
| *Aldh1a1* | chr19:20568566 | Intron | 2.134916415 | 0.001129655 | 0.001388103 | up |
| *Ogt* | chrX:101644632 | Intron | 2.133594308 | 2.68E-21 | 6.63E-21 | up |
| *Cep89* | chr7:35427404 | Intron | 2.126918274 | 1.55E-21 | 3.85E-21 | up |
| *Cdh18* | chr15:22689666 | Intron | 2.124509178 | 7.16E-20 | 1.69E-19 | up |
| *Lmbr1* | chr5:29296030 | Intron | 2.12127628 | 4.47E-23 | 1.17E-22 | up |
| *Poli* | chr18:70512603 | Intron | 2.107502944 | 1.17E-18 | 2.67E-18 | up |
| *Grik4* | chr9:42638656 | Intron | 2.100284957 | 6.19E-24 | 1.65E-23 | up |
| *Cdh20* | chr1:104977287 | Intron | 2.090724067 | 0.001369279 | 0.001677993 | up |
| *Psd3* | chr8:67797033 | Intron | 2.071046828 | 2.91E-40 | 1.14E-39 | up |
| *Zmynd8* | chr2:165792486 | Intron | 2.0605019 | 1.01E-30 | 3.33E-30 | up |
| *Bmi1* | chr2:18685455 | exon | 2.037925311 | 5.51E-31 | 1.83E-30 | up |
| *Fhit* | chr14:9633340 | Intron | 2.029183183 | 7.68E-07 | 1.12E-06 | up |
| *Cit* | chr5:115867071 | Intron | 2.010898882 | 2.98E-24 | 8.08E-24 | up |
| *A230006K03Rik* | chr7:61185163 | Intron | 1.999584304 | 3.85E-10 | 6.48E-10 | NC |
| *Suclg2* | chr6:95659191 | Intron | 1.997841791 | 5.30E-05 | 7.07E-05 | NC |
| *Bhlhb9* | chrX:135809927 | Intron | 1.997318161 | 4.55E-26 | 1.31E-25 | NC |
| *Rgl1* | chr1:152585260 | Intron | 1.992382419 | 6.87E-29 | 2.17E-28 | NC |
| *Gm38312* | chr1:89097574 | exon | 1.98940846 | 6.42E-13 | 1.21E-12 | NC |
| *Gigyf2* | chr1:87380383 | Intron | 1.989405603 | 2.59E-27 | 7.69E-27 | NC |
| *F2* | chr2:91628000 | Intron | 1.988759506 | 9.48E-06 | 1.32E-05 | NC |
| *Tmem192* | chr8:64952439 | Intron | 1.987603444 | 2.20E-27 | 6.56E-27 | NC |
| *Rb1* | chr14:73239231 | Intron | 1.971328773 | 1.73E-16 | 3.68E-16 | NC |
| *Parg* | chr14:32205426 | Intron | 1.967558053 | 0.002300507 | 0.002781508 | NC |
| *Aqp9* | chr9:71118394 | Intron | 1.967000956 | 0.000497876 | 0.000625368 | NC |
| *Mrgprx2* | chr7:48490339 | Intron | 1.966691677 | 0.001551783 | 0.00189395 | NC |
| *Elavl2* | chr4:91307686 | Intron | 1.96582306 | 2.13E-43 | 8.78E-43 | NC |
| *Trim14* | chr4:46505657 | Intron | 1.952782861 | 0.000928183 | 0.001145983 | NC |
| *Cpm* | chr10:117643974 | Intron | 1.950761713 | 7.65E-31 | 2.52E-30 | NC |
| *Ifi35* | chr11:101451511 | Intron | 1.946536502 | 3.89E-23 | 1.02E-22 | NC |
| *Stra6* | chr9:58128085 | Intron | 1.942651047 | 3.32E-08 | 5.19E-08 | NC |
| *Hk1* | chr10:62370757 | Intron | 1.941481075 | 0.000572057 | 0.000716556 | NC |
| *Os9* | chr10:127098282 | Intron | 1.940612412 | 1.61E-21 | 4.01E-21 | NC |
| *Epo* | chr5:137499488 | Intron | 1.931802959 | 0.055796061 | 0.062533047 | NC |
| *Adamts13* | chr2:26979958 | Intron | 1.921031777 | 0.000437449 | 0.000552535 | NC |
| *Lrrc69* | chr4:14677703 | Intron | 1.908373443 | 0.004055276 | 0.004835342 | NC |
| *Osbpl1a* | chr18:12935029 | Intron | 1.902229167 | 1.08E-10 | 1.86E-10 | NC |
| *Inpp4b* | chr8:81348492 | Intron | 1.900357048 | 4.29E-07 | 6.35E-07 | NC |
| *Sult2a2* | chr7:13758413 | Intron | 1.866835553 | 2.28E-09 | 3.73E-09 | NC |
| *Susd4* | chr1:182864557 | Intron | 1.864302802 | 2.87E-12 | 5.27E-12 | NC |
| *Cdh13* | chr8:118667779 | Intron | 1.859180347 | 2.68E-32 | 9.10E-32 | NC |
| *Celf2* | chr2:7404828 | Intron | 1.84811904 | 4.18E-15 | 8.55E-15 | NC |
| *Ino80* | chr2:119392345 | Intron | 1.847280496 | 4.80E-21 | 1.17E-20 | NC |
| *Obox1* | chr7:15462924 | Intron | 1.839136774 | 0.009959767 | 0.011668076 | NC |
| *Tmem132b* | chr5:125718411 | Intron | 1.836638084 | 5.84E-07 | 8.60E-07 | NC |
| *Cftr* | chr6:18256697 | Intron | 1.834494768 | 2.64E-29 | 8.46E-29 | NC |
| *Vstm2a* | chr11:16387865 | Intron | 1.832884517 | 8.15E-14 | 1.59E-13 | NC |
| *Polr1a* | chr6:71958501 | Intron | 1.832331367 | 0.002447199 | 0.002956896 | NC |
| *AC121788.3* | chr10:71563967 | Intron | 1.832133995 | 1.07E-07 | 1.64E-07 | NC |
| *Hormad1* | chr3:95578083 | CDS | 1.829111696 | 4.93E-15 | 1.00E-14 | NC |
| *Apobec1* | chr6:122586717 | Intron | 1.805910416 | 0.000400888 | 0.000508128 | NC |
| *Gm2309* | chrX:35132484 | Intron | 1.797402948 | 9.08E-07 | 1.32E-06 | NC |
| *Fto* | chr8:91390338 | Intron | 1.749767409 | 4.25E-31 | 1.42E-30 | NC |
| *Tenm4* | chr7:96197068 | Intron | 1.74814916 | 8.81E-20 | 2.07E-19 | NC |
| *Ints6l* | chrX:56459663 | Intron | 1.727362897 | 1.16E-14 | 2.32E-14 | NC |
| *AC160116.1* | chr9:35305457 | Intron | 1.706219327 | 4.77E-11 | 8.30E-11 | NC |
| *Me3* | chr7:89688833 | Intron | 1.705372587 | 5.50E-05 | 7.33E-05 | NC |
| *Adam22* | chr5:8268321 | Intron | 1.703041407 | 4.56E-12 | 8.33E-12 | NC |
| *Sulf1* | chr1:12693658 | Intron | 1.702582157 | 1.33E-08 | 2.11E-08 | NC |
| *Gm14308* | chr2:176625883 | Intron | 1.697229205 | 6.70E-06 | 9.41E-06 | NC |
| *Pkd1l1* | chr11:8842539 | Intron | 1.691791553 | 3.05E-06 | 4.34E-06 | NC |
| *Pdzd4* | chrX:73817088 | Intron | 1.662551692 | 2.49E-14 | 4.93E-14 | NC |
| *AC147634.1* | chr10:108438475 | Intron | 1.65549841 | 0.002200919 | 0.002668228 | NC |
| *1700022A22Rik* | chr15:46365246 | Intron | 1.649169254 | 9.87E-07 | 1.43E-06 | NC |
| *Capza1* | chr3:104860637 | Intron | 1.644592843 | 2.82E-16 | 5.96E-16 | NC |
| *Gm32850* | chr7:82160608 | Intron | 1.644409642 | 0.006158805 | 0.007285906 | NC |
| *Pir* | chrX:164285411 | Intron | 1.640748443 | 4.39E-24 | 1.18E-23 | NC |
| *Gm10040* | chr3:68736605 | Intron | 1.632467479 | 0.003681735 | 0.00439864 | NC |
| *Plch1* | chr3:63861735 | Intron | 1.630429428 | 0.003058057 | 0.003670479 | NC |
| *Nt5c2* | chr19:46890589 | Intron | 1.628905866 | 0.000435727 | 0.000550744 | NC |
| *4930586N03Rik* | chr13:23326501 | Intron | 1.626893058 | 4.21E-19 | 9.76E-19 | NC |
| *Als2* | chr1:59199703 | Intron | 1.626520433 | 9.89E-06 | 1.37E-05 | NC |
| *Gm3182* | chr14:4489224 | three_prime_utr | 1.625843904 | 0.010465194 | 0.012252264 | NC |
| *Sgce* | chr6:4738333 | Intron | 1.621976245 | 6.55E-18 | 1.45E-17 | NC |
| *Pofut1* | chr2:153244237 | Intron | 1.618982741 | 5.38E-05 | 7.18E-05 | NC |
| *2700049A03Rik* | chr12:71208983 | Intron | 1.618185136 | 8.65E-07 | 1.26E-06 | NC |
| *E330016L19Rik* | chrX:30041329 | Intron | 1.607866121 | 0.000809028 | 0.001002644 | NC |
| *Sulf2* | chr2:166145448 | Intron | 1.593853473 | 3.71E-25 | 1.04E-24 | NC |
| *Wdr7* | chr18:63860379 | Intron | 1.587392324 | 5.92E-13 | 1.11E-12 | NC |
| *Zmym6* | chr4:127122651 | CDS | 1.575038173 | 1.21E-12 | 2.26E-12 | NC |
| *Atp2c2* | chr8:119700971 | Intron | 1.55003234 | 1.91E-07 | 2.87E-07 | NC |
| *Prss32* | chr17:23851614 | Intron | 1.549606833 | 0.037264406 | 0.042367195 | NC |
| *Zfp69* | chr4:120939188 | Intron | 1.549294456 | 0.008666668 | 0.010195188 | NC |
| *Emcn* | chr3:137378020 | Intron | 1.548550579 | 0.008086082 | 0.009534728 | NC |
| *AC210924.1* | chr13:18643215 | Intron | 1.548499722 | 0.002867648 | 0.003453388 | NC |
| *Tns3* | chr11:8525205 | Intron | 1.533293352 | 2.32E-27 | 6.90E-27 | NC |
| *Rhoh* | chr5:65874641 | Intron | 1.523656711 | 2.93E-08 | 4.60E-08 | NC |
| *Phex* | chrX:157285182 | Intron | 1.51884593 | 1.74E-08 | 2.75E-08 | NC |
| *Maml2* | chr9:13697752 | Intron | 1.518349878 | 3.64E-18 | 8.13E-18 | NC |
| *Mapk1* | chr16:17030533 | Intron | 1.495418686 | 4.58E-22 | 1.16E-21 | NC |
| *Col6a6* | chr9:105733577 | Intron | 1.489171521 | 9.07E-21 | 2.19E-20 | NC |
| *Lama1* | chr17:67807628 | Intron | 1.459909604 | 0.000185176 | 0.000239921 | NC |
| *Carmil1* | chr13:24029366 | Intron | 1.450264607 | 0.005954386 | 0.007053297 | NC |
| *Znrd1as* | chr17:36963240 | Intron | 1.435378114 | 3.01E-09 | 4.91E-09 | NC |
| *Gid8* | chr2:180713102 | five_prime_utr | 1.432811584 | 2.38E-14 | 4.72E-14 | NC |
| *Arhgef4* | chr1:34735328 | Intron | 1.432181162 | 3.46E-13 | 6.56E-13 | NC |
| *Npsr1* | chr9:24150537 | Intron | 1.424920096 | 0.003505963 | 0.00419418 | NC |
| *Gm29502* | chr13:100924106 | Intron | 1.379387791 | 0.071877432 | 0.079983056 | NC |
| *Gm12790* | chr4:101978879 | Intron | 1.379293886 | 0.001606373 | 0.001959255 | NC |
| *Tacr3* | chr3:134909180 | Intron | 1.355252995 | 0.029503659 | 0.033777117 | NC |
| *Herpud2* | chr9:25128197 | Intron | 1.353805395 | 0.000338367 | 0.000431908 | NC |
| *Fam19a1* | chr6:96499436 | Intron | 1.347877839 | 9.83E-19 | 2.25E-18 | NC |
| *Mgat4a* | chr1:37482285 | Intron | 1.34668375 | 0.011928871 | 0.013929843 | NC |
| *Myo6* | chr9:80196698 | Intron | 1.338152452 | 1.61E-07 | 2.43E-07 | NC |
| *Col8a1* | chr16:57629833 | Intron | 1.333529911 | 0.002205027 | 0.002671418 | NC |
| *Pik3r3* | chr4:116278865 | Intron | 1.32579869 | 9.26E-05 | 0.000122199 | NC |
| *Peak1* | chr9:56234016 | Intron | 1.324275501 | 5.78E-05 | 7.70E-05 | NC |
| *Gm14406* | chr2:177572004 | Intron | 1.321025128 | 2.01E-11 | 3.54E-11 | NC |
| *Fam135a* | chr1:24077157 | Intron | 1.319284712 | 9.73E-12 | 1.74E-11 | NC |
| *9130409J20Rik* | chr11:66950293 | intergenic(10kb) | 1.309779711 | 0.000204454 | 0.000264708 | NC |
| *Gsap* | chr5:21238444 | Intron | 1.307728094 | 0.003000517 | 0.003606199 | NC |
| *Prkdc* | chr16:15650454 | Intron | 1.304489509 | 0.000182158 | 0.000236349 | NC |
| *Nufip2* | chr11:77705826 | Intron | 1.303504644 | 3.86E-11 | 6.74E-11 | NC |
| *Platr28* | chr7:107267460 | Intron | 1.29727824 | 0.049112964 | 0.05514545 | NC |
| *Gm28363* | chr1:117704986 | Intron | 1.287107258 | 0.011865363 | 0.013864626 | NC |
| *Borcs5* | chr6:134658195 | Intron | 1.280823223 | 1.22E-09 | 2.02E-09 | NC |
| *Akr1c14* | chr13:4074755 | Intron | 1.280436019 | 0.010514192 | 0.012301673 | NC |
| *Nsd1* | chr13:55279670 | Intron | 1.272105479 | 1.26E-12 | 2.33E-12 | NC |
| *Gm21738* | chr14:19417359 | CDS | 1.2708026 | 1.70E-16 | 3.64E-16 | NC |
| *Vps13a* | chr19:16758400 | Intron | 1.26835357 | 6.81E-06 | 9.54E-06 | NC |
| *Olfr907* | chr9:38504338 | intergenic(10kb) | 1.264479528 | 0.001833699 | 0.002232007 | NC |
| *5430401F13Rik* | chr6:131504626 | Intron | 1.262502219 | 0.082836848 | 0.09159114 | NC |
| *4930533K18Rik* | chr10:70882615 | Intron | 1.260683165 | 0.077187015 | 0.085553274 | NC |
| *Phkb* | chr8:85981285 | Intron | 1.257631766 | 0.000149686 | 0.000195195 | NC |
| *Zfp560* | chr9:20356624 | Intron | 1.233076728 | 0.000580478 | 0.000726601 | NC |
| *Urm1* | chr2:29834636 | Intron | 1.228170697 | 2.03E-07 | 3.04E-07 | NC |
| *Gm45030* | chr16:76493226 | Intron | 1.227107028 | 0.018519368 | 0.021391229 | NC |
| *Upp2* | chr2:58689985 | Intron | 1.225836176 | 9.00E-05 | 0.000118911 | NC |
| *Spg11* | chr2:122070714 | Intron | 1.221565264 | 0.003069716 | 0.003682031 | NC |
| *Birc6* | chr17:74612682 | Intron | 1.197935249 | 1.68E-11 | 2.99E-11 | NC |
| *Myof* | chr19:37969333 | Intron | 1.197159682 | 1.50E-13 | 2.89E-13 | NC |
| *Cbx1* | chr11:96790234 | Intron | 1.180261479 | 7.97E-10 | 1.33E-09 | NC |
| *Csrnp3* | chr2:65931680 | Intron | 1.170110318 | 8.45E-10 | 1.40E-09 | NC |
| *Tpo* | chr12:30089482 | Intron | 1.151801674 | 7.49E-06 | 1.04E-05 | NC |
| *Atxn7l1* | chr12:33232625 | Intron | 1.148151634 | 0.004276169 | 0.005088669 | NC |
| *Nkain3* | chr4:20422832 | Intron | 1.137246508 | 1.42E-07 | 2.16E-07 | NC |
| *E330010L02Rik* | chrX:25934731 | Intron | 1.125848738 | 0.002587343 | 0.00312206 | NC |
| *Top2b* | chr14:16369001 | Intron | 1.115935318 | 8.31E-07 | 1.21E-06 | NC |
| *Tsga10* | chr1:37775574 | Intron | 1.101702964 | 0.088607168 | 0.097792057 | NC |
| *Gm14697* | chrX:49352981 | Intron | 1.098661129 | 0.027442766 | 0.03151739 | NC |
| *Ankrd6* | chr4:32924396 | Intron | 1.096571627 | 3.79E-10 | 6.39E-10 | NC |
| *Kalrn* | chr16:34048119 | Intron | 1.096156802 | 1.55E-11 | 2.75E-11 | NC |
| *Crisp2* | chr17:40797692 | Intron | 1.081830164 | 0.072921646 | 0.080980126 | NC |
| *Spaca9* | chr2:28697478 | Intron | 1.070182066 | 0.047704682 | 0.053664061 | NC |
| *CT025551.1* | chr13:47668158 | exon | 1.059746249 | 1.29E-06 | 1.87E-06 | NC |
| *Immp2l* | chr12:41847928 | Intron | 1.058054648 | 2.68E-07 | 4.01E-07 | NC |
| *Gm14401* | chr2:177079404 | Intron | 1.031420483 | 0.001484356 | 0.001816555 | NC |
| *Gpsm1* | chr2:26336258 | Intron | 1.02370979 | 7.68E-07 | 1.12E-06 | NC |
| *Sox2ot* | chr3:34352081 | Intron | 1.011435651 | 7.35E-06 | 1.03E-05 | NC |
| *Crhr1* | chr11:104163192 | Intron | 1.010829211 | 7.74E-07 | 1.13E-06 | NC |
| *Ppp6r3* | chr19:3491841 | Intron | 1.000897578 | 0.008390969 | 0.009887796 | NC |
| *Gm14597* | chrX:53599874 | Intron | 1.000392896 | 1.41E-10 | 2.42E-10 | NC |
| *Brwd1* | chr16:96026899 | Intron | 0.993280244 | 3.00E-05 | 4.05E-05 | NC |
| *Cbl* | chr9:44218267 | Intron | 0.987009947 | 0.003928136 | 0.004686833 | NC |
| *Lrrc7* | chr3:158100848 | Intron | 0.983837974 | 0.11844067 | 0.128755323 | NC |
| *Slc6a6* | chr6:91693095 | Intron | 0.981299868 | 1.55E-06 | 2.23E-06 | NC |
| *Ank3* | chr10:69960422 | Intron | 0.974280288 | 7.11E-09 | 1.14E-08 | NC |
| *Rock1* | chr18:10156517 | Intron | 0.968983006 | 0.000679441 | 0.000846961 | NC |
| *Gulp1* | chr1:44726861 | Intron | 0.966962721 | 0.00032604 | 0.000416466 | NC |
| *Runx1* | chr16:92648943 | Intron | 0.959600494 | 1.15E-07 | 1.75E-07 | NC |
| *Dpyd* | chr3:118584797 | Intron | 0.940030936 | 2.73E-05 | 3.70E-05 | NC |
| *Yipf1* | chr4:107346956 | Intron | 0.939010899 | 0.00155162 | 0.00189395 | NC |
| *Nprl3* | chr11:32246291 | Intron | 0.931053979 | 4.89E-05 | 6.55E-05 | NC |
| *Gm12381* | chr4:38618527 | Intron | 0.918340468 | 0.009878943 | 0.011580886 | NC |
| *Cadm1* | chr9:47786877 | Intron | 0.896224406 | 0.000264445 | 0.000339706 | NC |
| *Epha3* | chr16:63650780 | Intron | 0.882448369 | 9.87E-07 | 1.43E-06 | NC |
| *Ccdc60* | chr5:116212867 | Intron | 0.871891953 | 3.29E-06 | 4.68E-06 | NC |
| *Galc* | chr12:98248529 | Intron | 0.854498344 | 0.115223618 | 0.12563539 | NC |
| *Veph1* | chr3:66231834 | Intron | 0.836098598 | 0.000467631 | 0.000588604 | NC |
| *Gcom1* | chr9:71491218 | Intron | 0.830000232 | 6.82E-06 | 9.55E-06 | NC |
| *Mov10* | chr3:104813212 | Intron | 0.828928151 | 0.043451324 | 0.049169382 | NC |
| *Rbm20* | chr19:53714775 | Intron | 0.825730124 | 2.99E-07 | 4.45E-07 | NC |
| *Ccdc160* | chrX:52795978 | Intron | 0.825337036 | 0.000116424 | 0.000152701 | NC |
| *Hspbap1* | chr16:35777311 | Intron | 0.819749859 | 0.000607456 | 0.00075932 | NC |
| *Mettl27* | chr5:134941086 | three_prime_utr | 0.817027042 | 0.000873667 | 0.001080148 | NC |
| *Stard6* | chr18:70487449 | Intron | 0.809275202 | 0.031833579 | 0.036371573 | NC |
| *St3gal3* | chr4:117964631 | Intron | 0.781611443 | 0.137408609 | 0.148572032 | NC |
| *Camk1d* | chr2:5379215 | Intron | 0.778386644 | 2.36E-08 | 3.72E-08 | NC |
| *Sgcd* | chr11:47498727 | Intron | 0.738951835 | 3.66E-06 | 5.20E-06 | NC |
| *Ralgps1* | chr2:33279889 | Intron | 0.732739144 | 0.000275466 | 0.000353613 | NC |
| *Pax2* | chr19:44748091 | intergenic(10kb) | 0.732208305 | 0.004164281 | 0.004962046 | NC |
| *Stat4* | chr1:52009160 | Intron | 0.722781929 | 0.000207708 | 0.000268728 | NC |
| *Tom1l1* | chr11:90682973 | Intron | 0.705835982 | 0.04908423 | 0.05514545 | NC |
| *Gbe1* | chr16:70544690 | Intron | 0.701717144 | 0.195414901 | 0.209787052 | NC |
| *CT030658.1* | chr12:39095380 | Intron | 0.682968424 | 0.0053839 | 0.006385884 | NC |
| *Fam13c* | chr10:70442781 | Intron | 0.681319188 | 0.016956641 | 0.019623735 | NC |
| *Plac1* | chrX:53189898 | Intron | 0.680127159 | 0.000254823 | 0.00032781 | NC |
| *Vrk2* | chr11:26557699 | Intron | 0.664836041 | 0.263642124 | 0.279714094 | NC |
| *Slc2a12* | chr10:22686314 | Intron | 0.660599438 | 0.111256179 | 0.121602467 | NC |
| *Fam184b* | chr5:45607121 | Intron | 0.6571362 | 0.001838157 | 0.00223593 | NC |
| *Abca14* | chr7:120298859 | Intron | 0.648843909 | 0.013131338 | 0.015265075 | NC |
| *Tmem132d* | chr5:127968647 | Intron | 0.635058154 | 0.000627307 | 0.000783052 | NC |
| *Btbd3* | chr2:138576843 | Intron | 0.633916501 | 0.117329224 | 0.127777314 | NC |
| *Dclk2* | chr3:86868206 | Intron | 0.632008913 | 0.001683466 | 0.002051902 | NC |
| *Mtmr7* | chr8:40571607 | Intron | 0.617764405 | 2.83E-05 | 3.83E-05 | NC |
| *Mctp1* | chr13:76475563 | Intron | 0.61664419 | 0.012205778 | 0.014234831 | NC |
| *Thsd7b* | chr1:129335455 | Intron | 0.60361745 | 3.81E-16 | 8.02E-16 | NC |
| *Rabgap1l* | chr1:160419592 | Intron | 0.593933015 | 0.149232102 | 0.160971457 | NC |
| *Vwc2* | chr11:11142917 | Intron | 0.588067928 | 0.059213093 | 0.066239616 | NC |
| *Gm30835* | chr5:7769830 | Intron | 0.58680928 | 0.214151432 | 0.229357451 | NC |
| *Kcnn2* | chr18:45393259 | Intron | 0.572296996 | 0.00474795 | 0.005642671 | NC |
| *Slc35f2* | chr9:53815383 | Intron | 0.566850626 | 0.006492734 | 0.00767092 | NC |
| *Zfp985* | chr4:147548084 | intergenic(10kb) | 0.56587837 | 0.099846711 | 0.109794986 | NC |
| *Gm44644* | chr7:60920446 | Intron | 0.552271549 | 0.195056124 | 0.209526162 | NC |
| *Poln* | chr5:34046587 | Intron | 0.550413675 | 0.026260671 | 0.030217301 | NC |
| *Slco2a1* | chr9:103087602 | exon | 0.542135402 | 0.006156409 | 0.007285906 | NC |
| *Gm12718* | chr4:103453784 | Intron | 0.532673422 | 0.035927841 | 0.040924728 | NC |
| *Adcy7* | chr8:88297601 | Intron | 0.532188439 | 0.085451495 | 0.094424423 | NC |
| *Lair1* | chr7:4060797 | Intron | 0.521520788 | 0.012979591 | 0.015108077 | NC |
| *Gm5535* | chr2:144155659 | Intron | 0.514928987 | 0.042225527 | 0.047857361 | NC |
| *Lsamp* | chr16:41578071 | Intron | 0.511841348 | 0.49695091 | 0.516349683 | NC |
| *Upf2* | chr2:5964009 | Intron | 0.509413798 | 0.386835136 | 0.406603715 | NC |
| *Stk32b* | chr5:37501297 | Intron | 0.507220995 | 0.026912976 | 0.030928563 | NC |
| *Dctn4* | chr18:60531466 | Intron | 0.503247979 | 0.223835232 | 0.239304058 | NC |
| *Gm7247* | chr14:51489862 | Intron | 0.500866204 | 0.087332291 | 0.096443835 | NC |
| *AA792892* | chr5:94373560 | Intron | 0.490091516 | 0.404080991 | 0.423746578 | NC |
| *Col14a1* | chr15:55398610 | Intron | 0.489512528 | 0.150920378 | 0.162695583 | NC |
| *Pds5b* | chr5:150685476 | Intron | 0.489400675 | 0.107981654 | 0.118166139 | NC |
| *Afg3l1* | chr8:123502504 | three_prime_utr | 0.483225851 | 0.040691138 | 0.04617615 | NC |
| *Ptprq* | chr10:107560107 | Intron | 0.476092528 | 0.121993082 | 0.132378585 | NC |
| *Sptbn4* | chr7:27427851 | Intron | 0.475045427 | 0.067069255 | 0.074796889 | NC |
| *Lgals9* | chr11:78979182 | Intron | 0.471581939 | 0.204515142 | 0.219296449 | NC |
| *Gm37240* | chr3:84766646 | Intron | 0.470088229 | 0.038761861 | 0.04404204 | NC |
| *Oma1* | chr4:103316065 | Intron | 0.467626203 | 0.015805879 | 0.018303673 | NC |
| *Kcnd2* | chr6:21439842 | Intron | 0.465299577 | 0.336681399 | 0.354710903 | NC |
| *4930405D11Rik* | chr11:90834088 | Intron | 0.461500973 | 0.146306636 | 0.158004183 | NC |
| *Pcdha6* | chr18:36984566 | Intron | 0.454731915 | 0.323701494 | 0.341632481 | NC |
| *Fam234b* | chr6:135209743 | Intron | 0.434754615 | 0.2746272 | 0.291198144 | NC |
| *Xpo4* | chr14:57585868 | CDS | 0.412504124 | 0.081319374 | 0.089968256 | NC |
| *Fhad1* | chr4:141970802 | Intron | 0.400030219 | 0.4291319 | 0.448976149 | NC |
| *Nrros* | chr16:32148832 | Intron | 0.377178018 | 0.412281507 | 0.432095847 | NC |
| *Dcps* | chr9:35133565 | Intron | 0.357572878 | 0.399781059 | 0.419480416 | NC |
| *H2-T24* | chr17:36011213 | Intron | 0.357038585 | 0.419474174 | 0.439125654 | NC |
| *Gm9750* | chr19:9188630 | Intron | 0.350672421 | 0.335332325 | 0.353495345 | NC |
| *AC102569.1* | chr12:15059581 | Intron | 0.347233517 | 0.09388837 | 0.103494488 | NC |
| *Erbb4* | chr1:68587507 | Intron | 0.336071022 | 0.214466335 | 0.22955888 | NC |
| *Cxadr* | chr16:78352812 | Intron | 0.335905492 | 0.200115945 | 0.214706497 | NC |
| *Gm26858* | chr17:32370052 | Intron | 0.333178925 | 0.14742029 | 0.159111941 | NC |
| *Sntb1* | chr15:55902368 | Intron | 0.319935163 | 0.046528735 | 0.05250437 | NC |
| *Gm14295* | chr2:176807173 | Intron | 0.299930858 | 0.417080609 | 0.436872628 | NC |
| *Lrmda* | chr14:22233144 | Intron | 0.290543381 | 0.2536286 | 0.269564161 | NC |
| *Sft2d1* | chr17:8333674 | Intron | 0.283344077 | 0.431248708 | 0.450930192 | NC |
| *Gm29683* | chr7:70194455 | Intron | 0.277651868 | 0.601510106 | 0.620714533 | NC |
| *A830082K12Rik* | chr13:78262944 | Intron | 0.265028122 | 0.363509773 | 0.382753164 | NC |
| *Ate1* | chr7:130444878 | Intron | 0.264215867 | 0.397559165 | 0.417391003 | NC |
| *Gm28807* | chr7:40461431 | Intron | 0.257187222 | 0.43265991 | 0.452144594 | NC |
| *Camk2d* | chr3:126637194 | Intron | 0.252130542 | 0.662132642 | 0.679784505 | NC |
| *Gria3* | chrX:41483968 | Intron | 0.233270998 | 0.716706547 | 0.73042728 | NC |
| *Rftn1* | chr17:50078032 | Intron | 0.23073896 | 0.664530384 | 0.681473085 | NC |
| *Gm13710* | chr2:84505946 | Intron | 0.219042563 | 0.459588877 | 0.479732334 | NC |
| *Acer3* | chr7:98242650 | Intron | 0.213239612 | 0.647348798 | 0.665739389 | NC |
| *Kdm1b* | chr13:47048625 | Intron | 0.207984687 | 0.465574668 | 0.485420593 | NC |
| *Gm33201* | chr6:99968263 | Intron | 0.192095382 | 0.608003858 | 0.62705811 | NC |
| *Rnf38* | chr4:44219658 | Intron | 0.180119613 | 0.484626034 | 0.504412376 | NC |
| *Ccnd3* | chr17:47514315 | Intron | 0.166874871 | 0.388900008 | 0.408536863 | NC |
| *Skint11* | chr4:114216078 | Intron | 0.16407981 | 0.764277384 | 0.776722103 | NC |
| *Rasef* | chr4:73753280 | Intron | 0.162158087 | 0.641636344 | 0.660239786 | NC |
| *Rtn4* | chr11:29712042 | Intron | 0.161687666 | 0.480629434 | 0.500540435 | NC |
| *4930593A02Rik* | chr3:58764582 | Intron | 0.140484955 | 0.552583111 | 0.571855592 | NC |
| *Dpf3* | chr12:83352045 | Intron | 0.135134701 | 0.78671528 | 0.797733701 | NC |
| *Spata16* | chr3:26699890 | Intron | 0.127427909 | 0.681456619 | 0.697250695 | NC |
| *Cpne8* | chr15:90639517 | Intron | 0.126046391 | 0.697775655 | 0.712337245 | NC |
| *Gm44618* | chr7:28658228 | Intron | 0.12446693 | 0.697389529 | 0.712337245 | NC |
| *Sfmbt1* | chr14:30750350 | Intron | 0.116098966 | 0.669098279 | 0.685380806 | NC |
| *Galk2* | chr2:125876438 | Intron | 0.110708225 | 0.73706967 | 0.750335266 | NC |
| *Slc4a4* | chr5:89208696 | Intron | 0.11037743 | 0.539362917 | 0.559453799 | NC |
| *9130019P16Rik* | chr6:54333886 | Intron | 0.107891135 | 0.585710441 | 0.604755218 | NC |
| *Lrrc4c* | chr2:96377675 | Intron | 0.107461337 | 0.63153322 | 0.650953946 | NC |
| *Rcor1* | chr12:111098226 | Intron | 0.104990629 | 0.729518585 | 0.743066201 | NC |
| *Sncaip* | chr18:52877495 | Intron | 0.103713873 | 0.816437531 | 0.82694568 | NC |
| *6030407O03Rik* | chr1:73720590 | Intron | 0.102691548 | 0.799092567 | 0.809830654 | NC |
| *Gm10800* | chr2:98666645 | CDS | 0.098417227 | 0.549272221 | 0.568754417 | NC |
| *Cyp4a30-ps* | chr4:115348966 | Intron | 0.095956143 | 0.874417309 | 0.882215903 | NC |
| *Caprin2* | chr6:148868698 | Intron | 0.084902973 | 0.855903877 | 0.865950821 | NC |
| *Slc15a2* | chr16:36768122 | Intron | 0.078600827 | 0.713342494 | 0.727408403 | NC |
| *Ptprg* | chr14:11899118 | Intron | 0.063382762 | 0.694576009 | 0.709871585 | NC |
| *Lhfpl1* | chrX:145316461 | Intron | 0.055546398 | 0.898902279 | 0.905909312 | NC |
| *Glra3* | chr8:55973080 | Intron | 0.050349982 | 0.863083395 | 0.8722395 | NC |
| *Gm5622* | chr14:51555687 | Intron | 0.044853525 | 0.859226097 | 0.868826388 | NC |
| *4932412D23Rik* | chr16:42815160 | Intron | 0.016080829 | 0.973488172 | 0.975104367 | NC |
| *Poglut1* | chr16:38538841 | Intron | 0.015836469 | 0.935419695 | 0.940616471 | NC |
| *Dgkb* | chr12:37886656 | Intron | 0.013930343 | 0.975743716 | 0.976283099 | NC |
| *Hmcn1* | chr1:150819877 | Intron | 0.013389548 | 0.946943737 | 0.951147705 | NC |
| *Anapc10* | chr8:79764229 | Intron | 0.003477299 | 0.988125173 | 0.988125173 | NC |
| *Gramd1b* | chr9:40493904 | Intron | -0.010352971 | 0.970718542 | 0.972868528 | NC |
| *Thsd7a* | chr6:12664893 | Intron | -0.010526794 | 0.974264841 | 0.975342568 | NC |
| *Mtm1* | chrX:71213335 | Intron | -0.012962752 | 0.953093884 | 0.956263819 | NC |
| *Clca3b* | chr3:144831342 | Intron | -0.018512934 | 0.965958415 | 0.9686342 | NC |
| *Capsl* | chr15:9440795 | Intron | -0.02187004 | 0.943696437 | 0.948412299 | NC |
| *Tmtc1* | chr6:148318379 | Intron | -0.022096996 | 0.94791498 | 0.951595183 | NC |
| *Specc1l* | chr10:75258786 | Intron | -0.023187123 | 0.919302062 | 0.924923142 | NC |
| *Eri3* | chr4:117617483 | Intron | -0.03493716 | 0.835178352 | 0.845454596 | NC |
| *Mtdh* | chr15:34084043 | Intron | -0.041394701 | 0.900385139 | 0.906898777 | NC |
| *Cdcp1* | chr9:123193883 | Intron | -0.044121408 | 0.863846529 | 0.872523559 | NC |
| *Gm10722* | chr9:3001294 | CDS | -0.045561305 | 0.909851789 | 0.915924215 | NC |
| *AC160029.3* | chr10:122509828 | Intron | -0.052746723 | 0.89771675 | 0.905218562 | NC |
| *Srp54a* | chr12:55096696 | Intron | -0.057832379 | 0.868638821 | 0.87687466 | NC |
| *Gm26870* | chr9:3000416 | Intron | -0.065792953 | 0.757400099 | 0.77016527 | NC |
| *Slc22a22* | chr15:57311304 | Intron | -0.091432332 | 0.674891916 | 0.690924417 | NC |
| *Fam168a* | chr7:100722727 | Intron | -0.109610505 | 0.634585158 | 0.65355018 | NC |
| *Tom1l2* | chr11:60330189 | Intron | -0.117457612 | 0.564668471 | 0.583694993 | NC |
| *Xylt1* | chr7:117410286 | Intron | -0.126571886 | 0.743349771 | 0.756303027 | NC |
| *Gm26902* | chr19:34476652 | Intron | -0.1450347 | 0.765634179 | 0.777664346 | NC |
| *CT010472.1* | chr13:47419232 | Intron | -0.150671185 | 0.685742821 | 0.701239834 | NC |
| *Abca1* | chr4:53057677 | Intron | -0.157471888 | 0.770975335 | 0.782650228 | NC |
| *Unc5d* | chr8:28865588 | Intron | -0.163937817 | 0.783299423 | 0.794715221 | NC |
| *Ddx46* | chr13:55673672 | Intron | -0.176411418 | 0.652316215 | 0.670466978 | NC |
| *Adamts19* | chr18:58921112 | Intron | -0.1841697 | 0.634774153 | 0.65355018 | NC |
| *Ext1* | chr15:53177273 | Intron | -0.189804086 | 0.320143063 | 0.338074063 | NC |
| *Tbcd* | chr11:121613140 | Intron | -0.19743733 | 0.704027808 | 0.718314731 | NC |
| *Plppr1* | chr4:49267845 | Intron | -0.197758058 | 0.561295412 | 0.580539826 | NC |
| *Zfp346* | chr13:55110009 | Intron | -0.206516272 | 0.469547985 | 0.489281435 | NC |
| *Mpp2* | chr11:102070984 | Intron | -0.211445992 | 0.659886581 | 0.67786306 | NC |
| *Rps6ka3* | chrX:159246791 | Intron | -0.223147314 | 0.665494691 | 0.682075533 | NC |
| *Gm26848* | chr17:6363377 | Intron | -0.255403423 | 0.112177166 | 0.122535106 | NC |
| *Vmn2r-ps26* | chr5:151527705 | Intron | -0.25688838 | 0.526399984 | 0.546321084 | NC |
| *St13* | chr15:81388814 | Intron | -0.262326316 | 0.584228285 | 0.603569176 | NC |
| *Ppp1r9a* | chr6:4974321 | Intron | -0.27439197 | 0.522127749 | 0.542198064 | NC |
| *Asxl3* | chr18:22472099 | Intron | -0.276221585 | 0.548342504 | 0.568116733 | NC |
| *Gm15614* | chr5:32545625 | Intron | -0.284893468 | 0.66447843 | 0.681473085 | NC |
| *AY036118* | chr17:39847380 | exon | -0.285182662 | 0.031850245 | 0.036371573 | NC |
| *Sdhaf3* | chr6:6976247 | Intron | -0.293869747 | 0.464430505 | 0.484506752 | NC |
| *Pum2* | chr12:8723288 | Intron | -0.297677489 | 0.189474336 | 0.203772162 | NC |
| *Macc1* | chr12:119348023 | Intron | -0.298347888 | 0.54530197 | 0.56529013 | NC |
| *Fam81a* | chr9:70119182 | Intron | -0.304044122 | 0.189101417 | 0.203492012 | NC |
| *Inpp5b* | chr4:124773956 | Intron | -0.309190349 | 0.112367947 | 0.122669472 | NC |
| *Sec22a* | chr16:35343922 | Intron | -0.310221487 | 0.453023347 | 0.473151909 | NC |
| *Gm42906* | chr5:151355595 | Intron | -0.312482843 | 0.133482767 | 0.144413514 | NC |
| *Plpp3* | chr4:105204040 | Intron | -0.32177241 | 0.061520953 | 0.068736373 | NC |
| *Srp54b* | chr12:55246820 | Intron | -0.327790671 | 0.48585546 | 0.505401369 | NC |
| *Pax5* | chr4:44563521 | Intron | -0.349865425 | 0.157895294 | 0.170113382 | NC |
| *Adamts12* | chr15:11144271 | Intron | -0.355685622 | 0.384521257 | 0.404406436 | NC |
| *Hnf4aos* | chr2:163523087 | Intron | -0.366795797 | 0.320135114 | 0.338074063 | NC |
| *Slc7a15* | chr12:8581450 | Intron | -0.383624886 | 0.123563587 | 0.134002452 | NC |
| *Nup93* | chr8:94240160 | Intron | -0.383722907 | 0.228543787 | 0.244193775 | NC |
| *Atp6ap1l* | chr13:90901579 | Intron | -0.383981199 | 0.21132893 | 0.226468539 | NC |
| *Sh3gl2* | chr4:85411866 | Intron | -0.395835086 | 0.115769153 | 0.126154224 | NC |
| *Map2k6* | chr11:110409757 | Intron | -0.405233739 | 0.018153126 | 0.020981583 | NC |
| *Wdr33* | chr18:31873709 | Intron | -0.40606546 | 0.0485431 | 0.054573298 | NC |
| *Pcsk2os2* | chr2:143760279 | Intron | -0.407725381 | 0.121183272 | 0.131578717 | NC |
| *Rgs3* | chr4:62575979 | Intron | -0.420116044 | 0.489549577 | 0.5089516 | NC |
| *Ube2o* | chr11:116569305 | Intron | -0.423376636 | 0.293841356 | 0.311207054 | NC |
| *Aff4* | chr11:53387750 | Intron | -0.425635914 | 0.191145738 | 0.205447616 | NC |
| *Mpv17* | chr5:31150957 | Intron | -0.427556874 | 0.092455489 | 0.101977108 | NC |
| *Myo10* | chr15:25767393 | Intron | -0.429484491 | 0.002632833 | 0.003174835 | NC |
| *Zbtb44* | chr9:31055225 | Intron | -0.433814329 | 0.069741914 | 0.077681763 | NC |
| *Gm26668* | chr11:102932195 | Intron | -0.436932049 | 0.237996148 | 0.253545043 | NC |
| *Fbxw4* | chr19:45650044 | Intron | -0.445244638 | 0.012171936 | 0.014204515 | NC |
| *Gm16070* | chr1:17685294 | Intron | -0.448737628 | 0.236410704 | 0.252152843 | NC |
| *Atf6* | chr1:170849879 | Intron | -0.450706355 | 0.131351853 | 0.142278189 | NC |
| *Gm13301* | chr4:42222389 | Intron | -0.451481638 | 0.256316457 | 0.272261025 | NC |
| *Mei4* | chr9:81908883 | Intron | -0.459682746 | 0.297714873 | 0.314940924 | NC |
| *Zfp934* | chr13:62509976 | Intron | -0.460426165 | 0.107877041 | 0.118123076 | NC |
| *Ctdsp2* | chr10:126984692 | Intron | -0.471433614 | 0.079213898 | 0.087746117 | NC |
| *Gm3383* | chr14:5699107 | Intron | -0.472660696 | 0.119885243 | 0.130247473 | NC |
| *Ctdspl2* | chr2:121982034 | Intron | -0.472824705 | 0.021172517 | 0.024440214 | NC |
| *Fgf12* | chr16:28175019 | Intron | -0.473177687 | 0.094900936 | 0.104483391 | NC |
| *Kif13b* | chr14:64677287 | Intron | -0.477748168 | 0.060830266 | 0.068006659 | NC |
| *Igf2bp2* | chr16:22150759 | Intron | -0.479180095 | 0.002192863 | 0.002660242 | NC |
| *Gm4788* | chr1:139727024 | Intron | -0.482355104 | 3.07E-179 | 3.78E-178 | NC |
| *Nlrp4c* | chr7:6079641 | Intron | -0.489243164 | 0.104254002 | 0.114363481 | NC |
| *Kif1b* | chr4:149193106 | Intron | -0.490802465 | 0.217352968 | 0.232511154 | NC |
| *9530052E02Rik* | chr8:11012859 | Intron | -0.49745327 | 0.332375622 | 0.350582678 | NC |
| *Herc2* | chr7:56080669 | Intron | -0.497563039 | 0.240565238 | 0.256131224 | NC |
| *Gm9725* | chr8:81027724 | Intron | -0.50319945 | 0.23370636 | 0.249415396 | NC |
| *Epm2a* | chr10:11405565 | Intron | -0.507888469 | 0.09431129 | 0.103897404 | NC |
| *Atpaf1* | chr4:115808416 | Intron | -0.511152213 | 0.075346499 | 0.083615673 | NC |
| *Ino80d* | chr1:63095343 | Intron | -0.513904791 | 0.290678693 | 0.308037725 | NC |
| *Eif4g3* | chr4:138042431 | Intron | -0.516374155 | 0.026391027 | 0.030348004 | NC |
| *Havcr1* | chr11:46764254 | Intron | -0.523558834 | 0.072517835 | 0.080624865 | NC |
| *Kif26b* | chr1:178853609 | Intron | -0.526581369 | 0.004967403 | 0.005899606 | NC |
| *Abhd10* | chr16:45736568 | Intron | -0.530226618 | 0.117909177 | 0.128331696 | NC |
| *Scn7a* | chr2:66745932 | Intron | -0.534048945 | 0.034007054 | 0.038761188 | NC |
| *Mecom* | chr3:30456323 | Intron | -0.534563908 | 0.04079641 | 0.046266606 | NC |
| *Bola1* | chr3:96204208 | Intron | -0.536792206 | 0.25689606 | 0.272716639 | NC |
| *4930455D15Rik* | chr18:32684733 | Intron | -0.541076815 | 0.017642272 | 0.020404161 | NC |
| *Ap1s3* | chr1:79656843 | Intron | -0.54622611 | 0.299361895 | 0.316498265 | NC |
| *AC158938.1* | chr3:34849480 | Intron | -0.557924439 | 0.047357542 | 0.053314824 | NC |
| *Disc1* | chr8:125147336 | Intron | -0.558104671 | 0.229490443 | 0.245060591 | NC |
| *Chit1* | chr1:134122480 | Intron | -0.56882251 | 1.97E-10 | 3.36E-10 | NC |
| *Anp32b* | chr4:46471253 | Intron | -0.575511773 | 0.072926854 | 0.080980126 | NC |
| *AC124134.1* | chr12:7802960 | Intron | -0.579767396 | 0.23771073 | 0.253390119 | NC |
| *Naaladl2* | chr3:23976955 | Intron | -0.59391045 | 0.007332252 | 0.008657127 | NC |
| *A530053G22Rik* | chr6:60314173 | Intron | -0.632276682 | 0.103096106 | 0.113230553 | NC |
| *Gm11168* | chr9:3003514 | gene | -0.64598773 | 0.11826126 | 0.128637548 | NC |
| *Tcerg1l* | chr7:138264056 | Intron | -0.648627462 | 0.043464647 | 0.049169382 | NC |
| *Sema6d* | chr2:124250424 | Intron | -0.650880084 | 0.130166976 | 0.141079177 | NC |
| *Tfdp2* | chr9:96278279 | Intron | -0.675383358 | 0.296836762 | 0.314195637 | NC |
| *Tshr* | chr12:91440270 | Intron | -0.6771016 | 0.096387844 | 0.106055926 | NC |
| *Senp7* | chr16:56060012 | Intron | -0.681698661 | 0.000792737 | 0.000983451 | NC |
| *Pgm1* | chr5:64118810 | Intron | -0.683294419 | 0.071896372 | 0.079983056 | NC |
| *Tenm3* | chr8:48314932 | Intron | -0.684048602 | 1.81E-05 | 2.46E-05 | NC |
| *Trdn* | chr10:33267283 | Intron | -0.689313805 | 0.013072921 | 0.015206933 | NC |
| *Eif3j1* | chr2:122032409 | Intron | -0.694244276 | 0.044904143 | 0.050734393 | NC |
| *Gm43470* | chr3:150596351 | Intron | -0.709930889 | 0.167310511 | 0.180149926 | NC |
| *Kcnj6* | chr16:94756936 | Intron | -0.7107887 | 1.40E-05 | 1.92E-05 | NC |
| *Fam171a1* | chr2:3126568 | Intron | -0.715636738 | 0.132103326 | 0.143006591 | NC |
| *5730522E02Rik* | chr11:25972020 | Intron | -0.716023521 | 0.003018834 | 0.003625806 | NC |
| *Mcc* | chr18:44738617 | Intron | -0.717031519 | 8.64E-05 | 0.00011418 | NC |
| *Gm43824* | chr3:128681846 | Intron | -0.718669511 | 0.036404061 | 0.041415054 | NC |
| *Gphn* | chr12:78351068 | Intron | -0.724603369 | 1.92E-06 | 2.75E-06 | NC |
| *Mme* | chr3:63293275 | Intron | -0.725381553 | 0.000528172 | 0.000662503 | NC |
| *Cdk15* | chr1:59348270 | Intron | -0.726528202 | 0.103331479 | 0.11342024 | NC |
| *Atm* | chr9:53531119 | Intron | -0.733207722 | 0.047364771 | 0.053314824 | NC |
| *Prcd* | chr11:116667648 | Intron | -0.737972972 | 0.106008862 | 0.116218075 | NC |
| *Ttbk2* | chr2:120799835 | Intron | -0.756165991 | 0.027558578 | 0.031630327 | NC |
| *Tbc1d23* | chr16:57204943 | Intron | -0.758035757 | 0.142514394 | 0.154000628 | NC |
| *Scn1a* | chr2:66307953 | Intron | -0.759139998 | 0.102810472 | 0.1129854 | NC |
| *Gpc4* | chrX:52155461 | Intron | -0.759357813 | 0.000176239 | 0.000228997 | NC |
| *Stxbp5l* | chr16:37120379 | Intron | -0.767079357 | 0.249582857 | 0.265420077 | NC |
| *Acnat1* | chr4:49464869 | Intron | -0.772842336 | 0.007691118 | 0.009074917 | NC |
| *Stk24* | chr14:121295753 | Intron | -0.773120818 | 0.068566878 | 0.076419981 | NC |
| *Gm4924* | chr10:82393584 | Intron | -0.78509771 | 2.85E-05 | 3.86E-05 | NC |
| *Mageb18* | chrX:92251912 | Intron | -0.786888432 | 0.000348661 | 0.00044442 | NC |
| *AC121569.3* | chr12:9424005 | Intron | -0.78803679 | 0.005910871 | 0.007006337 | NC |
| *Trps1* | chr15:50746485 | Intron | -0.788834566 | 0.365179017 | 0.384287221 | NC |
| *4930512M02Rik* | chr11:11604793 | Intron | -0.792858585 | 0.029193172 | 0.033442811 | NC |
| *Smarca2* | chr19:26725986 | Intron | -0.801792607 | 0.042796654 | 0.048474308 | NC |
| *Hykk* | chr9:54924648 | Intron | -0.813562609 | 0.001323344 | 0.0016228 | NC |
| *Srl* | chr16:4519661 | Intron | -0.817649724 | 0.062628516 | 0.069930668 | NC |
| *AC139752.1* | chr12:8123657 | Intron | -0.818932162 | 0.242569384 | 0.258113219 | NC |
| *Ppef1* | chrX:160657847 | Intron | -0.821104201 | 0.109297273 | 0.119533573 | NC |
| *Daam2* | chr17:49560248 | Intron | -0.836955224 | 0.076930533 | 0.08532124 | NC |
| *Rgs7* | chr1:175176233 | Intron | -0.839009086 | 0.057139837 | 0.063959867 | NC |
| *Gm20752* | chr3:159230365 | Intron | -0.842668267 | 0.033667376 | 0.038398205 | NC |
| *Ccdc58* | chr16:36081534 | Intron | -0.843372138 | 0.023898153 | 0.027533837 | NC |
| *4933413G19Rik* | chr6:128381518 | Intron | -0.843416902 | 0.080882277 | 0.089539401 | NC |
| *Fry* | chr5:150349602 | Intron | -0.84841034 | 1.53E-06 | 2.21E-06 | NC |
| *Tmem26* | chr10:68734449 | Intron | -0.85064217 | 0.029732375 | 0.034017445 | NC |
| *Dst* | chr1:34103612 | Intron | -0.852819179 | 7.08E-05 | 9.40E-05 | NC |
| *Ezh2* | chr6:47537406 | Intron | -0.853794359 | 0.002551971 | 0.003081433 | NC |
| *Chrm2* | chr6:36499051 | Intron | -0.856415824 | 0.014924277 | 0.017315988 | NC |
| *Mboat7* | chr7:3693517 | five_prime_utr | -0.862372166 | 0.046716042 | 0.052682888 | NC |
| *Cfap61* | chr2:145935413 | Intron | -0.869257008 | 0.000873616 | 0.001080148 | NC |
| *Tbl1x* | chrX:77520624 | Intron | -0.873351529 | 2.58E-06 | 3.69E-06 | NC |
| *Ccdc7b* | chr8:129119398 | Intron | -0.88284487 | 1.58E-05 | 2.17E-05 | NC |
| *Smoc1* | chr12:81177947 | Intron | -0.894340175 | 0.000425984 | 0.000538806 | NC |
| *Bcar3* | chr3:122468886 | Intron | -0.894786908 | 0.000161851 | 0.000210907 | NC |
| *BC024978* | chr7:27199327 | Intron | -0.895716844 | 0.010530092 | 0.012312316 | NC |
| *Dennd4c* | chr4:86757337 | Intron | -0.897357721 | 0.013444177 | 0.015618717 | NC |
| *Ptprd* | chr4:77578254 | Intron | -0.900878161 | 0.000102538 | 0.00013488 | NC |
| *Sbspon* | chr1:15867046 | Intron | -0.908916275 | 0.112772827 | 0.123037262 | NC |
| *Casz1* | chr4:148859682 | Intron | -0.911114057 | 0.000448803 | 0.000565691 | NC |
| *Gm10863* | chr15:79183499 | Intron | -0.914338352 | 4.63E-05 | 6.21E-05 | NC |
| *Nwd2* | chr5:63689763 | Intron | -0.927079963 | 0.064459191 | 0.071930416 | NC |
| *Nr2c2* | chr6:92121392 | Intron | -0.951930184 | 0.106412829 | 0.116590327 | NC |
| *Cfhr3* | chr1:139615177 | Intron | -0.953779725 | 1.80E-241 | 3.32E-240 | NC |
| *Itsn2* | chr12:4619077 | Intron | -0.961373222 | 0.000169604 | 0.000220535 | NC |
| *Gpatch2* | chr1:187239132 | Intron | -0.962817642 | 0.032605889 | 0.037211008 | NC |
| *Grm8* | chr6:27556429 | Intron | -0.967737988 | 0.002927085 | 0.003520282 | NC |
| *Eml1* | chr12:108451337 | Intron | -0.971108656 | 0.000407009 | 0.000515165 | NC |
| *Garem1* | chr18:21265817 | Intron | -0.976298497 | 0.00424229 | 0.005051674 | NC |
| *Utrn* | chr10:12610412 | Intron | -0.984771982 | 0.003693293 | 0.004409539 | NC |
| *Btbd9* | chr17:30297052 | Intron | -0.986280092 | 6.47E-05 | 8.59E-05 | NC |
| *Pak3* | chrX:143701920 | Intron | -0.990254225 | 0.027680278 | 0.031749876 | NC |
| *Nav1* | chr1:135544287 | Intron | -1.008902056 | 2.06E-07 | 3.08E-07 | NC |
| *Tmem231* | chr8:111917386 | Intron | -1.009709714 | 0.001040855 | 0.00128247 | NC |
| *Tmc4* | chr7:3674043 | Intron | -1.028048132 | 0.001878797 | 0.00228383 | NC |
| *Gm14164* | chr2:152347733 | Intron | -1.034676426 | 0.056920749 | 0.063754057 | NC |
| *Cidea* | chr18:67355639 | Intron | -1.038782065 | 0.046807293 | 0.052752927 | NC |
| *A630023A22Rik* | chr14:34082212 | Intron | -1.048629034 | 0.024893863 | 0.028662781 | NC |
| *Slc11a2* | chr15:100400324 | Intron | -1.04972054 | 0.002875714 | 0.003460799 | NC |
| *D030025E07Rik* | chr3:128140151 | Intron | -1.054015326 | 0.036019531 | 0.041003365 | NC |
| *Smarca1* | chrX:47861916 | Intron | -1.060841948 | 0.001759192 | 0.002142757 | NC |
| *Obox3* | chr7:15599928 | Intron | -1.071854105 | 0.015547589 | 0.018016092 | NC |
| *Auts2* | chr5:131750586 | Intron | -1.086323072 | 4.49E-12 | 8.20E-12 | NC |
| *Psma3* | chr12:70992502 | Intron | -1.100401971 | 0.039980412 | 0.045398084 | NC |
| *Nckap5* | chr1:126426605 | Intron | -1.107505873 | 2.84E-13 | 5.39E-13 | NC |
| *Blnk* | chr19:40956418 | Intron | -1.110603753 | 4.45E-07 | 6.58E-07 | NC |
| *Pcdhga7* | chr18:37746195 | Intron | -1.120074422 | 0.028676182 | 0.032871368 | NC |
| *Enox2* | chrX:49141734 | Intron | -1.12231925 | 0.000398589 | 0.000505568 | NC |
| *Itgb5* | chr16:33850321 | Intron | -1.126251867 | 0.000376682 | 0.000479461 | NC |
| *Trpm2* | chr10:77954832 | Intron | -1.128724488 | 0.00637288 | 0.007534235 | NC |
| *Cdh4* | chr2:179446210 | Intron | -1.13553403 | 0.000625869 | 0.000781797 | NC |
| *Mfsd7c* | chr12:85801463 | Intron | -1.145218146 | 0.015330075 | 0.017775423 | NC |
| *Fam184a* | chr10:53667894 | Intron | -1.162133048 | 0.045463911 | 0.051334797 | NC |
| *Galp* | chr7:6209298 | Intron | -1.16733133 | 1.83E-07 | 2.75E-07 | NC |
| *1500004A13Rik* | chr3:88791122 | Intron | -1.169542894 | 0.000226229 | 0.000291856 | NC |
| *Cdh12* | chr15:21263783 | Intron | -1.185003532 | 7.07E-06 | 9.90E-06 | NC |
| *Prkce* | chr17:86330717 | Intron | -1.186739408 | 5.67E-06 | 7.99E-06 | NC |
| *Ap3m1* | chr14:21037726 | Intron | -1.18707619 | 0.000731084 | 0.000909458 | NC |
| *Olfr1196* | chr2:88709546 | intergenic(10kb) | -1.192149182 | 0.009780288 | 0.011480104 | NC |
| *Malrd1* | chr2:15916047 | Intron | -1.193697342 | 0.000383883 | 0.000487598 | NC |
| *Kcnh7* | chr2:62826152 | Intron | -1.203984163 | 0.05231132 | 0.058663871 | NC |
| *Sgcz* | chr8:37854791 | Intron | -1.206173395 | 0.000463247 | 0.000583492 | NC |
| *Grk4* | chr5:34678637 | Intron | -1.206985899 | 0.023874288 | 0.027523861 | NC |
| *Rexo1* | chr10:80552092 | Intron | -1.221658207 | 1.17E-05 | 1.62E-05 | NC |
| *Brinp3* | chr1:146587381 | Intron | -1.226863933 | 4.59E-307 | 1.15E-305 | NC |
| *Gm44947* | chr7:93301381 | Intron | -1.227884828 | 1.48E-05 | 2.03E-05 | NC |
| *Gm15286* | chr13:111588433 | Intron | -1.228928847 | 0.01259208 | 0.014675895 | NC |
| *Mup6* | chr4:59969743 | Intron | -1.235558336 | 0.000494264 | 0.000621262 | NC |
| *Fam135b* | chr15:71607508 | Intron | -1.240207862 | 0.000854579 | 0.001057994 | NC |
| *Rhox2c* | chrX:37466278 | Intron | -1.240585148 | 0.001115649 | 0.001372757 | NC |
| *Csmd1* | chr8:15968764 | Intron | -1.241829099 | 2.28E-07 | 3.40E-07 | NC |
| *Scarb2* | chr5:92467701 | Intron | -1.254566767 | 2.13E-10 | 3.63E-10 | NC |
| *AC155922.2* | chr12:50788890 | Intron | -1.263937143 | 0.002774442 | 0.003343369 | NC |
| *Tcf12* | chr9:71970566 | Intron | -1.266472058 | 0.0129624 | 0.015097776 | NC |
| *Gm26704* | chr15:38917091 | Intron | -1.280060408 | 0.00027999 | 0.000358911 | NC |
| *Adgrg4* | chrX:56882109 | Intron | -1.283473853 | 0.000135746 | 0.000177658 | NC |
| *Arhgap24* | chr5:102603915 | Intron | -1.286247414 | 1.15E-05 | 1.60E-05 | NC |
| *Gm11973* | chr11:6491947 | Intron | -1.287517458 | 0.008474685 | 0.009979947 | NC |
| *Clmp* | chr9:40762842 | Intron | -1.304695602 | 6.22E-08 | 9.63E-08 | NC |
| *Kcnu1* | chr8:25874508 | Intron | -1.312915022 | 0.000164893 | 0.000214717 | NC |
| *Ptgr2* | chr12:84312313 | Intron | -1.318117421 | 8.43E-05 | 0.000111575 | NC |
| *Gm4117* | chr13:89830739 | exon | -1.328264787 | 0.000489562 | 0.000615779 | NC |
| *Cd47* | chr16:49897338 | Intron | -1.331774968 | 0.000290399 | 0.000371465 | NC |
| *Uso1* | chr5:92160458 | Intron | -1.338076228 | 8.59E-08 | 1.32E-07 | NC |
| *Tmem234* | chr4:129607761 | exon | -1.355544696 | 0.021304162 | 0.024576503 | NC |
| *Rpn2* | chr2:157319813 | exon | -1.364238576 | 0.00072693 | 0.000904913 | NC |
| *Vmn2r57* | chr7:41430128 | Intron | -1.365994422 | 9.55E-05 | 0.000125972 | NC |
| *Ophn1* | chrX:98746012 | Intron | -1.369760599 | 1.63E-05 | 2.22E-05 | NC |
| *Fbxw7* | chr3:84863611 | Intron | -1.369828395 | 2.64E-11 | 4.62E-11 | NC |
| *Pabpc4* | chr4:123268599 | Intron | -1.370274991 | 0.005245097 | 0.006225328 | NC |
| *Ambra1* | chr2:91743249 | Intron | -1.376553036 | 0.000257136 | 0.000330551 | NC |
| *Cdk5rap1* | chr2:154355058 | Intron | -1.377583175 | 0.001063476 | 0.00130945 | NC |
| *Chd2* | chr7:73509369 | Intron | -1.383758483 | 0.00022027 | 0.000284371 | NC |
| *Sgta* | chr10:81045411 | Intron | -1.384547782 | 0.000169078 | 0.000220008 | NC |
| *AC153950.2* | chr10:51947478 | Intron | -1.390620932 | 0.000570899 | 0.0007156 | NC |
| *Hnf1aos1* | chr5:114972666 | Intron | -1.391106069 | 0.001527863 | 0.001867273 | NC |
| *Gm26510* | chr17:70883428 | Intron | -1.391357806 | 1.77E-09 | 2.92E-09 | NC |
| *Cobl* | chr11:12436856 | Intron | -1.392046943 | 1.05E-05 | 1.45E-05 | NC |
| *Shroom4* | chrX:6570818 | Intron | -1.409474233 | 3.05E-06 | 4.34E-06 | NC |
| *Gm28055* | chr1:51715696 | Intron | -1.425350116 | 0.000809317 | 0.001002644 | NC |
| *Kbtbd12* | chr6:88567964 | Intron | -1.427012014 | 2.50E-07 | 3.73E-07 | NC |
| *4930554G24Rik* | chr11:14126309 | Intron | -1.435858152 | 1.69E-11 | 3.00E-11 | NC |
| *Gm42303* | chr4:76472500 | Intron | -1.438061115 | 0.000129612 | 0.000169752 | NC |
| *2610206C17Rik* | chr7:84714533 | Intron | -1.44507246 | 1.96E-05 | 2.67E-05 | NC |
| *Cdyl2* | chr8:116638839 | Intron | -1.445759712 | 1.13E-13 | 2.19E-13 | NC |
| *4932443I19Rik* | chr8:13713476 | Intron | -1.448188298 | 3.29E-05 | 4.43E-05 | NC |
| *CT010475.1* | chr13:82208091 | Intron | -1.452565494 | 1.62E-07 | 2.44E-07 | NC |
| *Tsacc* | chr3:88293264 | Intron | -1.45714281 | 5.95E-07 | 8.75E-07 | NC |
| *Zfp654* | chr16:64845397 | Intron | -1.458264571 | 1.33E-05 | 1.83E-05 | NC |
| *Rcbtb2* | chr14:73188058 | Intron | -1.46306793 | 2.91E-08 | 4.56E-08 | NC |
| *Gm20383* | chr6:81998161 | intergenic(10kb) | -1.470122934 | 0.009874412 | 0.011580886 | NC |
| *Pdzd2* | chr15:12397979 | Intron | -1.470190433 | 0.000107838 | 0.000141644 | NC |
| *Zbtb7c* | chr18:75888618 | Intron | -1.474441787 | 1.49E-13 | 2.86E-13 | NC |
| *Saysd1* | chr14:20136516 | Intron | -1.474502193 | 1.91E-11 | 3.38E-11 | NC |
| *Abhd2* | chr7:79340262 | Intron | -1.475506078 | 1.53E-07 | 2.32E-07 | NC |
| *Ptpn4* | chr1:119745250 | Intron | -1.481802466 | 0.000229359 | 0.000295473 | NC |
| *Skint6* | chr4:112962402 | Intron | -1.491022797 | 0.002222257 | 0.002690492 | NC |
| *Gipc2* | chr3:152164285 | Intron | -1.491086118 | 6.02E-07 | 8.85E-07 | NC |
| *B4galt1* | chr4:40838759 | Intron | -1.491782952 | 9.32E-10 | 1.54E-09 | NC |
| *Rap1a* | chr3:105748072 | Intron | -1.491794557 | 1.30E-05 | 1.79E-05 | NC |
| *Dner* | chr1:84377837 | Intron | -1.493196116 | 2.71E-07 | 4.04E-07 | NC |
| *Trmt11* | chr10:30553491 | Intron | -1.49726431 | 1.43E-07 | 2.18E-07 | NC |
| *Cd86* | chr16:36653650 | Intron | -1.502840908 | 0.001255392 | 0.00154156 | NC |
| *Capn11* | chr17:45646874 | Intron | -1.519883124 | 5.49E-06 | 7.74E-06 | NC |
| *Baz2b* | chr2:60175004 | Intron | -1.520897078 | 3.18E-19 | 7.40E-19 | NC |
| *AC110091.3* | chr9:120267766 | Intron | -1.536383624 | 0.008668726 | 0.010195188 | NC |
| *Plcb1* | chr2:134996642 | Intron | -1.537311407 | 6.01E-05 | 7.99E-05 | NC |
| *Zfp638* | chr6:83903828 | Intron | -1.544849408 | 0.003094509 | 0.003709312 | NC |
| *Gm14661* | chrX:60592054 | Intron | -1.548710423 | 0.000115797 | 0.000151989 | NC |
| *Thap4* | chr1:93710662 | Intron | -1.551365215 | 0.000287609 | 0.000368156 | NC |
| *Pick1* | chr15:79235106 | Intron | -1.564827289 | 4.44E-05 | 5.97E-05 | NC |
| *Myh15* | chr16:49073889 | Intron | -1.568697103 | 4.20E-07 | 6.22E-07 | NC |
| *Sdk2* | chr11:113804352 | Intron | -1.572194851 | 2.99E-10 | 5.06E-10 | NC |
| *Slc1a1* | chr19:28848595 | Intron | -1.575927083 | 0.000216536 | 0.00027975 | NC |
| *Ppp4r3a* | chr12:101045855 | Intron | -1.579318303 | 0.000380365 | 0.00048347 | NC |
| *Mroh5* | chr15:73823876 | Intron | -1.589576884 | 7.17E-07 | 1.05E-06 | NC |
| *Csnk1g3* | chr18:53889805 | Intron | -1.590855886 | 0.000281982 | 0.000361209 | NC |
| *Agbl4* | chr4:111180872 | Intron | -1.591683817 | 3.40E-19 | 7.91E-19 | NC |
| *Mob1b* | chr5:88735094 | Intron | -1.593093502 | 0.000227512 | 0.000293302 | NC |
| *Cnih4* | chr1:181163020 | Intron | -1.593850154 | 0.004467765 | 0.005313176 | NC |
| *AC155255.2* | chr12:32417479 | Intron | -1.596646906 | 0.000915807 | 0.001131474 | NC |
| *Slc4a1* | chr11:102354910 | Intron | -1.60356322 | 0.000701524 | 0.000873887 | NC |
| *Fndc3a* | chr14:72616227 | Intron | -1.611328275 | 1.05E-12 | 1.96E-12 | NC |
| *Gm26871* | chr3:118448170 | Intron | -1.620427117 | 0.000645684 | 0.000805436 | NC |
| *Rp2* | chrX:20402344 | Intron | -1.624676725 | 2.99E-09 | 4.89E-09 | NC |
| *Elf2* | chr3:51300761 | Intron | -1.626327042 | 1.33E-05 | 1.83E-05 | NC |
| *Ppp2r2b* | chr18:42738510 | Intron | -1.6307813 | 0.00044857 | 0.000565691 | NC |
| *Trrap* | chr5:144802560 | Intron | -1.634240047 | 0.000103527 | 0.000136082 | NC |
| *Grik2* | chr10:49226801 | Intron | -1.638427988 | 2.60E-08 | 4.08E-08 | NC |
| *AC055777.2* | chr10:77023557 | Intron | -1.641314977 | 3.97E-07 | 5.89E-07 | NC |
| *Appl2* | chr10:83606563 | Intron | -1.646513593 | 0.000208693 | 0.00026981 | NC |
| *Chst9* | chr18:15707992 | Intron | -1.655345499 | 0.000733853 | 0.000912276 | NC |
| *Hook1* | chr4:95982602 | Intron | -1.656958898 | 0.002103519 | 0.002553568 | NC |
| *Aatf* | chr11:84504736 | Intron | -1.658876481 | 3.24E-06 | 4.61E-06 | NC |
| *Epha6* | chr16:59702694 | Intron | -1.668795273 | 1.35E-11 | 2.42E-11 | NC |
| *Rab11fip3* | chr17:26038526 | Intron | -1.677738418 | 1.49E-05 | 2.04E-05 | NC |
| *4933406I18Rik* | chr7:114408551 | Intron | -1.679624842 | 0.008801222 | 0.010344293 | NC |
| *Cep128* | chr12:91222676 | Intron | -1.686663392 | 0.000340173 | 0.000433907 | NC |
| *Jarid2* | chr13:44742001 | Intron | -1.687114909 | 1.20E-13 | 2.32E-13 | NC |
| *Fblim1* | chr4:141580879 | Intron | -1.700818855 | 1.73E-07 | 2.60E-07 | NC |
| *Rnf157* | chr11:116390195 | Intron | -1.708688983 | 1.72E-06 | 2.48E-06 | NC |
| *Golga5* | chr12:102488696 | Intron | -1.720311945 | 1.85E-06 | 2.66E-06 | NC |
| *AC124776.1* | chr12:36283456 | Intron | -1.721526311 | 4.48E-06 | 6.33E-06 | NC |
| *Ube2r2* | chr4:41164649 | Intron | -1.732303169 | 1.66E-05 | 2.26E-05 | NC |
| *Ctnna2* | chr6:77702956 | Intron | -1.735712273 | 2.30E-09 | 3.76E-09 | NC |
| *Tenm1* | chrX:42634656 | Intron | -1.742011777 | 0.001515539 | 0.001853463 | NC |
| *Lmo3* | chr6:138508885 | Intron | -1.745654992 | 8.64E-06 | 1.20E-05 | NC |
| *Pola1* | chrX:93352242 | Intron | -1.758472788 | 2.21E-08 | 3.49E-08 | NC |
| *Zfp777* | chr6:48040488 | Intron | -1.761931064 | 6.76E-12 | 1.22E-11 | NC |
| *Mblac2* | chr13:81723353 | Intron | -1.780268461 | 3.00E-05 | 4.05E-05 | NC |
| *Spc24* | chr9:21755806 | three_prime_utr | -1.784459118 | 4.02E-11 | 7.01E-11 | NC |
| *Gm20426* | chr6:90211359 | Intron | -1.784947634 | 5.92E-10 | 9.88E-10 | NC |
| *Maob* | chrX:16789223 | Intron | -1.794637226 | 1.88E-05 | 2.55E-05 | NC |
| *Diaph2* | chrX:129852257 | Intron | -1.796894029 | 1.02E-06 | 1.48E-06 | NC |
| *Gm17767* | chr1:51562229 | Intron | -1.798011 | 4.59E-05 | 6.16E-05 | NC |
| *1700030N03Rik* | chr19:3173537 | Intron | -1.798727815 | 1.11E-11 | 1.99E-11 | NC |
| *Ndst4* | chr3:125635519 | Intron | -1.800496539 | 5.28E-08 | 8.22E-08 | NC |
| *Me1* | chr9:86694389 | Intron | -1.804216022 | 0.000144599 | 0.000188835 | NC |
| *Trim37* | chr11:87154285 | Intron | -1.807293497 | 3.57E-06 | 5.07E-06 | NC |
| *Col26a1* | chr5:136780207 | Intron | -1.81762011 | 4.67E-07 | 6.90E-07 | NC |
| *D5Ertd615e* | chr5:45189155 | Intron | -1.823920125 | 1.95E-06 | 2.79E-06 | NC |
| *AC156031.1* | chr9:41177593 | Intron | -1.829596389 | 1.63E-05 | 2.22E-05 | NC |
| *Dph5* | chr3:115909929 | Intron | -1.839124437 | 7.10E-07 | 1.04E-06 | NC |
| *Mkl1* | chr15:81025568 | Intron | -1.840694091 | 6.27E-06 | 8.81E-06 | NC |
| *Bbs9* | chr9:22722833 | Intron | -1.84463264 | 9.84E-08 | 1.51E-07 | NC |
| *Pld1* | chr3:28065794 | Intron | -1.848905755 | 5.70E-06 | 8.02E-06 | NC |
| *Thrb* | chr14:17762636 | Intron | -1.849270513 | 1.61E-05 | 2.20E-05 | NC |
| *Dennd5a* | chr7:109927844 | Intron | -1.855190625 | 3.98E-10 | 6.68E-10 | NC |
| *Slc2a3* | chr6:122763952 | Intron | -1.857370231 | 3.61E-21 | 8.89E-21 | NC |
| *Esco1* | chr18:10579826 | Intron | -1.870675959 | 0.000992348 | 0.001223535 | NC |
| *Rtn4ip1* | chr10:43925908 | Intron | -1.876303331 | 0.002094437 | 0.002544248 | NC |
| *Wdpcp* | chr11:21835833 | Intron | -1.878643657 | 8.93E-06 | 1.24E-05 | NC |
| *Gm16332* | chr1:139909526 | Intron | -1.885183285 | 5.52E-223 | 9.34E-222 | NC |
| *Csnk2a1* | chr2:152265833 | Intron | -1.886387937 | 1.24E-12 | 2.30E-12 | NC |
| *Fam131a* | chr16:20701039 | Intron | -1.88963597 | 5.91E-07 | 8.70E-07 | NC |
| *Gm12132* | chr11:40066775 | Intron | -1.893804539 | 1.34E-10 | 2.30E-10 | NC |
| *Enox1* | chr14:77301329 | Intron | -1.895573267 | 1.42E-21 | 3.54E-21 | NC |
| *Pdss2* | chr10:43224826 | Intron | -1.897934217 | 2.46E-16 | 5.22E-16 | NC |
| *Cytip* | chr2:58186691 | Intron | -1.901115035 | 0.009350732 | 0.010983015 | NC |
| *1700113H08Rik* | chr10:87069352 | Intron | -1.903300367 | 1.17E-06 | 1.70E-06 | NC |
| *Epha4* | chr1:77415423 | Intron | -1.923778076 | 6.86E-08 | 1.06E-07 | NC |
| *Hmg20a* | chr9:56449171 | Intron | -1.924356357 | 8.59E-05 | 0.000113698 | NC |
| *Ero1lb* | chr13:12603409 | Intron | -1.925570619 | 9.66E-08 | 1.48E-07 | NC |
| *Fancc* | chr13:63438315 | Intron | -1.954244141 | 5.29E-11 | 9.18E-11 | NC |
| *Ugt8a* | chr3:125903365 | Intron | -1.957497093 | 3.50E-05 | 4.71E-05 | NC |
| *Alg6* | chr4:99732249 | Intron | -1.962265049 | 6.65E-10 | 1.11E-09 | NC |
| *F8* | chrX:75198772 | Intron | -1.963996862 | 3.27E-08 | 5.12E-08 | NC |
| *AC159239.2* | chr13:44268423 | Intron | -1.968124499 | 0.000142075 | 0.000185672 | NC |
| *Fam227b* | chr2:125997335 | Intron | -1.976267798 | 5.73E-07 | 8.45E-07 | NC |
| *Ppig* | chr2:69726149 | Intron | -1.990400668 | 5.40E-07 | 7.96E-07 | NC |
| *Pde7b* | chr10:20647770 | Intron | -1.991135799 | 1.72E-18 | 3.89E-18 | NC |
| *Cpq* | chr15:33561963 | Intron | -1.993004024 | 1.89E-11 | 3.35E-11 | NC |
| *Cfap97* | chr8:46192118 | Intron | -2.000904701 | 2.74E-05 | 3.72E-05 | down |
| *5031439G07Rik* | chr15:84981291 | Intron | -2.001509799 | 4.45E-09 | 7.22E-09 | down |
| *Itgbl1* | chr14:123728530 | Intron | -2.002205219 | 3.10E-10 | 5.24E-10 | down |
| *Arhgef9* | chrX:95074220 | Intron | -2.009638652 | 1.17E-06 | 1.69E-06 | down |
| *Cd9* | chr6:125488438 | Intron | -2.020020912 | 2.29E-09 | 3.76E-09 | down |
| *Xirp2* | chr2:67519363 | Intron | -2.025077817 | 0.000102293 | 0.000134654 | down |
| *Srrm3* | chr5:135844276 | Intron | -2.029299906 | 7.47E-19 | 1.72E-18 | down |
| *Chrm3* | chr13:9902731 | Intron | -2.03059743 | 1.16E-18 | 2.65E-18 | down |
| *Ppp1r8* | chr4:132830935 | Intron | -2.035313243 | 2.43E-08 | 3.82E-08 | down |
| *Cnot1* | chr8:95735005 | Intron | -2.056003828 | 1.55E-07 | 2.34E-07 | down |
| *Gm30097* | chr3:82976639 | Intron | -2.058991972 | 2.40E-10 | 4.07E-10 | down |
| *Efcab3* | chr11:105105979 | Intron | -2.05958128 | 3.17E-07 | 4.71E-07 | down |
| *Chd6* | chr2:161057904 | Intron | -2.061848262 | 9.28E-07 | 1.35E-06 | down |
| *Gm44734* | chr7:73609686 | Intron | -2.070596442 | 5.53E-06 | 7.79E-06 | down |
| *Hacd1* | chr2:13973819 | Intron | -2.070894143 | 3.43E-08 | 5.37E-08 | down |
| *Trim2* | chr3:84219163 | Intron | -2.073351691 | 1.24E-07 | 1.89E-07 | down |
| *Ap2b1* | chr11:83376793 | Intron | -2.073708422 | 1.52E-15 | 3.13E-15 | down |
| *Plekha5* | chr6:140502774 | Intron | -2.075603116 | 0.000147402 | 0.000192356 | down |
| *Gm14798* | chrX:94067913 | Intron | -2.077480092 | 1.27E-09 | 2.10E-09 | down |
| *Mier3* | chr13:111697299 | Intron | -2.081304493 | 4.68E-06 | 6.62E-06 | down |
| *Gm3952* | chr8:128748696 | Intron | -2.084399529 | 9.86E-10 | 1.63E-09 | down |
| *Akr1e1* | chr13:4599309 | Intron | -2.086272762 | 0.003473425 | 0.004158002 | down |
| *Slc38a6* | chr12:73314067 | three_prime_utr | -2.087825573 | 3.57E-12 | 6.53E-12 | down |
| *Cd28* | chr1:60727901 | Intron | -2.091548212 | 5.14E-06 | 7.25E-06 | down |
| *Ddah1* | chr3:145863301 | Intron | -2.103565142 | 2.67E-05 | 3.63E-05 | down |
| *Snx13* | chr12:35135609 | Intron | -2.104763849 | 3.61E-06 | 5.13E-06 | down |
| *Exph5* | chr9:53327252 | Intron | -2.105049987 | 8.44E-09 | 1.35E-08 | down |
| *Zfp438* | chr18:5310157 | Intron | -2.105870698 | 3.11E-09 | 5.07E-09 | down |
| *Mcmdc2* | chr1:9924348 | Intron | -2.107636474 | 2.58E-11 | 4.53E-11 | down |
| *A830018L16Rik* | chr1:11624260 | Intron | -2.112982729 | 1.34E-25 | 3.83E-25 | down |
| *Npas3* | chr12:53438721 | Intron | -2.119644957 | 1.19E-15 | 2.47E-15 | down |
| *Tlr8* | chrX:167247786 | Intron | -2.120703854 | 1.22E-10 | 2.09E-10 | down |
| *Gm16036* | chr5:141600056 | Intron | -2.124140298 | 2.99E-05 | 4.05E-05 | down |
| *Cspp1* | chr1:10078412 | Intron | -2.126237579 | 0.000356915 | 0.000454621 | down |
| *Tspan5* | chr3:138808700 | Intron | -2.132159354 | 9.12E-10 | 1.51E-09 | down |
| *Vat1l* | chr8:114327334 | Intron | -2.135955657 | 1.52E-07 | 2.30E-07 | down |
| *Astn2* | chr4:65779704 | Intron | -2.138690712 | 1.29E-07 | 1.96E-07 | down |
| *Arhgap26* | chr18:38604469 | Intron | -2.139378592 | 2.12E-18 | 4.78E-18 | down |
| *Rev3l* | chr10:39767188 | Intron | -2.141445389 | 8.28E-08 | 1.28E-07 | down |
| *Pebp4* | chr14:69893667 | Intron | -2.14305604 | 3.90E-15 | 7.98E-15 | down |
| *Dtnb* | chr12:3583347 | Intron | -2.144359401 | 3.14E-07 | 4.67E-07 | down |
| *Cmss1* | chr16:57587388 | Intron | -2.148970115 | 2.41E-08 | 3.80E-08 | down |
| *Negr1* | chr3:156802587 | Intron | -2.159153961 | 5.51E-20 | 1.31E-19 | down |
| *Zc3hav1* | chr6:38306155 | three_prime_utr | -2.166395572 | 8.71E-09 | 1.39E-08 | down |
| *Arf2* | chr11:103976298 | Intron | -2.166709309 | 8.17E-06 | 1.14E-05 | down |
| *Xkr6* | chr14:63645405 | Intron | -2.177782035 | 8.06E-12 | 1.45E-11 | down |
| *Akt3* | chr1:177218596 | Intron | -2.184087181 | 4.28E-06 | 6.06E-06 | down |
| *Cntn6* | chr6:104618126 | Intron | -2.189329989 | 3.78E-06 | 5.36E-06 | down |
| *Cntnap2* | chr6:45484157 | Intron | -2.190385267 | 4.62E-19 | 1.07E-18 | down |
| *2700060E02Rik* | chr14:19813714 | Intron | -2.192680573 | 4.66E-07 | 6.89E-07 | down |
| *Arhgef26* | chr3:62360862 | Intron | -2.197712269 | 8.30E-10 | 1.38E-09 | down |
| *Adcy1* | chr11:7143448 | Intron | -2.20116282 | 3.89E-08 | 6.07E-08 | down |
| *Kansl1* | chr11:104453320 | Intron | -2.202378107 | 9.86E-08 | 1.51E-07 | down |
| *Megf9* | chr4:70474794 | Intron | -2.205794592 | 1.77E-06 | 2.55E-06 | down |
| *Synpo2* | chr3:123196578 | Intron | -2.20614898 | 4.94E-25 | 1.38E-24 | down |
| *Themis* | chr10:28707271 | Intron | -2.213581672 | 8.37E-08 | 1.29E-07 | down |
| *Mrc1* | chr2:14286690 | Intron | -2.248032559 | 1.41E-08 | 2.24E-08 | down |
| *Dmxl1* | chr18:49943693 | Intron | -2.260095649 | 2.38E-10 | 4.05E-10 | down |
| *Tbc1d32* | chr10:56036315 | Intron | -2.260299875 | 8.13E-09 | 1.30E-08 | down |
| *Meis2* | chr2:115962427 | Intron | -2.260433189 | 3.72E-16 | 7.84E-16 | down |
| *Rnf220* | chr4:117329492 | Intron | -2.260722817 | 1.30E-39 | 5.05E-39 | down |
| *AC126549.1* | chr13:114125988 | Intron | -2.26221236 | 7.23E-08 | 1.12E-07 | down |
| *Ranbp2* | chr10:58471171 | Intron | -2.262294378 | 3.75E-13 | 7.10E-13 | down |
| *Tek* | chr4:94843807 | Intron | -2.262451864 | 1.35E-08 | 2.14E-08 | down |
| *Ptpn2* | chr18:67689742 | Intron | -2.264363311 | 1.68E-12 | 3.10E-12 | down |
| *Rnd3* | chr2:51139763 | Intron | -2.270029176 | 2.51E-06 | 3.59E-06 | down |
| *Adck1* | chr12:88402516 | Intron | -2.275355256 | 6.43E-09 | 1.03E-08 | down |
| *Rbmx2* | chrX:48703456 | Intron | -2.28012392 | 1.82E-06 | 2.61E-06 | down |
| *Usp34* | chr11:23373220 | Intron | -2.281206516 | 9.71E-05 | 0.000128017 | down |
| *4930526L06Rik* | chr19:11204068 | Intron | -2.282535359 | 3.10E-24 | 8.36E-24 | down |
| *Tmcc3* | chr10:94365620 | Intron | -2.287019889 | 7.30E-08 | 1.13E-07 | down |
| *4732465J04Rik* | chr10:95752241 | Intron | -2.288074248 | 1.74E-06 | 2.50E-06 | down |
| *Lonp2* | chr8:86681427 | Intron | -2.289950974 | 1.13E-05 | 1.56E-05 | down |
| *Art3* | chr5:92355091 | Intron | -2.300185122 | 5.86E-08 | 9.09E-08 | down |
| *Triqk* | chr4:12957999 | Intron | -2.300990873 | 5.81E-05 | 7.74E-05 | down |
| *9330182L06Rik* | chr5:9341018 | Intron | -2.302684825 | 3.44E-08 | 5.37E-08 | down |
| *Gm6558* | chr3:31653615 | Intron | -2.30338639 | 1.90E-07 | 2.85E-07 | down |
| *Ddx11* | chr17:66661648 | intergenic(10kb) | -2.304307231 | 7.09E-05 | 9.40E-05 | down |
| *Dach2* | chrX:113353795 | Intron | -2.305264287 | 8.06E-15 | 1.63E-14 | down |
| *Map3k2* | chr18:32175597 | Intron | -2.310797756 | 1.36E-07 | 2.07E-07 | down |
| *Zfp423* | chr8:87913604 | Intron | -2.310805079 | 7.65E-09 | 1.23E-08 | down |
| *Ntm* | chr9:29278689 | Intron | -2.310956569 | 1.83E-12 | 3.36E-12 | down |
| *Fbn1* | chr2:125405154 | Intron | -2.310969856 | 1.86E-20 | 4.45E-20 | down |
| *Gm19026* | chr1:6433416 | Intron | -2.313140703 | 3.84E-14 | 7.55E-14 | down |
| *Mpv17l* | chr16:13910805 | Intron | -2.321821362 | 5.14E-06 | 7.25E-06 | down |
| *Tnik* | chr3:28515132 | Intron | -2.322653522 | 6.32E-09 | 1.02E-08 | down |
| *Ankrd17* | chr5:90304773 | Intron | -2.3234445 | 8.57E-12 | 1.54E-11 | down |
| *Hcfc2* | chr10:82703754 | Intron | -2.333883594 | 5.88E-10 | 9.82E-10 | down |
| *Gria4* | chr9:4482674 | Intron | -2.33424977 | 3.33E-10 | 5.62E-10 | down |
| *Gm14066* | chr2:139261717 | Intron | -2.339897446 | 1.40E-09 | 2.30E-09 | down |
| *Sil1* | chr18:35413196 | Intron | -2.344006655 | 2.33E-17 | 5.11E-17 | down |
| *Specc1* | chr11:62125909 | Intron | -2.349179505 | 3.31E-09 | 5.38E-09 | down |
| *Dcc* | chr18:71632032 | Intron | -2.350089074 | 1.37E-12 | 2.54E-12 | down |
| *Fastkd5* | chr2:130624493 | Intron | -2.35176835 | 1.06E-05 | 1.47E-05 | down |
| *Zcchc10* | chr11:53330146 | Intron | -2.352631276 | 2.80E-06 | 3.99E-06 | down |
| *Scap* | chr9:110342732 | Intron | -2.362781063 | 1.63E-08 | 2.58E-08 | down |
| *Ube2e2* | chr14:18775098 | Intron | -2.3699638 | 3.63E-09 | 5.90E-09 | down |
| *Gfod1* | chr13:43281629 | Intron | -2.370604685 | 8.16E-14 | 1.59E-13 | down |
| *Gm16675* | chr8:46679245 | Intron | -2.371060815 | 2.74E-14 | 5.42E-14 | down |
| *Fat3* | chr9:15929765 | Intron | -2.372605305 | 1.97E-10 | 3.37E-10 | down |
| *Fancl* | chr11:26465134 | Intron | -2.377559191 | 1.37E-05 | 1.89E-05 | down |
| *Creb5* | chr6:53403527 | Intron | -2.383685744 | 5.44E-14 | 1.06E-13 | down |
| *Fbxl7* | chr15:26651294 | Intron | -2.387523784 | 9.71E-15 | 1.95E-14 | down |
| *Zmat4* | chr8:23836580 | Intron | -2.387688882 | 4.98E-05 | 6.66E-05 | down |
| *Spag17* | chr3:99906131 | Intron | -2.4036243 | 6.26E-09 | 1.01E-08 | down |
| *Plxdc2* | chr2:16379710 | Intron | -2.40776898 | 1.23E-12 | 2.29E-12 | down |
| *Arhgef38* | chr3:133127674 | Intron | -2.409702875 | 4.61E-09 | 7.47E-09 | down |
| *Dcaf6* | chr1:165406867 | Intron | -2.410145752 | 0.003600731 | 0.004304705 | down |
| *Macrod2* | chr2:140542136 | Intron | -2.426413973 | 5.33E-29 | 1.69E-28 | down |
| *Gm12682* | chr4:99673809 | Intron | -2.428248688 | 1.39E-05 | 1.90E-05 | down |
| *Aco2* | chr15:81892765 | Intron | -2.437554799 | 1.18E-07 | 1.80E-07 | down |
| *Frmd4b* | chr6:97317226 | Intron | -2.44940469 | 3.75E-26 | 1.09E-25 | down |
| *Garnl3* | chr2:33076564 | Intron | -2.452793124 | 6.57E-17 | 1.42E-16 | down |
| *Barx2* | chr9:31892104 | Intron | -2.457712315 | 7.10E-07 | 1.04E-06 | down |
| *Iqgap2* | chr13:95660409 | Intron | -2.467047692 | 5.97E-21 | 1.45E-20 | down |
| *Luc7l2* | chr6:38582744 | Intron | -2.47503828 | 1.46E-07 | 2.21E-07 | down |
| *Gm26904* | chr17:46771291 | Intron | -2.480343451 | 1.93E-21 | 4.79E-21 | down |
| *Gm44206* | chr6:115828967 | Intron | -2.482970536 | 4.31E-14 | 8.46E-14 | down |
| *Scd3* | chr19:44220976 | Intron | -2.495484934 | 1.50E-07 | 2.27E-07 | down |
| *Foxj3* | chr4:119596103 | Intron | -2.502861038 | 9.04E-11 | 1.56E-10 | down |
| *Blm* | chr7:80472879 | Intron | -2.504371028 | 7.02E-12 | 1.27E-11 | down |
| *Zfhx3* | chr8:107968010 | Intron | -2.507482834 | 4.90E-10 | 8.21E-10 | down |
| *Egflam* | chr15:7277589 | Intron | -2.50927535 | 7.11E-10 | 1.19E-09 | down |
| *Pcdh9* | chr14:93021972 | Intron | -2.509693115 | 1.10E-27 | 3.34E-27 | down |
| *Slc20a2* | chr8:22555222 | Intron | -2.511156704 | 3.53E-07 | 5.25E-07 | down |
| *Gpr158* | chr2:21583682 | Intron | -2.524372895 | 2.45E-22 | 6.28E-22 | down |
| *Zfp277* | chr12:40343461 | Intron | -2.527613657 | 5.96E-09 | 9.62E-09 | down |
| *Scn2a* | chr2:65756059 | Intron | -2.531827393 | 4.00E-14 | 7.86E-14 | down |
| *Diaph1* | chr18:37908960 | Intron | -2.537847621 | 2.74E-16 | 5.80E-16 | down |
| *CT025587.1* | chr9:59215695 | Intron | -2.54319197 | 5.04E-08 | 7.86E-08 | down |
| *Itih5* | chr2:10180922 | Intron | -2.545787673 | 6.92E-11 | 1.20E-10 | down |
| *Usp13* | chr3:32880361 | Intron | -2.54782141 | 2.27E-11 | 4.00E-11 | down |
| *AC107755.1* | chr9:67201093 | Intron | -2.562819353 | 5.07E-09 | 8.21E-09 | down |
| *Ica1* | chr6:8700198 | Intron | -2.562941278 | 6.24E-11 | 1.08E-10 | down |
| *Arhgap35* | chr7:16528696 | Intron | -2.569295877 | 5.64E-12 | 1.03E-11 | down |
| *Kcnma1* | chr14:23412233 | Intron | -2.572484307 | 1.49E-25 | 4.26E-25 | down |
| *Rasal2* | chr1:157202565 | Intron | -2.58008617 | 4.69E-10 | 7.86E-10 | down |
| *Tmem184b* | chr15:79386971 | Intron | -2.580946186 | 1.02E-10 | 1.76E-10 | down |
| *Cacna2d3* | chr14:29344582 | Intron | -2.58779052 | 1.14E-08 | 1.82E-08 | down |
| *Epc2* | chr2:49467951 | Intron | -2.589621816 | 7.31E-08 | 1.13E-07 | down |
| *Zpbp* | chr11:11288944 | Intron | -2.595414036 | 5.77E-12 | 1.05E-11 | down |
| *Agtr1a* | chr13:30374199 | Intron | -2.598726258 | 1.08E-07 | 1.65E-07 | down |
| *Gm45455* | chr8:50244493 | Intron | -2.599877907 | 1.92E-11 | 3.39E-11 | down |
| *Zbed4* | chr15:88753755 | Intron | -2.607450081 | 3.73E-18 | 8.33E-18 | down |
| *Gm14565* | chrX:38717627 | Intron | -2.607511738 | 5.41E-09 | 8.74E-09 | down |
| *Nf2* | chr11:4839532 | Intron | -2.61104658 | 2.71E-06 | 3.87E-06 | down |
| *Rtn3* | chr19:7481042 | Intron | -2.619232673 | 1.39E-18 | 3.16E-18 | down |
| *Fgfr2* | chr7:130349181 | Intron | -2.62586222 | 1.98E-16 | 4.20E-16 | down |
| *Atf7* | chr15:102579740 | Intron | -2.629258249 | 2.48E-10 | 4.20E-10 | down |
| *Ola1* | chr2:73153515 | Intron | -2.63271392 | 1.91E-09 | 3.13E-09 | down |
| *Plpp4* | chr7:129350929 | Intron | -2.633547364 | 3.25E-10 | 5.49E-10 | down |
| *Cyth3* | chr5:143685530 | Intron | -2.64720599 | 1.48E-21 | 3.69E-21 | down |
| *Sorbs2* | chr8:45618396 | Intron | -2.650223906 | 8.91E-15 | 1.80E-14 | down |
| *2610307P16Rik* | chr13:28765585 | Intron | -2.652668374 | 5.18E-11 | 9.01E-11 | down |
| *Sec23b* | chr2:144573213 | Intron | -2.652912898 | 5.09E-10 | 8.52E-10 | down |
| *Gpm6b* | chrX:166266592 | Intron | -2.657834637 | 1.45E-12 | 2.68E-12 | down |
| *1700061G19Rik* | chr17:56878831 | Intron | -2.657889209 | 1.09E-14 | 2.19E-14 | down |
| *Gm15345* | chr18:56770944 | Intron | -2.658742957 | 4.99E-05 | 6.67E-05 | down |
| *Sirt5* | chr13:43385834 | CDS | -2.663382674 | 1.63E-07 | 2.46E-07 | down |
| *Pter* | chr2:12944710 | Intron | -2.666451147 | 2.52E-08 | 3.97E-08 | down |
| *Vwa8* | chr14:78997787 | Intron | -2.671943037 | 1.12E-05 | 1.54E-05 | down |
| *Zfp385b* | chr2:77604035 | Intron | -2.682921169 | 1.15E-21 | 2.87E-21 | down |
| *Dcaf17* | chr2:71068774 | Intron | -2.684638218 | 1.79E-10 | 3.06E-10 | down |
| *Cngb3* | chr4:19388512 | Intron | -2.695278403 | 1.23E-14 | 2.47E-14 | down |
| *Cfap43* | chr19:47806705 | Intron | -2.695926852 | 4.08E-15 | 8.35E-15 | down |
| *Mrpl16* | chr19:11770546 | Intron | -2.70637247 | 1.19E-07 | 1.81E-07 | down |
| *Cast* | chr13:74707740 | Intron | -2.715030541 | 3.09E-27 | 9.16E-27 | down |
| *Gm26883* | chr2:169786010 | Intron | -2.719200932 | 1.84E-20 | 4.43E-20 | down |
| *Myo1f* | chr17:33586628 | Intron | -2.723238202 | 4.88E-10 | 8.18E-10 | down |
| *Pde1c* | chr6:56297776 | Intron | -2.732077754 | 3.90E-22 | 9.94E-22 | down |
| *Cntnap4* | chr8:112796706 | Intron | -2.737561338 | 1.91E-08 | 3.02E-08 | down |
| *Megf11* | chr9:64707475 | three_prime_utr | -2.747348339 | 1.46E-05 | 2.01E-05 | down |
| *Thoc2* | chrX:41888956 | Intron | -2.749928882 | 5.40E-21 | 1.32E-20 | down |
| *Gm28694* | chr1:156986988 | Intron | -2.780903654 | 3.94E-29 | 1.26E-28 | down |
| *Zyg11b* | chr4:108288118 | Intron | -2.785497368 | 3.57E-15 | 7.31E-15 | down |
| *AC160401.1* | chr10:57141726 | Intron | -2.788861012 | 9.08E-09 | 1.45E-08 | down |
| *Lekr1* | chr3:65777875 | Intron | -2.788955083 | 3.54E-22 | 9.03E-22 | down |
| *Adam5* | chr8:24794103 | Intron | -2.791281106 | 1.12E-12 | 2.09E-12 | down |
| *Baalc* | chr15:38947810 | Intron | -2.795501893 | 4.56E-31 | 1.52E-30 | down |
| *Kdm6a* | chrX:18269287 | Intron | -2.795919085 | 1.75E-11 | 3.11E-11 | down |
| *C130071C03Rik* | chr13:83814482 | Intron | -2.800117013 | 1.89E-13 | 3.62E-13 | down |
| *Zdhhc14* | chr17:5683216 | Intron | -2.806215674 | 1.99E-07 | 2.99E-07 | down |
| *Efcab11* | chr12:99760506 | Intron | -2.808054244 | 4.42E-16 | 9.28E-16 | down |
| *Dzip1l* | chr9:99638236 | Intron | -2.811766542 | 9.27E-16 | 1.92E-15 | down |
| *Boll* | chr1:55272581 | Intron | -2.815533355 | 1.71E-24 | 4.69E-24 | down |
| *Qser1* | chr2:104771074 | Intron | -2.81971895 | 1.31E-16 | 2.80E-16 | down |
| *Atrnl1* | chr19:57830183 | Intron | -2.827221503 | 2.20E-25 | 6.24E-25 | down |
| *Lrrc27* | chr7:139220957 | exon | -2.830239639 | 1.84E-13 | 3.52E-13 | down |
| *Mbp* | chr18:82503162 | Intron | -2.830482307 | 4.41E-11 | 7.68E-11 | down |
| *A830035A12Rik* | chr11:107533363 | Intron | -2.835742278 | 2.03E-10 | 3.45E-10 | down |
| *Nelfa* | chr5:33909802 | Intron | -2.83696752 | 3.97E-10 | 6.68E-10 | down |
| *Kcnj3* | chr2:55492778 | Intron | -2.837932399 | 2.83E-17 | 6.18E-17 | down |
| *Zbtb20* | chr16:43289454 | Intron | -2.838802118 | 4.46E-29 | 1.42E-28 | down |
| *2010111I01Rik* | chr13:63159634 | Intron | -2.840338587 | 8.77E-16 | 1.82E-15 | down |
| *Nrcam* | chr12:44485731 | Intron | -2.840516554 | 2.13E-18 | 4.79E-18 | down |
| *Hdgfl2* | chr17:56091776 | Intron | -2.846823254 | 3.71E-10 | 6.27E-10 | down |
| *Stk40* | chr4:126122959 | Intron | -2.84755337 | 4.27E-15 | 8.71E-15 | down |
| *Nsd2* | chr5:33858372 | Intron | -2.862865745 | 1.01E-06 | 1.46E-06 | down |
| *Cubn* | chr2:13409265 | Intron | -2.865560273 | 1.14E-08 | 1.82E-08 | down |
| *Gm16157* | chr7:68275580 | Intron | -2.86947465 | 9.96E-08 | 1.53E-07 | down |
| *Plekhm3* | chr1:64809216 | Intron | -2.870990293 | 8.48E-08 | 1.30E-07 | down |
| *4930570N18Rik* | chr1:193210126 | Intron | -2.882469616 | 5.28E-08 | 8.22E-08 | down |
| *Nsdhl* | chrX:72946053 | Intron | -2.88548371 | 1.13E-10 | 1.94E-10 | down |
| *Fam155a* | chr8:9356254 | Intron | -2.887629731 | 4.97E-29 | 1.58E-28 | down |
| *4930544I03Rik* | chr12:90967647 | Intron | -2.898255665 | 2.77E-13 | 5.26E-13 | down |
| *D630045J12Rik* | chr6:38222713 | Intron | -2.901764602 | 4.61E-18 | 1.03E-17 | down |
| *Plag1* | chr4:3910978 | Intron | -2.905877502 | 5.82E-13 | 1.10E-12 | down |
| *Pdia5* | chr16:35447429 | Intron | -2.906866096 | 3.40E-13 | 6.45E-13 | down |
| *Sh3gl3* | chr7:82215750 | Intron | -2.913010285 | 1.42E-08 | 2.25E-08 | down |
| *Sytl3* | chr17:6680057 | exon | -2.917611177 | 2.19E-17 | 4.81E-17 | down |
| *Col9a1* | chr1:24188348 | Intron | -2.918226752 | 1.06E-16 | 2.28E-16 | down |
| *Nfkb1* | chr3:135682470 | Intron | -2.920511447 | 7.10E-06 | 9.93E-06 | down |
| *Gm4804* | chr12:15627735 | Intron | -2.921388276 | 1.60E-11 | 2.85E-11 | down |
| *Ttc39b* | chr4:83244342 | Intron | -2.923363712 | 1.98E-14 | 3.93E-14 | down |
| *Wdr89* | chr12:75642415 | Intron | -2.929190256 | 6.26E-15 | 1.27E-14 | down |
| *Gm12068* | chr11:24594367 | Intron | -2.933132233 | 5.09E-12 | 9.27E-12 | down |
| *Rap1gap2* | chr11:74542306 | Intron | -2.936838708 | 5.83E-16 | 1.22E-15 | down |
| *Trak2* | chr1:58942183 | Intron | -2.939038422 | 1.88E-09 | 3.08E-09 | down |
| *Arl15* | chr13:113927550 | Intron | -2.939735652 | 1.18E-22 | 3.05E-22 | down |
| *Csmd3* | chr15:47964288 | Intron | -2.945123743 | 2.05E-38 | 7.76E-38 | down |
| *Gabra3* | chrX:72573843 | Intron | -2.94602456 | 1.61E-10 | 2.75E-10 | down |
| *Abhd6* | chr14:8050354 | Intron | -2.974029482 | 1.56E-24 | 4.29E-24 | down |
| *Gm5087* | chr14:13246251 | Intron | -2.982703768 | 4.68E-22 | 1.19E-21 | down |
| *R3hdm2* | chr10:127465384 | Intron | -2.98513807 | 2.14E-13 | 4.09E-13 | down |
| *Zcchc16* | chrX:144908923 | Intron | -2.990244178 | 9.01E-15 | 1.81E-14 | down |
| *Bckdhb* | chr9:83946750 | Intron | -2.997864136 | 1.15E-32 | 3.97E-32 | down |
| *AU023762* | chr9:113504617 | Intron | -2.999250183 | 7.00E-20 | 1.65E-19 | down |
| *Pdzrn4* | chr15:92405604 | Intron | -3.008234521 | 2.24E-11 | 3.93E-11 | down |
| *2010308F09Rik* | chrX:13243578 | Intron | -3.012628547 | 1.89E-11 | 3.34E-11 | down |
| *Gm3143* | chr3:34704446 | Intron | -3.013543322 | 1.33E-12 | 2.47E-12 | down |
| *Grm5* | chr7:87905374 | Intron | -3.030301175 | 5.68E-12 | 1.03E-11 | down |
| *Tfb1m* | chr17:3521920 | Intron | -3.036328763 | 5.75E-08 | 8.93E-08 | down |
| *Tbl1xr1* | chr3:22185123 | Intron | -3.037715589 | 6.26E-11 | 1.08E-10 | down |
| *Arhgef39* | chr4:43496163 | Intron | -3.038643889 | 1.52E-16 | 3.25E-16 | down |
| *Gm13377* | chr2:21078736 | Intron | -3.040969524 | 1.37E-09 | 2.26E-09 | down |
| *Ajuba* | chr14:54570731 | Intron | -3.044161645 | 1.61E-05 | 2.20E-05 | down |
| *Psat1* | chr19:15921423 | Intron | -3.051640626 | 2.76E-13 | 5.25E-13 | down |
| *Plcxd3* | chr15:4415232 | Intron | -3.061176142 | 1.26E-16 | 2.70E-16 | down |
| *Vwc2l* | chr1:70756471 | Intron | -3.064802969 | 5.15E-14 | 1.01E-13 | down |
| *Zfp442* | chr2:150442883 | Intron | -3.064844236 | 1.00E-28 | 3.14E-28 | down |
| *Rtn1* | chr12:72345130 | Intron | -3.070488003 | 5.67E-13 | 1.07E-12 | down |
| *Cbx5* | chr15:103230943 | Intron | -3.073924258 | 5.06E-09 | 8.19E-09 | down |
| *Phf21a* | chr2:92361348 | three_prime_utr | -3.074520296 | 8.39E-14 | 1.63E-13 | down |
| *Lvrn* | chr18:46892431 | Intron | -3.08378916 | 9.95E-11 | 1.72E-10 | down |
| *Stpg2* | chr3:139369079 | Intron | -3.08433395 | 4.10E-14 | 8.06E-14 | down |
| *4930513D17Rik* | chr5:39579284 | Intron | -3.085545733 | 1.41E-12 | 2.61E-12 | down |
| *Glcci1* | chr6:8530214 | Intron | -3.086314213 | 4.30E-21 | 1.05E-20 | down |
| *Otud7a* | chr7:63498991 | Intron | -3.086779287 | 1.11E-16 | 2.38E-16 | down |
| *Glis1* | chr4:107510439 | Intron | -3.088999907 | 8.73E-12 | 1.57E-11 | down |
| *Rab27a* | chr9:73062981 | Intron | -3.089314962 | 1.47E-22 | 3.80E-22 | down |
| *Rhobtb1* | chr10:69233923 | Intron | -3.094562611 | 5.13E-21 | 1.25E-20 | down |
| *Lamb1* | chr12:31307468 | Intron | -3.097922184 | 8.72E-12 | 1.57E-11 | down |
| *Mtcl1* | chr17:66444676 | Intron | -3.103629804 | 3.12E-25 | 8.78E-25 | down |
| *Slk* | chr19:47640587 | Intron | -3.104361293 | 4.43E-15 | 9.02E-15 | down |
| *Zfp354c* | chr11:50822752 | Intron | -3.115771577 | 8.55E-12 | 1.54E-11 | down |
| *Cmc1* | chr9:118121573 | Intron | -3.133535493 | 7.21E-09 | 1.16E-08 | down |
| *Adamts2* | chr11:50793289 | Intron | -3.155406274 | 2.65E-28 | 8.26E-28 | down |
| *Cntn5* | chr9:10706759 | Intron | -3.157811614 | 3.07E-10 | 5.20E-10 | down |
| *Wapl* | chr14:34707799 | Intron | -3.162029739 | 7.84E-12 | 1.41E-11 | down |
| *Gm20707* | chr4:147800902 | Intron | -3.170812416 | 1.07E-14 | 2.14E-14 | down |
| *Ccdc138* | chr10:58566940 | Intron | -3.171597285 | 1.10E-19 | 2.59E-19 | down |
| *Aff1* | chr5:103743653 | Intron | -3.171607632 | 8.51E-53 | 3.83E-52 | down |
| *Mpdz* | chr4:81407257 | Intron | -3.191345821 | 3.46E-11 | 6.06E-11 | down |
| *AC154452.1* | chr14:16347536 | Intron | -3.21359144 | 9.90E-24 | 2.64E-23 | down |
| *A630001G21Rik* | chr1:85734327 | Intron | -3.222910243 | 2.03E-10 | 3.46E-10 | down |
| *Lrp6* | chr6:134563362 | Intron | -3.228047409 | 2.03E-20 | 4.87E-20 | down |
| *Mdm4* | chr1:132985822 | Intron | -3.228489634 | 1.29E-11 | 2.30E-11 | down |
| *Grb10* | chr11:11953327 | Intron | -3.233054064 | 6.95E-23 | 1.81E-22 | down |
| *B530045E10Rik* | chr10:99343184 | Intron | -3.24911495 | 8.03E-15 | 1.62E-14 | down |
| *Gm45895* | chr8:95917987 | Intron | -3.250164936 | 2.02E-16 | 4.28E-16 | down |
| *Gm10687* | chr9:44128633 | Intron | -3.261907159 | 3.99E-30 | 1.29E-29 | down |
| *Lipc* | chr9:70904614 | Intron | -3.277677259 | 4.82E-14 | 9.43E-14 | down |
| *Tex11* | chrX:100863524 | Intron | -3.277856415 | 5.42E-08 | 8.42E-08 | down |
| *Asph* | chr4:9495797 | Intron | -3.296390944 | 3.44E-20 | 8.18E-20 | down |
| *4932414N04Rik* | chr2:68700104 | Intron | -3.296454179 | 5.10E-12 | 9.29E-12 | down |
| *Adarb2* | chr13:8581624 | Intron | -3.299053412 | 1.50E-21 | 3.74E-21 | down |
| *Sema3c* | chr5:17611242 | Intron | -3.300317722 | 1.59E-13 | 3.05E-13 | down |
| *Gfra2* | chr14:70975480 | Intron | -3.306941934 | 1.49E-12 | 2.76E-12 | down |
| *Exd2* | chr12:80493053 | Intron | -3.309330106 | 4.31E-15 | 8.78E-15 | down |
| *Sgsm2* | chr11:74872660 | Intron | -3.316773688 | 6.69E-21 | 1.62E-20 | down |
| *Erich1* | chr8:14070075 | Intron | -3.326360716 | 2.29E-11 | 4.03E-11 | down |
| *Pgm5* | chr19:24728550 | Intron | -3.328404957 | 5.16E-17 | 1.12E-16 | down |
| *Dleu2* | chr14:61642120 | Intron | -3.332389213 | 1.85E-14 | 3.67E-14 | down |
| *Vav3* | chr3:109461855 | Intron | -3.335679389 | 3.41E-14 | 6.71E-14 | down |
| *Lhfp* | chr3:53114217 | Intron | -3.344931637 | 1.34E-32 | 4.61E-32 | down |
| *Tcl1* | chr12:105219877 | Intron | -3.349682539 | 2.27E-18 | 5.11E-18 | down |
| *Spag16* | chr1:69967232 | Intron | -3.350732022 | 2.79E-14 | 5.52E-14 | down |
| *BB557941* | chr2:57170394 | Intron | -3.35271843 | 5.40E-20 | 1.28E-19 | down |
| *Nexn* | chr3:152259569 | Intron | -3.356627312 | 1.76E-12 | 3.23E-12 | down |
| *Greb1l* | chr18:10488656 | Intron | -3.359501954 | 8.31E-15 | 1.68E-14 | down |
| *Mug-ps1* | chr6:122194605 | Intron | -3.360021861 | 4.65E-19 | 1.07E-18 | down |
| *Nrf1* | chr6:30081222 | Intron | -3.364424398 | 3.12E-14 | 6.16E-14 | down |
| *Wnk2* | chr13:49083473 | Intron | -3.384409735 | 2.15E-13 | 4.10E-13 | down |
| *Mdga2* | chr12:66748287 | Intron | -3.396686967 | 1.81E-18 | 4.10E-18 | down |
| *Mtus2* | chr5:148286118 | Intron | -3.400831323 | 3.77E-27 | 1.12E-26 | down |
| *Gm8909* | chr17:36166279 | Intron | -3.401588771 | 7.43E-14 | 1.45E-13 | down |
| *Fam169b* | chr7:68295605 | Intron | -3.406300129 | 9.98E-19 | 2.29E-18 | down |
| *Chchd6* | chr6:89532933 | Intron | -3.412825246 | 5.74E-16 | 1.20E-15 | down |
| *Fam227a* | chr15:79639450 | Intron | -3.413746531 | 5.51E-31 | 1.83E-30 | down |
| *Babam2* | chr5:31990313 | Intron | -3.438248306 | 2.17E-27 | 6.47E-27 | down |
| *Gga1* | chr15:78877320 | exon | -3.441388534 | 5.12E-13 | 9.66E-13 | down |
| *Slc39a6* | chr18:24581408 | Intron | -3.449029934 | 1.20E-15 | 2.48E-15 | down |
| *Fmn1* | chr2:113565065 | Intron | -3.460152283 | 2.61E-17 | 5.72E-17 | down |
| *Fras1* | chr5:96488592 | Intron | -3.460584485 | 1.81E-13 | 3.47E-13 | down |
| *Yes1* | chr5:32630304 | Intron | -3.464238701 | 6.46E-15 | 1.31E-14 | down |
| *Scp2* | chr4:108108799 | Intron | -3.466978626 | 1.13E-25 | 3.23E-25 | down |
| *Nell2* | chr15:95309887 | Intron | -3.480332553 | 1.84E-16 | 3.92E-16 | down |
| *Gm26903* | chr9:75974398 | Intron | -3.484307521 | 2.63E-18 | 5.91E-18 | down |
| *Fbxo47* | chr11:97861474 | Intron | -3.48436709 | 1.24E-13 | 2.40E-13 | down |
| *Snx29* | chr16:11324579 | Intron | -3.485903438 | 1.76E-40 | 6.95E-40 | down |
| *Sgip1* | chr4:102878706 | Intron | -3.499512898 | 1.59E-14 | 3.18E-14 | down |
| *Nav3* | chr10:109944060 | Intron | -3.517910128 | 1.42E-18 | 3.21E-18 | down |
| *Pacsin3* | chr2:91257692 | Intron | -3.519136642 | 5.42E-17 | 1.18E-16 | down |
| *Slc16a9* | chr10:70255198 | Intron | -3.526926965 | 3.38E-12 | 6.18E-12 | down |
| *Arhgap15* | chr2:43810939 | Intron | -3.53464462 | 1.72E-25 | 4.92E-25 | down |
| *Ttn* | chr2:76805159 | Intron | -3.541124091 | 4.34E-41 | 1.73E-40 | down |
| *Eci2* | chr13:35025465 | Intron | -3.544569284 | 1.18E-17 | 2.59E-17 | down |
| *Ttc23* | chr7:67720626 | Intron | -3.551893004 | 1.62E-13 | 3.10E-13 | down |
| *Zfp874a* | chr13:67431059 | Intron | -3.560775802 | 3.36E-13 | 6.38E-13 | down |
| *Dok6* | chr18:89635884 | Intron | -3.573625751 | 9.46E-20 | 2.22E-19 | down |
| *Slain2* | chr5:72934345 | Intron | -3.576533038 | 1.28E-11 | 2.29E-11 | down |
| *Gm26804* | chr8:20308070 | Intron | -3.577356548 | 7.13E-10 | 1.19E-09 | down |
| *Gbp8* | chr5:105022959 | Intron | -3.584979766 | 1.33E-15 | 2.73E-15 | down |
| *Dbnl* | chr11:5798714 | Intron | -3.58593978 | 1.30E-15 | 2.69E-15 | down |
| *Cntn3* | chr6:102576016 | intergenic(10kb) | -3.586665025 | 1.39E-18 | 3.16E-18 | down |
| *Cttnbp2* | chr6:18393110 | Intron | -3.586920299 | 2.10E-19 | 4.91E-19 | down |
| *Dnase1l1* | chrX:74275681 | Intron | -3.592285113 | 7.99E-16 | 1.66E-15 | down |
| *Gm6209* | chr3:50543700 | Intron | -3.593800505 | 3.96E-11 | 6.91E-11 | down |
| *Lcor* | chr19:41504620 | Intron | -3.594160276 | 1.22E-26 | 3.59E-26 | down |
| *Gtf2f2* | chr14:75936617 | Intron | -3.598718969 | 1.39E-21 | 3.49E-21 | down |
| *Alg8* | chr7:97372045 | Intron | -3.601371703 | 1.80E-14 | 3.59E-14 | down |
| *Gm43915* | chr6:120988752 | Intron | -3.601415802 | 5.28E-16 | 1.11E-15 | down |
| *Nox1* | chrX:134187368 | Intron | -3.603661362 | 6.80E-22 | 1.72E-21 | down |
| *Cask* | chrX:13721842 | Intron | -3.60592753 | 2.87E-16 | 6.04E-16 | down |
| *Padi1* | chr4:140825657 | Intron | -3.606224463 | 1.49E-13 | 2.87E-13 | down |
| *Pde1a* | chr2:79938397 | Intron | -3.607602222 | 1.09E-22 | 2.83E-22 | down |
| *Stt3b* | chr9:115305563 | Intron | -3.609409123 | 2.78E-21 | 6.86E-21 | down |
| *Adgrl3* | chr5:81706405 | Intron | -3.617169703 | 1.39E-59 | 6.74E-59 | down |
| *Grip1* | chr10:119768476 | Intron | -3.628783889 | 1.67E-27 | 5.03E-27 | down |
| *Samsn1* | chr16:75954095 | Intron | -3.631762263 | 4.52E-21 | 1.11E-20 | down |
| *9530059O14Rik* | chr9:122663130 | Intron | -3.632469408 | 1.54E-12 | 2.84E-12 | down |
| *4930480K23Rik* | chr14:69750146 | Intron | -3.632899827 | 5.34E-17 | 1.16E-16 | down |
| *Clec10a* | chr11:70158315 | Intron | -3.658556467 | 3.76E-24 | 1.01E-23 | down |
| *Med6* | chr12:81584795 | Intron | -3.664129277 | 5.45E-21 | 1.33E-20 | down |
| *Adcy10* | chr1:165529409 | Intron | -3.667866039 | 2.26E-15 | 4.64E-15 | down |
| *Dlgap1* | chr17:70730330 | Intron | -3.679926329 | 1.50E-72 | 8.24E-72 | down |
| *Mgme1* | chr2:144271561 | Intron | -3.684683666 | 9.54E-23 | 2.48E-22 | down |
| *Cdyl* | chr13:35695050 | Intron | -3.685920128 | 9.47E-28 | 2.90E-27 | down |
| *Dab1* | chr4:104588306 | Intron | -3.68710579 | 1.13E-27 | 3.42E-27 | down |
| *Gm29266* | chr14:60565797 | Intron | -3.691686829 | 1.59E-15 | 3.27E-15 | down |
| *Fnbp4* | chr2:90757070 | Intron | -3.703912927 | 3.45E-22 | 8.83E-22 | down |
| *Scfd2* | chr5:74505322 | Intron | -3.70761939 | 7.42E-17 | 1.60E-16 | down |
| *1700007G11Rik* | chr5:98396053 | Intron | -3.715327598 | 5.72E-17 | 1.24E-16 | down |
| *Susd1* | chr4:59343525 | Intron | -3.731935541 | 1.40E-22 | 3.61E-22 | down |
| *Osbpl10* | chr9:115081098 | Intron | -3.737631163 | 8.90E-36 | 3.24E-35 | down |
| *Pank1* | chr19:34866004 | Intron | -3.738921796 | 2.29E-19 | 5.35E-19 | down |
| *Lpp* | chr16:24557715 | Intron | -3.750059819 | 2.11E-23 | 5.59E-23 | down |
| *Adamtsl1* | chr4:85893614 | Intron | -3.763659322 | 3.40E-11 | 5.96E-11 | down |
| *Wdr70* | chr15:7914966 | Intron | -3.776722192 | 3.34E-18 | 7.47E-18 | down |
| *Armc9* | chr1:86212438 | Intron | -3.784250817 | 1.27E-17 | 2.80E-17 | down |
| *Akap6* | chr12:52713989 | Intron | -3.802807222 | 7.41E-26 | 2.14E-25 | down |
| *Kcnc2* | chr10:112427452 | Intron | -3.804187165 | 1.54E-24 | 4.24E-24 | down |
| *Uchl1* | chr5:66683253 | Intron | -3.81352315 | 6.50E-16 | 1.35E-15 | down |
| *Fam78a* | chr2:32076390 | Intron | -3.814105881 | 1.23E-14 | 2.46E-14 | down |
| *Dnah7b* | chr1:46076099 | Intron | -3.815138105 | 2.89E-12 | 5.30E-12 | down |
| *Catspere1* | chr1:177933862 | Intron | -3.823609569 | 3.73E-16 | 7.84E-16 | down |
| *Arntl* | chr7:113268346 | Intron | -3.828954045 | 4.36E-26 | 1.26E-25 | down |
| *Mthfd1* | chr12:76258127 | Intron | -3.83732529 | 3.05E-20 | 7.28E-20 | down |
| *Tmem232* | chr17:65333260 | Intron | -3.840592245 | 8.70E-25 | 2.43E-24 | down |
| *Prdm10* | chr9:31375629 | Intron | -3.840698712 | 1.44E-20 | 3.47E-20 | down |
| *Gm29865* | chr3:131932517 | Intron | -3.841275675 | 1.09E-21 | 2.73E-21 | down |
| *Gli3* | chr13:15467373 | Intron | -3.84193058 | 9.36E-17 | 2.02E-16 | down |
| *Slc44a1* | chr4:53605417 | Intron | -3.843174685 | 1.87E-33 | 6.56E-33 | down |
| *Mipep* | chr14:60877202 | Intron | -3.853323138 | 1.15E-24 | 3.17E-24 | down |
| *Arhgap42* | chr9:9234967 | Intron | -3.861521628 | 3.96E-19 | 9.20E-19 | down |
| *Psmd9* | chr5:123211693 | Intron | -3.862013017 | 1.05E-12 | 1.97E-12 | down |
| *Snx14* | chr9:88403568 | Intron | -3.879616105 | 7.14E-18 | 1.58E-17 | down |
| *Prkcb* | chr7:122355827 | Intron | -3.883104838 | 2.77E-20 | 6.62E-20 | down |
| *Gm44196* | chr6:98675989 | Intron | -3.883887468 | 6.54E-15 | 1.32E-14 | down |
| *Ccdc146* | chr5:21343935 | Intron | -3.885043688 | 1.07E-29 | 3.45E-29 | down |
| *Scamp1* | chr13:94218575 | Intron | -3.890118506 | 4.80E-18 | 1.07E-17 | down |
| *D630033O11Rik* | chr9:43244321 | Intron | -3.893058407 | 1.13E-09 | 1.87E-09 | down |
| *Cpne4* | chr9:104910217 | Intron | -3.895142551 | 2.09E-30 | 6.84E-30 | down |
| *Tacc2* | chr7:130663997 | Intron | -3.898147817 | 2.11E-35 | 7.67E-35 | down |
| *C77370* | chrX:104169652 | Intron | -3.905690246 | 1.48E-18 | 3.36E-18 | down |
| *Pip5k1b* | chr19:24364857 | Intron | -3.905920762 | 1.44E-28 | 4.50E-28 | down |
| *Cdk8* | chr5:146278249 | Intron | -3.907255036 | 7.22E-34 | 2.56E-33 | down |
| *Mta3* | chr17:83778476 | Intron | -3.909866511 | 1.09E-17 | 2.40E-17 | down |
| *Zhx3* | chr2:160822703 | Intron | -3.924580424 | 2.49E-15 | 5.11E-15 | down |
| *Eif2d* | chr1:131162789 | Intron | -3.928500655 | 2.34E-12 | 4.29E-12 | down |
| *1500009C09Rik* | chr15:82259350 | Intron | -3.933667449 | 5.72E-23 | 1.49E-22 | down |
| *Cpd* | chr11:76830594 | Intron | -3.940176233 | 1.19E-23 | 3.17E-23 | down |
| *Lingo2* | chr4:36154534 | Intron | -3.941063747 | 2.21E-33 | 7.73E-33 | down |
| *4930519F09Rik* | chr10:28910564 | Intron | -3.958611745 | 1.25E-15 | 2.58E-15 | down |
| *Ypel2* | chr11:86986742 | Intron | -3.973423753 | 3.83E-40 | 1.50E-39 | down |
| *Dhrs13* | chr11:78032860 | Intron | -3.973990859 | 9.49E-25 | 2.64E-24 | down |
| *Ulk2* | chr11:61817456 | Intron | -3.976199244 | 1.87E-34 | 6.67E-34 | down |
| *Arhgap1* | chr2:91665386 | Intron | -3.979435233 | 1.34E-27 | 4.05E-27 | down |
| *Ptprk* | chr10:28306752 | Intron | -3.987473907 | 1.31E-18 | 2.98E-18 | down |
| *Asah1* | chr8:41345065 | Intron | -3.987683996 | 3.00E-25 | 8.46E-25 | down |
| *Hdac9* | chr12:34069285 | Intron | -3.992472481 | 1.46E-48 | 6.36E-48 | down |
| *Bmper* | chr9:23283254 | Intron | -3.998701496 | 7.18E-19 | 1.65E-18 | down |
| *Ppfibp1* | chr6:146923299 | Intron | -4.00834231 | 5.35E-19 | 1.24E-18 | down |
| *Kifap3* | chr1:163784917 | Intron | -4.021007821 | 4.39E-26 | 1.27E-25 | down |
| *Tnni3k* | chr3:154932082 | Intron | -4.027812772 | 7.55E-33 | 2.62E-32 | down |
| *AC154826.2* | chr9:14099607 | Intron | -4.038594683 | 2.66E-17 | 5.82E-17 | down |
| *AC159261.1* | chr13:44488882 | Intron | -4.050364935 | 8.55E-22 | 2.16E-21 | down |
| *Kcnip4* | chr5:48524625 | Intron | -4.052838869 | 4.55E-37 | 1.69E-36 | down |
| *Dnah17* | chr11:118062754 | Intron | -4.059174987 | 1.19E-16 | 2.55E-16 | down |
| *4933436I20Rik* | chr1:83676565 | Intron | -4.085970797 | 2.65E-16 | 5.62E-16 | down |
| *Adam12* | chr7:133982743 | Intron | -4.097288731 | 1.85E-18 | 4.18E-18 | down |
| *Kctd16* | chr18:40308032 | Intron | -4.097916878 | 3.15E-45 | 1.33E-44 | down |
| *Vac14* | chr8:110643310 | Intron | -4.106307309 | 1.48E-20 | 3.57E-20 | down |
| *Pmfbp1* | chr8:109507240 | Intron | -4.112861888 | 1.01E-27 | 3.08E-27 | down |
| *Sfi1* | chr11:3140431 | Intron | -4.13154148 | 8.79E-29 | 2.78E-28 | down |
| *Fstl5* | chr3:76452923 | Intron | -4.136222396 | 1.63E-32 | 5.59E-32 | down |
| *Sestd1* | chr2:77226435 | Intron | -4.13846164 | 5.27E-24 | 1.41E-23 | down |
| *Spata5* | chr3:37441013 | Intron | -4.147984309 | 3.53E-23 | 9.31E-23 | down |
| *Dennd2c* | chr3:103138453 | Intron | -4.149020738 | 1.35E-64 | 6.91E-64 | down |
| *Lnpep* | chr17:17555699 | Intron | -4.17271902 | 3.35E-24 | 9.02E-24 | down |
| *Actn1* | chr12:80197693 | Intron | -4.184117981 | 1.65E-32 | 5.62E-32 | down |
| *Ranbp17* | chr11:33371154 | Intron | -4.188498501 | 2.21E-32 | 7.55E-32 | down |
| *1700025G04Rik* | chr1:151868320 | Intron | -4.190457713 | 4.06E-41 | 1.63E-40 | down |
| *Rbpj* | chr5:53540903 | Intron | -4.214545963 | 7.25E-23 | 1.89E-22 | down |
| *Pot1a* | chr6:25782292 | Intron | -4.220498979 | 1.65E-20 | 3.96E-20 | down |
| *Lrig2* | chr3:104451917 | Intron | -4.221990355 | 9.95E-18 | 2.20E-17 | down |
| *Hs3st5* | chr10:36640548 | Intron | -4.233712223 | 6.21E-12 | 1.13E-11 | down |
| *Abtb2* | chr2:103619164 | Intron | -4.237565591 | 7.09E-21 | 1.72E-20 | down |
| *Wbp11* | chr6:136826454 | Intron | -4.247961735 | 8.81E-27 | 2.60E-26 | down |
| *Fam13b* | chr18:34479120 | Intron | -4.251045886 | 1.31E-42 | 5.34E-42 | down |
| *AC158596.1* | chr10:44743361 | Intron | -4.251737891 | 2.10E-24 | 5.71E-24 | down |
| *Usp7* | chr16:8767162 | Intron | -4.255570985 | 1.11E-39 | 4.33E-39 | down |
| *Gm44115* | chr6:148705709 | Intron | -4.260540505 | 3.24E-24 | 8.73E-24 | down |
| *Galnt13* | chr2:54896192 | Intron | -4.275429164 | 5.98E-32 | 2.02E-31 | down |
| *2010110K18Rik* | chr18:34758198 | Intron | -4.275957202 | 1.01E-30 | 3.31E-30 | down |
| *Ssh2* | chr11:77248096 | Intron | -4.280755247 | 4.91E-22 | 1.24E-21 | down |
| *Coro2b* | chr9:62448380 | Intron | -4.294237608 | 2.35E-29 | 7.53E-29 | down |
| *Sv2b* | chr7:75183238 | Intron | -4.307432164 | 1.22E-24 | 3.38E-24 | down |
| *Syne3* | chr12:104949597 | Intron | -4.318153121 | 1.99E-22 | 5.12E-22 | down |
| *Rit2* | chr18:31255638 | Intron | -4.346192576 | 1.80E-33 | 6.36E-33 | down |
| *Gskip* | chr12:105689057 | Intron | -4.363444157 | 7.76E-28 | 2.38E-27 | down |
| *Gnb1* | chr4:155502918 | Intron | -4.365584372 | 1.98E-22 | 5.09E-22 | down |
| *Kat2b* | chr17:53659526 | Intron | -4.378704758 | 3.07E-19 | 7.15E-19 | down |
| *Thada* | chr17:84350047 | Intron | -4.409846891 | 7.64E-45 | 3.20E-44 | down |
| *Gm6639* | chr3:35653604 | Intron | -4.412297411 | 2.70E-23 | 7.13E-23 | down |
| *Rasgrf2* | chr13:91950683 | Intron | -4.412690727 | 5.99E-23 | 1.57E-22 | down |
| *Cluap1* | chr16:3918212 | Intron | -4.422884925 | 3.15E-22 | 8.05E-22 | down |
| *Ak7* | chr12:105736623 | Intron | -4.437797736 | 1.14E-24 | 3.16E-24 | down |
| *Pcdh15* | chr10:73439883 | Intron | -4.458293489 | 6.46E-20 | 1.53E-19 | down |
| *Faf1* | chr4:109886781 | Intron | -4.459235769 | 1.27E-14 | 2.53E-14 | down |
| *Mgat5* | chr1:127283233 | Intron | -4.462908432 | 3.89E-09 | 6.32E-09 | down |
| *Cnnm2* | chr19:46873107 | Intron | -4.464720507 | 8.81E-34 | 3.12E-33 | down |
| *Snx27* | chr3:94551890 | Intron | -4.465723381 | 1.96E-23 | 5.20E-23 | down |
| *Kdm4a* | chr4:118168166 | Intron | -4.505497271 | 6.85E-28 | 2.11E-27 | down |
| *Ccdc7a* | chr8:128815254 | Intron | -4.50841404 | 2.40E-39 | 9.28E-39 | down |
| *Rcan2* | chr17:43984214 | Intron | -4.508679277 | 2.19E-26 | 6.35E-26 | down |
| *Ctnnd2* | chr15:30221288 | Intron | -4.51047527 | 3.31E-39 | 1.26E-38 | down |
| *Tmem135* | chr7:89202867 | Intron | -4.529590409 | 2.08E-27 | 6.25E-27 | down |
| *Chm* | chrX:113118510 | Intron | -4.53700074 | 2.15E-20 | 5.15E-20 | down |
| *Cntnap5b* | chr1:100135704 | Intron | -4.537687265 | 5.15E-16 | 1.08E-15 | down |
| *Pde10a* | chr17:8714584 | Intron | -4.543542856 | 4.28E-25 | 1.20E-24 | down |
| *Dpp6* | chr5:27298089 | Intron | -4.563960054 | 9.68E-38 | 3.64E-37 | down |
| *Fam20b* | chr1:156684018 | Intron | -4.566149967 | 2.10E-31 | 7.05E-31 | down |
| *Ube2h* | chr6:30287487 | Intron | -4.567506344 | 1.09E-20 | 2.64E-20 | down |
| *Gm15290* | chr13:111523708 | Intron | -4.576004142 | 2.99E-21 | 7.36E-21 | down |
| *Ccdc88a* | chr11:29440663 | Intron | -4.579691359 | 1.43E-27 | 4.33E-27 | down |
| *6030443J06Rik* | chr5:22594241 | Intron | -4.582939569 | 3.01E-14 | 5.93E-14 | down |
| *Csnk1g1* | chr9:65944456 | Intron | -4.590922815 | 2.11E-27 | 6.30E-27 | down |
| *4932438A13Rik* | chr3:36960101 | Intron | -4.597455346 | 6.25E-32 | 2.11E-31 | down |
| *AC154352.2* | chr13:70093091 | Intron | -4.623117898 | 1.01E-28 | 3.17E-28 | down |
| *Fndc3b* | chr3:27456520 | Intron | -4.628025763 | 9.81E-35 | 3.52E-34 | down |
| *Pik3cb* | chr9:99126636 | Intron | -4.636487664 | 6.88E-25 | 1.92E-24 | down |
| *Pde4d* | chr13:108517682 | Intron | -4.647800357 | 2.61E-39 | 1.00E-38 | down |
| *Pacrg* | chr17:10443190 | Intron | -4.648912926 | 2.56E-35 | 9.25E-35 | down |
| *Myo3b* | chr2:70078046 | Intron | -4.652608832 | 5.62E-46 | 2.39E-45 | down |
| *Fbln2* | chr6:91244357 | Intron | -4.655475828 | 9.58E-30 | 3.09E-29 | down |
| *Adamtsl3* | chr7:82468687 | Intron | -4.659722396 | 2.41E-25 | 6.83E-25 | down |
| *Ankrd42* | chr7:92586881 | Intron | -4.664233513 | 9.39E-29 | 2.96E-28 | down |
| *9530036O11Rik* | chr5:28593107 | Intron | -4.677956764 | 2.08E-42 | 8.45E-42 | down |
| *Grm1* | chr10:10956636 | Intron | -4.680744977 | 2.32E-18 | 5.22E-18 | down |
| *Dnah12* | chr14:26702735 | Intron | -4.692515563 | 2.78E-24 | 7.52E-24 | down |
| *Cphx3* | chr14:26198031 | Intron | -4.693997912 | 1.73E-24 | 4.74E-24 | down |
| *Slc9a3* | chr13:74126361 | Intron | -4.696559546 | 1.00E-15 | 2.08E-15 | down |
| *Antxr1* | chr6:87198962 | Intron | -4.700438123 | 4.90E-21 | 1.20E-20 | down |
| *Exoc4* | chr6:33664551 | Intron | -4.704902238 | 6.42E-28 | 1.98E-27 | down |
| *Dnm3* | chr1:162071768 | Intron | -4.716823291 | 5.52E-28 | 1.70E-27 | down |
| *Grin2a* | chr16:9870319 | Intron | -4.723711583 | 1.17E-18 | 2.67E-18 | down |
| *Ttpa* | chr4:20008025 | five_prime_utr | -4.739128084 | 8.77E-39 | 3.34E-38 | down |
| *9830132P13Rik* | chr3:127942346 | Intron | -4.751194163 | 3.59E-33 | 1.25E-32 | down |
| *Slc24a3* | chr2:145628322 | Intron | -4.782820218 | 2.28E-25 | 6.47E-25 | down |
| *Gucy2g* | chr19:55234461 | Intron | -4.786714303 | 1.16E-22 | 3.01E-22 | down |
| *Gnaq* | chr19:16351109 | Intron | -4.795538011 | 2.68E-28 | 8.33E-28 | down |
| *Ptbp3* | chr4:59538727 | Intron | -4.805098605 | 3.64E-29 | 1.16E-28 | down |
| *Pla2g16* | chr19:7587003 | three_prime_utr | -4.831135054 | 9.57E-25 | 2.66E-24 | down |
| *Kdm7a* | chr6:39187636 | Intron | -4.846650167 | 2.53E-23 | 6.69E-23 | down |
| *Gm31363* | chr5:64449766 | Intron | -4.862203226 | 9.36E-29 | 2.95E-28 | down |
| *Bcl9* | chr3:97234529 | Intron | -4.865506401 | 1.70E-29 | 5.46E-29 | down |
| *Dapk2* | chr9:66232915 | Intron | -4.87191389 | 1.60E-26 | 4.67E-26 | down |
| *Ap3b1* | chr13:94431540 | Intron | -4.878447008 | 1.49E-14 | 2.96E-14 | down |
| *Adcy2* | chr13:68897937 | Intron | -4.886904128 | 5.53E-31 | 1.83E-30 | down |
| *Mapt* | chr11:104283717 | Intron | -4.900236454 | 4.56E-55 | 2.09E-54 | down |
| *Drc3* | chr11:60387038 | Intron | -4.903500207 | 7.01E-28 | 2.15E-27 | down |
| *Gm2716* | chr8:88063293 | Intron | -4.916607294 | 9.99E-32 | 3.37E-31 | down |
| *Lrrtm4* | chr6:80380913 | Intron | -4.924786701 | 6.04E-50 | 2.67E-49 | down |
| *Itpr1* | chr6:108479660 | Intron | -4.932218706 | 1.41E-36 | 5.20E-36 | down |
| *Pdpk1* | chr17:24135876 | Intron | -4.953616619 | 3.74E-28 | 1.16E-27 | down |
| *Lnx2* | chr5:147061287 | Intron | -4.979709564 | 2.21E-24 | 6.01E-24 | down |
| *2810425M01Rik* | chr10:77516138 | exon | -4.989700763 | 1.88E-08 | 2.97E-08 | down |
| *Tpd52l1* | chr10:31406145 | Intron | -5.008655902 | 1.38E-26 | 4.04E-26 | down |
| *Gm28960* | chr1:153079883 | Intron | -5.024950563 | 1.15E-31 | 3.87E-31 | down |
| *Rims2* | chr15:39268804 | Intron | -5.031747544 | 5.39E-45 | 2.26E-44 | down |
| *Sult2a3* | chr7:14088904 | Intron | -5.037708168 | 2.76E-30 | 8.99E-30 | down |
| *Srgap1* | chr10:121944067 | Intron | -5.040732419 | 1.31E-34 | 4.70E-34 | down |
| *Cndp2* | chr18:84682156 | Intron | -5.056519972 | 5.02E-15 | 1.02E-14 | down |
| *Dip2c* | chr13:9435705 | Intron | -5.069124996 | 1.57E-24 | 4.32E-24 | down |
| *Prkd1* | chr12:50583445 | Intron | -5.080897576 | 7.57E-33 | 2.62E-32 | down |
| *Tcp11l1* | chr2:104676914 | Intron | -5.115417993 | 1.18E-25 | 3.39E-25 | down |
| *Gria2* | chr3:80744614 | Intron | -5.11976101 | 1.92E-24 | 5.25E-24 | down |
| *Calcrl* | chr2:84371719 | Intron | -5.137766149 | 5.69E-31 | 1.88E-30 | down |
| *Atp13a4* | chr16:29446760 | Intron | -5.147235207 | 9.65E-28 | 2.95E-27 | down |
| *Mak* | chr13:41061780 | Intron | -5.150090398 | 2.22E-22 | 5.71E-22 | down |
| *Gm28981* | chr1:64261550 | Intron | -5.200763228 | 1.33E-26 | 3.90E-26 | down |
| *Foxp1* | chr6:99168588 | Intron | -5.219656282 | 3.88E-44 | 1.61E-43 | down |
| *Mgat5b* | chr11:116930620 | Intron | -5.232789847 | 4.61E-27 | 1.36E-26 | down |
| *Nbea* | chr3:55976774 | Intron | -5.259546125 | 1.95E-27 | 5.87E-27 | down |
| *Patj* | chr4:98648132 | Intron | -5.324467317 | 2.96E-39 | 1.13E-38 | down |
| *Deptor* | chr15:55159746 | Intron | -5.345762597 | 8.28E-37 | 3.05E-36 | down |
| *Nrg3* | chr14:38459146 | Intron | -5.358337253 | 1.66E-28 | 5.19E-28 | down |
| *Rnf128* | chrX:139580467 | Intron | -5.361627285 | 2.82E-32 | 9.57E-32 | down |
| *Ptpn14* | chr1:189761496 | Intron | -5.373451387 | 7.56E-41 | 3.00E-40 | down |
| *9630028H03Rik* | chr2:135513043 | Intron | -5.399690744 | 1.60E-44 | 6.66E-44 | down |
| *Amotl2* | chr9:102730480 | Intron | -5.441907397 | 2.80E-52 | 1.26E-51 | down |
| *Cbfa2t2* | chr2:154490146 | Intron | -5.461543798 | 2.38E-37 | 8.86E-37 | down |
| *1700019D03Rik* | chr1:52941918 | Intron | -5.509806253 | 3.24E-36 | 1.19E-35 | down |
| *Rsph3a* | chr17:7952364 | Intron | -5.510547306 | 4.78E-25 | 1.34E-24 | down |
| *9030617O03Rik* | chr12:100802792 | Intron | -5.517471329 | 4.17E-32 | 1.41E-31 | down |
| *Cpeb3* | chr19:37151017 | Intron | -5.533714292 | 1.95E-42 | 7.94E-42 | down |
| *Cntnap5a* | chr1:115800297 | Intron | -5.549890786 | 1.11E-42 | 4.55E-42 | down |
| *Reln* | chr5:22217643 | Intron | -5.57015731 | 2.90E-25 | 8.20E-25 | down |
| *Cdkal1* | chr13:29254609 | Intron | -5.570631936 | 5.58E-61 | 2.77E-60 | down |
| *Pitpnc1* | chr11:107270567 | Intron | -5.585149135 | 5.16E-78 | 3.01E-77 | down |
| *Rfesd* | chr13:76018154 | five_prime_utr | -5.636767828 | 8.70E-22 | 2.19E-21 | down |
| *Thtpa* | chr14:55086719 | Intron | -5.653884278 | 1.23E-43 | 5.10E-43 | down |
| *Tmem132c* | chr5:127540466 | Intron | -5.675404147 | 1.42E-53 | 6.43E-53 | down |
| *Hlcs* | chr16:94278925 | Intron | -5.681179235 | 3.09E-37 | 1.15E-36 | down |
| *Hpse2* | chr19:42891296 | Intron | -5.686905921 | 3.53E-38 | 1.33E-37 | down |
| *Lin54* | chr5:100482959 | Intron | -5.761912248 | 2.91E-25 | 8.22E-25 | down |
| *Gm15155* | chrX:155993283 | Intron | -5.837524942 | 3.62E-24 | 9.73E-24 | down |
| *Phf14* | chr6:11967856 | Intron | -5.85873876 | 6.66E-41 | 2.65E-40 | down |
| *Gm33206* | chr3:29870627 | Intron | -5.930727144 | 3.95E-29 | 1.26E-28 | down |
| *Nrxn1* | chr17:90025053 | intergenic(10kb) | -6.267970969 | 2.07E-44 | 8.61E-44 | down |
| *Syne2* | chr12:75913872 | exon | -6.652505784 | 1.29E-51 | 5.77E-51 | down |
| *Iqcg* | chr16:33023147 | Intron | -6.861807888 | 2.53E-35 | 9.16E-35 | down |
| *Lrba* | chr3:86495072 | Intron | -6.896627283 | 3.30E-39 | 1.26E-38 | down |
| *Macf1* | chr4:123622665 | Intron | -7.02847037 | 1.52E-40 | 6.01E-40 | down |

NC: unchanged

**Table S2.** List of oligonucleotide primers used in this paper.

| **Name** | **Sequence (5, to 3,)** | **Application** |
| --- | --- | --- |
| m-Clpx-sg1 | GGGGACGACCTTGGACGCTC | Gene-knockout |
| m-Clpx-sg2 | CAGCTTCGCTGTCCTAAATG |  |
| m-Coro1c-sg1 | GTTCCGCCACGTCTTTGGAC |  |
| m-Coro1c-sg2 | GCCATCATCATAGAAGCGAG |  |
| m-Kank2-sg1 | GAAGGGCCACACACTGCGCA |  |
| m-Kank2-sg2 | GGTCCAGGCAGAGCTCACTC |  |
| m-Kcmf1-sg1 | GTCAGTGTGGAAATGTTTCA |  |
| m-Kcmf1-sg2 | GATGCAGTGCATATTAACAA |  |
| m-Mlip-sg1 | GATACTGGATTATCCGTCAG |  |
| m-Mlip-sg2 | GAGATCAGCCCCTCGTACTT |  |
| m-Stxbp4-sg1 | TCGTTACCTCAACTTGGCTC |  |
| m-Stxbp4-sg2 | CCCTTCTTGTAAAGCAATTC |  |
| m-Vps26a-sg1 | AACCTAGCCTTTAAGCAGCC |  |
| m-Vps26a-sg2 | TCCATACCTCAGGCGGACAT |  |
| m-Atg5-sg1 | GACCACAAGCAGCTCTGGAT |  |
| m-Atg5-sg2 | GTGAGCCTCAACCGCATCCT |  |
| h-Coro1c-sg1 | TGCAGACACGATGAGGCGAG |  |
| h-Coro1c-sg2 | CCCACTTGCCTCTATGATTA |  |
| h-Kank2-sg1 | GGATGCCCGTCGCCGTCTCG |  |
| h- Kank2-sg2 | CCACCCGGACTGTATCCACG |  |
| h-Mlip-sg1 | GACTACCAACCCATACTCAGT |  |
| h-Mlip-sg2 | GCTGCGGTGCCAATCCCCCC |  |
| h-Vps26a-sg1 | AACCTAGCCTTTAAGCAACC |  |
| h-Vps26a-sg2 | AAGCCATATGAATCTTACAT |  |
| h-Coro1b-sg1 | GAGGACACCAGTCGATGTCC |  |
| h-Coro1b-sg2 | GTGCGCATCATCGACCCCCGT |  |
| h-Coro1a-sg1 | GCACCAGGCGATGTCTAGCAC |  |
| h-Coro1a-sg2 | GACACGAACACTGCACGCA |  |
| q-h-Coro1c-F1 | ATGACATCCGGGTTTCTCGT | qRT-PCR |
| q-h-Coro1c-R1 | GTGGCCACATACTGTAGGGT |  |
| q-h-Coro1b-F1 | CCTGGACATCGACTGGTGT |  |
| q-h-Coro1b-R1 | GATGATGCCCACTCGCTTG |  |
| q-h-Coro1b-F2 | GCACCCTGACCTCATCTACA |  |
| q-h-Coro1b-R2 | CTCGAGGTTTTCTGGGTCCC |  |
| q-h-Coro1a-F1 | GTTTGTGGCCCTGATCTGTG | qRT-PCR |
| q-h-Coro1a-R1 | ATCTCCCACACCATGACTGT |  |
| q-h-Coro1a-F2 | GCACCCAGACACGATCTACAG |  |
| q-h-Coro1a-R2 | GACACGAACACTGCACGCA |  |
| q-m-Coro1c-F1 | CAGTTCCTTTTGTGCCGTCA |  |
| q-m-Coro1c-R1 | ACACCAGTCTATGTCCAGCA |  |
| q-m-Coro1b-F1 | GTTCCTGGCAGTGATTGTGG |  |
| q-m-Coro1b-R1 | GCCATACCATGACAGTGCAG |  |
| q-m-Coro1b-F2 | CCACAATGGCAGCCTCTTTT |  |
| q-m-Coro1b-R2 | TTCACTCATGCGGCTGAAAC |  |
| q-m-Coro1a-F1 | CGCGTCTCACAAACCACTT |  |
| q-m-Coro1a-R1 | ACACCAGGCGATGTCTAGC |  |
| q-m-Coro1a-F2 | CACGCTGTGTTTGTGTCAGA |  |
| q-m-Coro1a-R2 | CGGATAGAGCTGTCACCCTT |  |
| HMSpAa | CGAAGAGTAACCGTTGCTAGGAGAGACCGTG GCTGAATGAGACTGGTGTCGACACTAGTGG | Splinkerette PCR |
| HMSpBb-Sau3AI | GATCCCACTAGTGTCGACACCAGTCTCTAAT TTTTTTTTTCAAAAAAA |  |
| HMSp1 | CGAAGAGTAACCGTTGCTAGGAGAGACC |  |
| HMSp2 | GTGGCTGAATGAGACTGGTGTCGAC |  |
| PB3,-1 | TAAATAAACCTCGATATACAGACCGATAAA |  |
| PB3,-2 | ATATACAGACCGATAAAACACATGCGTCAA |  |
| PB3,-seq | TTTTACGCATGATTATCTTTAACGTACGTC |  |
| PB5,-2 | CTTACCGCATTGACAAGCACGCCTCACGGG |  |
| PB5,-3 | CGCATGATTATCTTTAACGTACGTCAC |  |
| PB5,-seq | TTAGAAAGAGAGAGCAATATTTCAAGAATG |  |
| P1 | CTAGGTAGGGGATCGGGACT | *Rosa26* locus targeted identify |
| P2 | CTTCTCTAGGCACCGGTTCA |  |
| P3 | CATCTGTTGTTTGCCCCTCC |  |
| P4 | CAAGCACTGTCCTGTCCTCAAGGA |  |
| F1 | TCCTAAGAAACAGCCTCCTGCC | *Coro1c* genome-typing PCR |
| F2 | ACACACTGAGAACTGATCCAGGTCA |  |
| F3 | GCTGCTCTGGATCTCTAGGG |  |

**Figure S1.** Genome-wide loss-of-function screen for genes regulating autophagy in mouse haESCs. (**A**) A schematic diagram illustrating the insertion of GFP-LC3-RFP-LC3ΔG construct, driven by the *Eef1a* promoter, into the *Rosa26* locus of haESC for the generation of cell lines reporting autophagic flux. (**B**) PCR validation of the insertion of GFP-LC3-RFP-LC3ΔG into the *Rosa26* locus of haESC. (**C**) Sorting the haGRL cell line (A2 clone) in haploid state via FACS. (**D**) Schematic diagram illustrating the strategy to knock out the *atg5* gene in mouse haGRL using the CRISPR-Cas9 system. Boxes indicate the exons of the genes. (**E**) Western blot validation of ATG5 expression in the indicated cell lines. GAPDH was used as a control. (**F**) Quantification of the proportion (%) of autophagy-deficient cells in ROI in (**Figure 1D**) (n=3). (**G**) Splinkerette-PCR combined with massively parallel sequencing to identify the genome coverage and distribution rates of PB transposon in HML. (**H**) FACS analysis showed that HML contained a high proportion of haploid cells. (**I**) Homozygosity analysis of individual mutant ES cell clones. Heterozygous mutants are marked in red. M, mutant sample; W, wild-type haESC. Data are presented as mean ± SEM. Unpaired t-test for F. *** *p*<0.001.

**Figure S2.** CORO1C is a novel autophagy regulator and is essential for the formation of SQSTM1/p62 bodies. (**A**) Results of the secondary screen. The seven candidate genes were individually knocked out in AB2.2 GRL cells. Indicated cells were treated with nutrient-rich or starvation condition for 24 h, and subsequently analyzed by flow cytometry. (**B**) WT and *coro1c*-KO AB2.2 GRL cells were treated with nutrient-rich or starvation condition for 24 h, and then determined by flow cytometry. The autophagy-deficient cells in starvation condition are indicated by the ROI. (**C**) 293FT GRL cells were knocked out the four candidate genes individually. Those cells were treated with nutrient-rich or starvation condition for 24 h, and then determined by flow cytometry. (**D**) WT and *CORO1C*-KO 293FT GRL cells were treated with nutrient-rich or starvation condition for 24 h, and then determined by flow cytometry. The autophagy-deficient cells in starvation condition are indicated by the ROI. (**E**) Schematic diagram of the strategy to knock out *CORO1C* in H4 cells by using CRISPR-Cas9 system. Boxes indicate the exon of the gene. (**F**) The protein level of CORO1C in indicated cell analyzed by western blot. Cell clone No.4 was selected as the rescue clone for subsequent experiments. Data are presented as mean ± SEM. Unpaired t-test for A and C. * *p*<0.05, *** *p*<0.001, **** *p*<0.0001; ns, not significant.

**
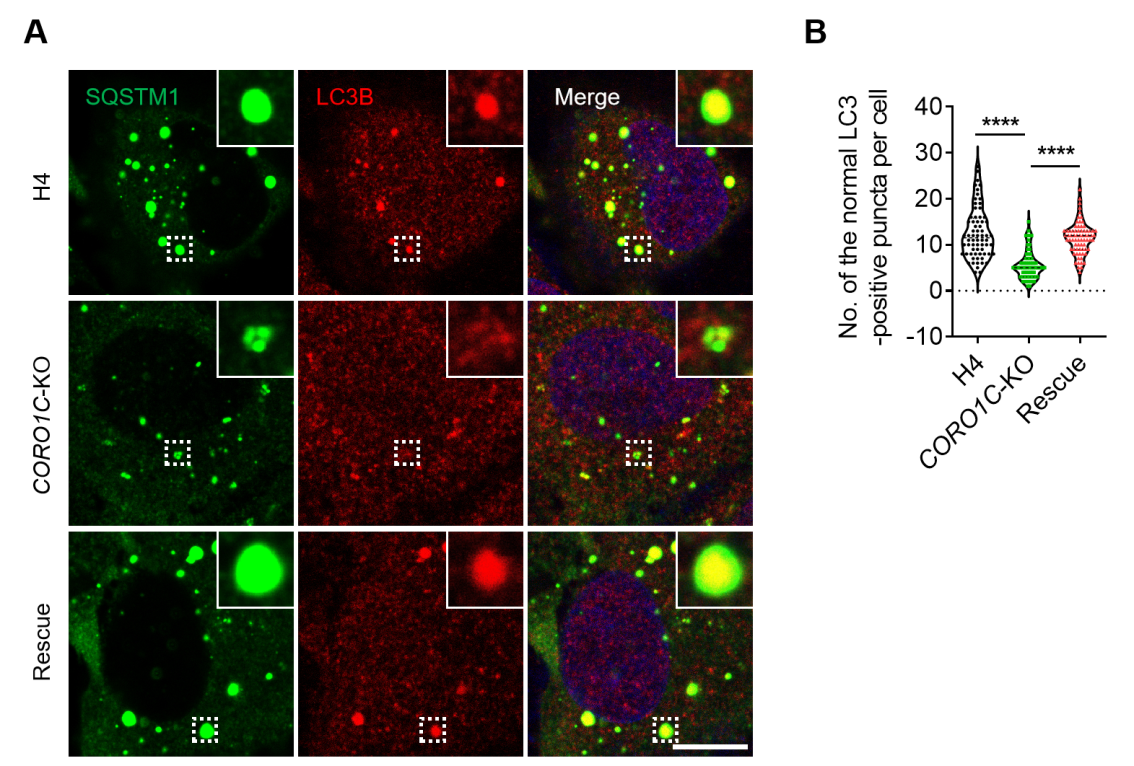
**

**Figure S3.** *CORO1C* deficiency impairs the formation of autophagosome. (**A**) Representative confocal images of WT, *CORO1C*-KO and rescued H4 cells immunolabeled with anti-SQSTM1/p62 and anti-LC3B after starved for 4 h. DAPI was used to stain the nucleus (blue). Regions outlined with white dashed lines are magnified in the insets. Scale bar: 10 μm. (**B**) Cells from (**A**) were quantified for normal LC3-positive puncta. n=68 (H4), 71 (*CORO1C*-KO) and 72 (Rescue) cells were assessed from three independent experiments. Data are presented as mean ± SEM. Unpaired t-test for B, **** *p*<0.0001.


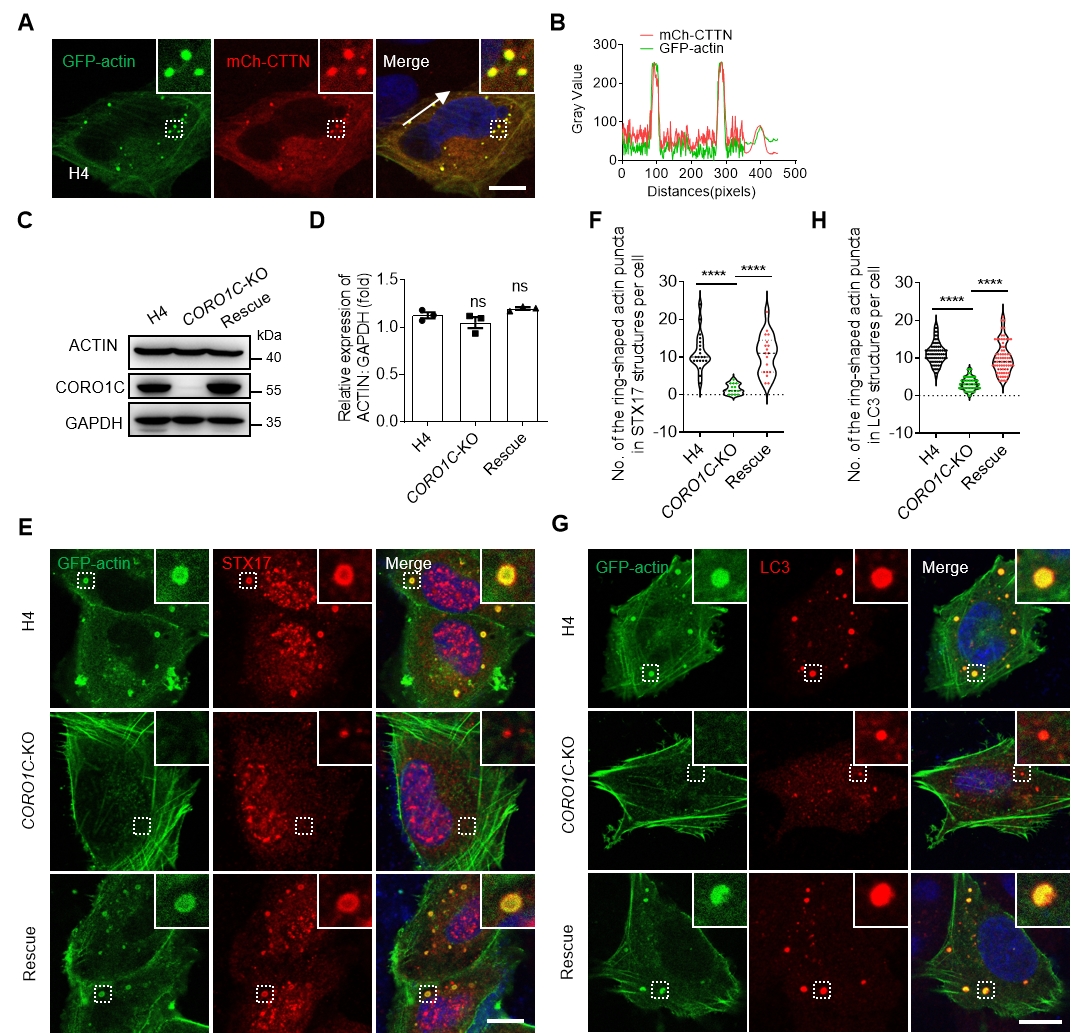


**Figure S4.** CORO1C facilitates the formation of the branched actin network in autophagy-related structures. (**A**) Representative confocal images of WT H4 cells transfected with GFP-actin and mCherry-CTTN. DAPI was used to stain the nucleus (blue). Regions outlined with white dashed lines are magnified in the insets. Scale bar: 10 μm. (**B**) Fluorescent intensity of GFP-actin and mCherry-CTTN signals indicated region marked with arrow from (**A**) is shown in line plots. Scale bar: 10 μm. (**C**) WT, *CORO1C*-KO and rescued H4 cells were treated in nutrient-rich condition, and then the cell lysates were analyzed by western blot. (**D**) Quantification of the ratio of ACTIN:GAPDH from (**C**) (n=3). (**E**) Representative confocal images of WT, *CORO1C*-KO and rescued cells transfected with GFP-actin and immunolabeled with anti-STX17 after starved for 4 h. DAPI was used to stain the nucleus (blue). Regions outlined with white dashed lines are magnified in the insets. Scale bar: 10 μm. (**F**) The percentage of actin-positive STX17 structures was quantified in cells from (**E**). n=20 (H4), 14 (*CORO1C*-KO) and 18 (Rescue) cells were assessed from two independent experiments. (**G**) Representative confocal images of WT, *CORO1C*-KO and rescued H4 cells transfected with GFP-actin and immunolabeled with anti-LC3B after starved for 4 h. DAPI was used to stain the nucleus (blue). Regions outlined with white dashed lines are magnified in the insets. Scale bar: 10 μm. (**H**) The percentage of actin-positive LC3 structures was quantified in cells from (**G**). n=67 (H4), 60 (*CORO1C*-KO) and 72 (Rescue) cells were assessed from two independent experiments. Data are presented as mean ± SEM. Unpaired t-test for D, F and H. **** *p*<0.0001; ns, not significant.

**
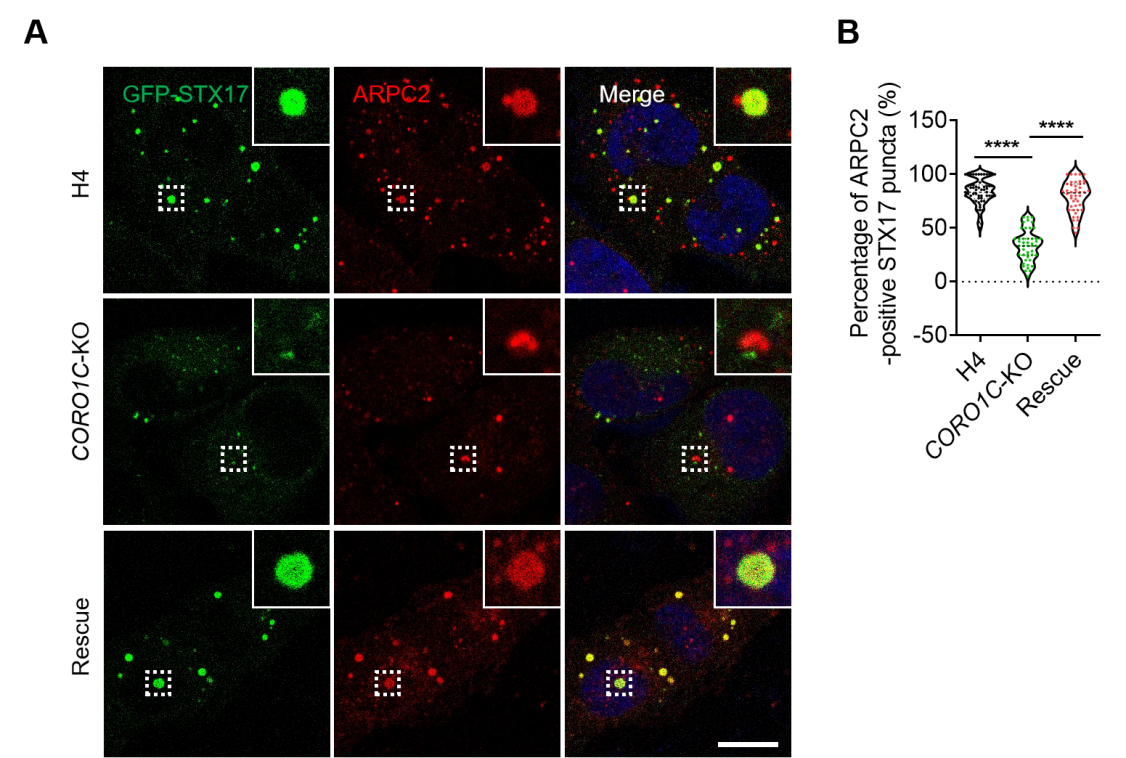
**

**Figure S5.** CORO1C facilitates the formation of the branched actin network in autophagy-related structures. (**A**) Representative confocal image of WT, *CORO1C*-KO and rescued H4 cells were transfected with GFP-STX17 and immunolabeled with anti-ARPC2 after starved for 4 h. DAPI was used to stain the nucleus (blue). Regions outlined with white dashed lines are magnified in the insets. Scale bar: 10 μm. (**B**) Cells from (**A**) were quantified for ARPC2-positive STX17 puncta. n=58 (H4), 46 (*CORO1C*-KO) and 46 (Rescue) cells were assessed from three independent experiments. Data are presented as mean ± SEM. Unpaired t-test for B. **** *p*<0.0001.

**Figure S6.** CORO1C, but not CORO1A or CORO1B, regulates autophagy. (**A, B**) Expression level of CORO1C, CORO1B and CORO1A in H4, 293FT (**A**), haESC and AB2.2 (**B**) cells tested by RT-qPCR. The expression level was normalized by GAPDH (n=3). (**C, D**) Expression level of CORO1C, CORO1B and CORO1A in H4 (**C**), 293FT (**D**) cells and indicated KO cells tested by RT-qPCR. The expression level was normalized by GAPDH (n=3). (**E**) Western blot analysis of indicated protein expression in WT, *CORO1C*-KO, *CORO1B*-KO and *CORO1A*-KO 293FT cells cultures in nutrient-rich or starvation conditions for 4 h. (**F-H**) Quantitation of protein signal intensities from western blot in (**E**) showing the ratio of SQSTM1:GAPDH (**F**), LC3-ⅠⅠ:LC3-Ⅰ (**G**) and LC3-ⅠⅠ:GAPDH (**H**) (n=3). (**I**) Sanger sequence of the different types of rescue plasmid. (**J**) WT, *CORO1C*-KO, and indicated rescued cells were subjected to either nutrient-rich or starvation conditions for 4 h. Subsequently, western blot was performed to analyze the level of indicated protein. Data are presented as mean ± SEM. Unpaired t-test for F and G. ***p*<0.01, ****p*<0.001; ns, not significant.

**Figure S7.** The *coro1c^-/-^* mice exhibit autophagic defects and spatial learning memory impairment. (**A**) Sanger sequence of the *coro1c^-/-^* mice. (**B**) Proportion of mice with different genotypes at birth. (**C**) Photograph of a representative *coro1c^-/-^* and WT littermate at birth. (**D**) Photograph of a representative *coro1c^-/-^* and WT littermate at 10 weeks age. (**E, F**) Plasma and tissue amino acid concentrations. Amino acid concentrations were measured at 0 h (**E**) and 10 h (**F**) after the caesarean delivery under fasting conditions. “Total” indicates the sum of the Asp, Thr, Ser, Asn, Glu, Gln, Pro, Gly, Ala, Val, Cys, Met, Ile, Leu, Tyr, Phe, Lys, His and Arg concentrations; “Essential” indicates the sum of Thr, Val, Met, Ile, Leu, Phe, Lys, His and Arg concentrations; “BCAA” indicates the sum of the Val, Ile and Leu concentrations. Tissue amino acid concentrations are expressed as mmol kg-1 of wet weight. (**G-I**) Quantification of total distance traveled (**G**), the movement speed (**H**) and the number of entries into the center (**I**) of control and KO mice in the open field test. (**J**) Time spent in the novel arm during the Y-maze test (n=5 in KO and n=13 in Ctrl group). (**K**) Representative western blot of indicated protein content in the brain from WT and *coro1c^-/-^* mice under fed or fast (24 h) conditions. Data are presented as mean ± SEM. Unpaired t-test for E, F, G, H, I and J; ** *p*<0.01; ns, not significant.

**Figure S8.** Coronin family in different species. (**A**) The results of protein BLAST between yeast Crn1 and human type 1 coronin (CORO1A, CORO1B, CORO1C). (**B, C**) The number of amino acids in coronins from zebrafish to mouse to human. Z, Zebrafish; M, Mouse; H, Human.
